# 2-Nitro- and 4-fluorocinnamaldehyde based receptors as naked-eye chemosensors to potential molecular keypad lock

**DOI:** 10.1038/s41598-021-99599-w

**Published:** 2021-10-21

**Authors:** Muhammad Islam, Zahid Shafiq, Fazal Mabood, Hakikulla H. Shah, Vandita Singh, Muhammad Khalid, Sara Figueirêdo de Alcântara Morais, Ataualpa Albert Carmo Braga, Muhammad Usman Khan, Javid Hussain, Ahmed Al-Harrasi, Najat Marraiki, Nouf S. S. Zaghloul

**Affiliations:** 1 Institute of Chemical Sciences, Bahauddin Zakariya University, Multan, 60800 Pakistan; 2 Natural and Medical Sciences Research Centre, University of Nizwa, P. O. Box 33, Birkat Al Mauz, Nizwa 616, Nizwa, Oman; 3 Department of Food Science and Human Nutrition, College of Applied and Health Sciences, A’Sharqiyah University, P. O. Box 42, Ibra, Oman; 4 Department of Chemistry, Khwaja Fareed University of Engineering & Information Technology, Rahim Yar Khan, 64200 Pakistan; 5 Departamento de Química Fundamental, Instituto de Química, Universidade de São Paulo, Avenida Professor LineuPrestes, 748, São Paulo, 05508-000 Brazil; 6 Department of Chemistry, University of Okara, Okara, 56300 Pakistan; 7 Department of Botany and Microbiology, College of Science, King Saud University, P.O. 2455, Riyadh, 11451 Saudi Arabia; 8 Bristol Centre for Functional Nanomaterials, HH Wills Physics Laboratory, University of Bristol, Tyndall Avenue, Bristol, BS8 1FD UK; 9 Institute of Chemical Sciences, University of Swat, Swat, Pakistan

**Keywords:** Environmental sciences, Natural hazards, Chemistry, Mathematics and computing

## Abstract

New-generation chemosensors desire small organic molecules that are easy to synthesise and cost-effective. As a new interdisciplinary area of research, the integration of these chemosensors into keypad locks or other advanced communication protocols is becoming increasingly popular. Our lab has developed new chemosensor probes that contain 2-nitro- (**1–3**) and 4-fluoro-cinnamaldehyde (**4–6**) and applied them to the anion recognition and sensing process. Probes **1–6** are colorimetric sensors for naked-eye detection of AcO^−^/CN^−^/F^−^, while probes **4–6** could differentiate between F^−^ and AcO^−^/CN^−^ anions in acetonitrile. Using the density functional theory (DFT), it was found that probes **1–6** acted as effective chemosensors. By using Probe **5** as a chemosensor, we explored colorimetric recognition of multiple anions in more detail. Probe **5** was tested in combination with a combinatorial approach to demonstrate pattern-generation capability and its ability to distinguish among chemical inputs based on concentration. After pattern discrimination using principal component analysis (PCA), we examined anion selectivity using DFT computation. In our study, probe **5** demonstrates excellent performance as a chemosensor and shows promise as a future molecular-level keypad lock system.

## Introduction

Rationally designed molecular systems that create new functional materials are of fundamental interest for application in molecular electronics^1–7^, opto-electronics^7–10^, chemo-sensing^11,12^, and information security systems^13,14^. Many organic molecules with light-emitting and light-absorbing properties have been used to build chemical logic devices^15,16^. The advantage of chemical logic devices over conventional devices are their sensitivity and specificity to chemical structure and their ability to respond to unconventional input signals^17^. A chemical logic system could be activated only by a specific chemical input. The encryption protects them from detection, tampering, and cracking. Chemical logic gate research has made strides ever since de Silva and coworkers made their major breakthrough^18–22^. In the field of chemosensors, there has been a rapid evolution from very simple chemical applications to molecular logic computation systems. Chemistry-based logic sensors are now capable of processing chemical information in the same way as electronic logic devices^19^. Based on chemical systems, a number of molecular switches, logic gates, and logic circuits have been developed in the past decade^14,15^.

The idea of selective detection, low production costs, and ease of processing has always been central to the success of the research. Organic molecules provide these qualities; however, they display some limitations such as poor solubility in aqueous media, non-specific fluorescence quenching caused by electron transfer mechanisms, heavy transition metals, and spin–orbit coupling, which need to be eliminated^23^. Small organic molecules have been successfully employed in colorimetric sensing of medically and environmentally important ions and neutral species. It offers onsite and short-time detection, simplicity, selectivity, sensitivity, and reversibility along with minimal or no sample preparation and manual expertise. These chemosensors are increasingly getting popular in chemical logic devices^17^.

Recent reports describe the application of thiosemicarbazone-based sensors in host–guest interactions and Molecular Logic Gates^24^. Hydrazone-based sensor molecules have been used in optical molecular switches and sensors for biologically important ions and molecules^25,26^. There are also pyrrole/indole and phenyl/hydroxyl based sensors which have been proposed for molecular keypad locks and as a molecular switch^27^. Moreover, calyx-4-pyrrole-based sensors have been reported for pattern generation and recognition sensing for application as potential molecular keypad locks^15^. These system has added advantage of miniaturization and potential for functional group diversification. There is scope for exploring the pattern-generating molecules to personalized medicine for specific targeted diagnosis and therapy as in the logic-based therapy and they are not confined to sensing and molecular computing^28^. Moreover, the integration of these anion sensor molecules into molecular level keypad lock devices has become a dynamic area of research^29–31^.

Our interest in substituted thiosemicarbazones is because chemosensors designed with C=S and N–H functional groups have been shown to be efficient^30^. As well as their low-cost, unique sensing potential for fluoride and cyanide^32^, and the ability to tune their conjugation via functional group diversification. Recently, we reported improvements in detection limits and binding constant values by optimizing the substituents on the thiosemicarbazides^33^. Two new chemosensor systems were developed keeping in view the unique properties of nitro- and fluoro- substituted molecules: 2-nitro-thiosemicarbazides (**1–3**) and 4-fluoro-based-thiosemicarbazides (**4–6**). These molecular sensors confer an advantage in terms of synthesis ease, modularity, and unique structural properties. Probes **1** through **3** and probes **4** through **6** showed an intense change from colorless to dark red and from colorless to yellow, respectively, in response to fluoride ions, which could be seen by naked eye.

The present work describes the synthesis, characterization, anion sensing properties and binding energies of probes **1–6**. In our investigation, we found that **5** was able to distinguish between AcO- and F- in a particular sequential addition, as previously reported for calyx-4-pyrrole based sensors^15^. These observations led us to investigate a potential molecular keypad lock. In order to simulate molecular level keypad lock, we expect that probe **5** would be able to recognize chemical input patterns and distinguish among the concentrations of the inputs^15,34^. We tested several combinations of inputs and created a library of data. We performed principal component analysis (PCA) to distinguish the patterns generated in the experiment, and we applied DFT to investigate the chemistry. Therefore, this way of using a molecular system provides a new insight into the detection of anions, as well as novel possibilities for their integration into multifunctional logic devices, which would be of future interest for molecular computing.

## Results and discussion

The chemosensors designed with the presence of –NH and C=S functional groups on probes to increase the reactivity of the probes. The two step synthesis yielded compound **1–6** as probe (receptors) in good yield (76–88%). The structures of all the probes were characterized by with different spectroscopic techniques including FTIR, ^1^HNMR and ^13^C NMR, Mass spectrometry. The IR spectrum of probes 1–6 showed characteristics C=S stretching frequencies at 1186–1198 cm^−1^, for C=N at 1580–1590 cm^−1^ and for N–H at 3119–3219 and 3318–3334 cm^−1^. The ^1^H NMR showed two characteristics singlet peaks at 11.67–10.03 and 9.13 ppm for N–H protons.

### Colorimetric analysis and UV–visible spectral studies

**Scheme 1:**
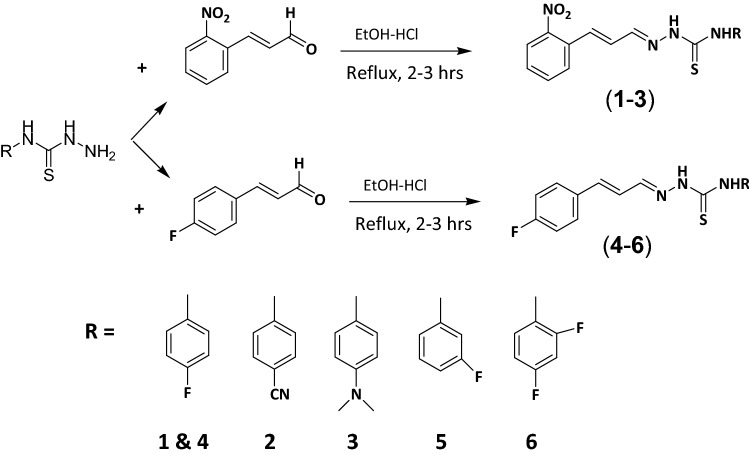
Synthesis of receptor compounds **1–6**.

**Figure 1:**
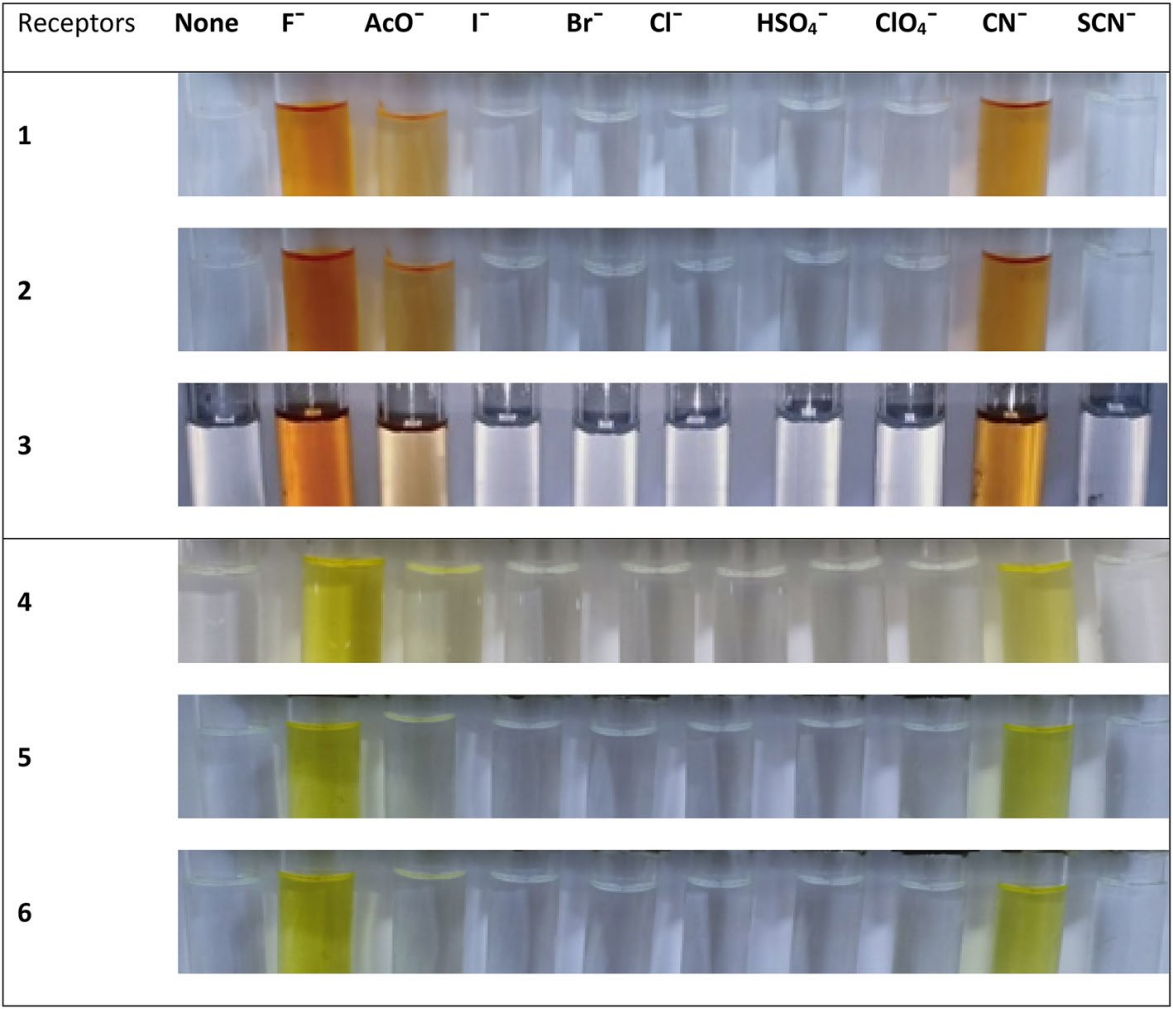
Visible colorimetric responses of probes **1**–**6** in CH_3_CN (5 × 10^−5^ M) upon addition of 30 equiv. of various anion.

The UV–visible spectra of probes **1–6** were recorded without the anion. The chromophore effect could be seen among the two groups, i.e. nitro-derivatives receptors **1–3** and fluoro-derivatives receptor **4–6**. Specifically; upon comparing the absorption maxima of **1** (318 nm) and **4** (342 nm), where the receptor unit were the same however the chromosphere unit were different. The difference calculated due to the chromophore effect was about ~ 24 nm which was attributed to π–π* and/or n–π* transitions in the two separate range groups as expected depending on the electron-withdrawing effect of substituents (Scheme 1). Initially, probes **1–6** were evaluated for anion sensing ability by the naked eye. We found that the reaction of the probe **1–6** with the anions solutions (F^−^, Cl^−^, Br^−^, I^−^, ClO_4_^−^, AcO^−^, HSO_4_^−^, CN^−^, and SCN^−^) caused a change in the color which could be seen by naked eyes. The intense color change was observed with fluoride, while less prominent with cyanide and acetate ion solutions. However, other anions did not show a visible change in color (Fig. 1). Probe **1–3** which were nitro-derivatives showed a change from colorless to dark red while the fluoro-derivatives **4–6** changed from colorless to yellow.

**Figure 2:**
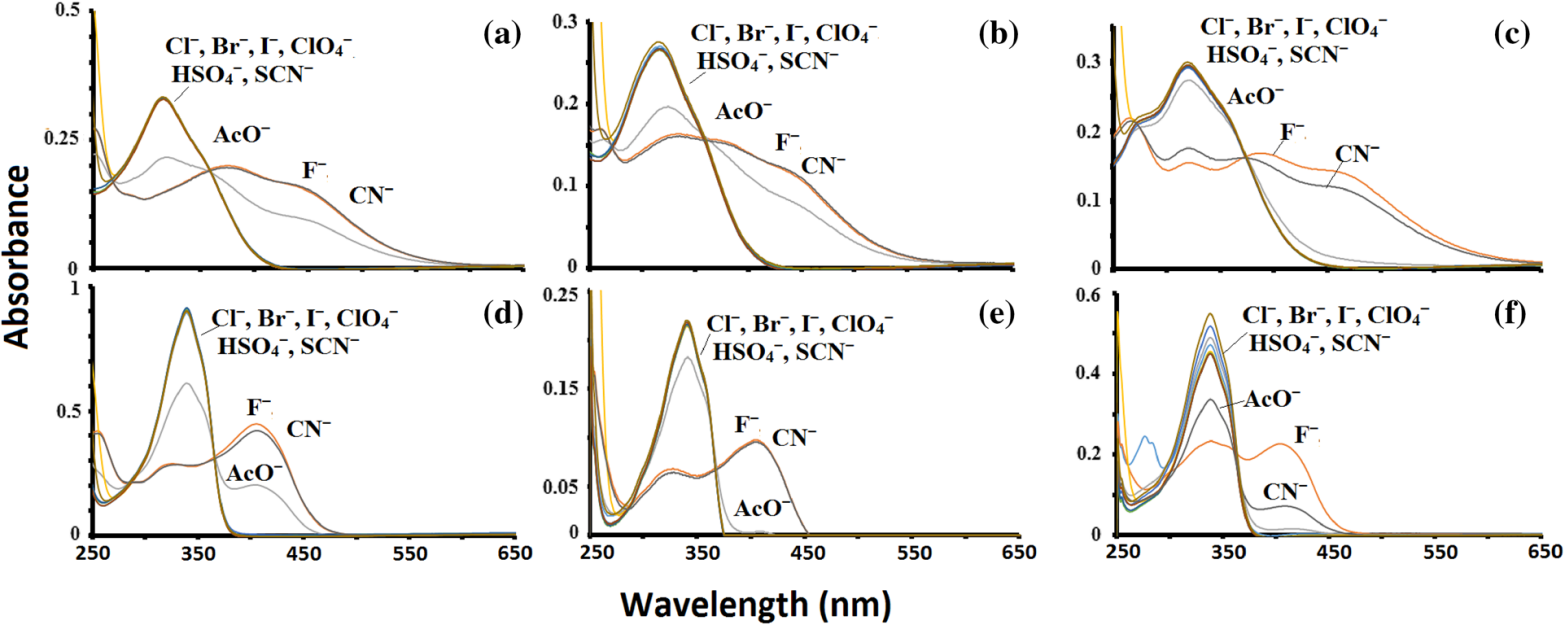
Changes in UV–vis absorption of **1–6** (5 × 10^−5^ M) upon addition of 30 equiv. of different tetra-butylammonium salts of anions in CH_3_CN solution as shown in (**a–f**) respectively.

The absorption spectrum of probes **1–6** with the anions (F^−^, Cl^−^, Br^−^, I^−^, ClO_4_^−^, AcO^−^, HSO_4_^−^, CN^−^, and SCN^−^) reveal that only fluoride and cyanide could cause obvious changes in the spectrum while some change by acetate anion. As shown in Fig. 2 new broad absorption bands between 350 and 500 nm appeared upon addition of fluoride and cyanide anions for probes **1–3** while new absorption band between 350 and 450 nm appeared after the addition of the same anions in probes **4–6**. The addition of acetate anion to probes **1–6** showed a decrease in the intensity of the ICT band to a broad shoulder band and concomitant formation of new bands similar to fluoride and cyanide anion addition, however, subtle differences were noted. These overall changes indicate anion binding, through hydrogen bonding at the electron-rich thiourea, attached to electron-withdrawing functional groups which create a push–pull effect and consequently shift the absorption wavelength towards red.

The ratiometric response of the probes **1–6** was studied by incremental addition of fluoride, acetate, and cyanide ions (1 × 10^−2^ M) to the fixed volume of receptor probes (3 ml, 3 × 10^−5^ M). During titration of receptor **1–3** with fluoride and cyanide anions the absorption maxima at ~ 320 nm progressively collapsed and the new band at ~ 245 nm and red-shift broad absorption bands around 350 and ~ 450 nm developed with isosbestic points observed at 275, 367 nm (**1**), 274, 370 nm (**2**) and 272, 380 nm (**3**) respectively, indicating a single component was produced. A large ∆λ_max_ of 122, 123, and 139 nm were observed with the change in color of the solutions from colorless to red. These observations were attributed to experimental more favorable towards ICT process (π–π* and/or n–π*) in **1–3** due to charge propagation to the electron-deficient (–NO_2_) moiety, and a significant intense color change due to extended conjugation^35^. During titration of receptors with acetate anion, we observed specific and unique changes. We noted that absorption maxima for receptor **1–3** at around 320 nm decreased slightly and new broadband appeared around 350 nm and 460 nm with significant ‘tailing off’. Thus we notice a subtle difference in the spectra of titration with acetate anion.

Titration of receptor **4–6** with fluoride and cyanide revealed that absorption maxima for **4, 5** and **6**, at 342, 343, and 339 nm collapsed and a new band at around 246 nm and a new red-shift sharp band at 400, 401 and 402 nm appeared and developed with isosbestic points at 287, 363 nm (**4**) 296, 362 nm (**5**), and 292, 359 nm (**6**) respectively with a change in color of the solutions from colorless to yellow. The ∆λ_max_ of 63, 64, and 67 nm were observed. The new red-shift band induced by fluoride anions match well with recent reports of anion sensors^36^, suggesting that it could be the response towards deprotonating of receptors **1–6** as also predicted by our theoretical calculation. For receptor **4–6** upon titration with acetate anion as noted above the absorption maxima reduced only slightly and new broad bands appear around 248 nm and at around 406 nm giving a slight yellow color solution. The spectra for **1–6** upon titration with acetate revealed only 10–20% reduction in the absorption maxima. Among all the receptors the least changes in the absorption maxima observed for receptors **3** and **5** (Fig. 2c–e).

**Figure 3:**
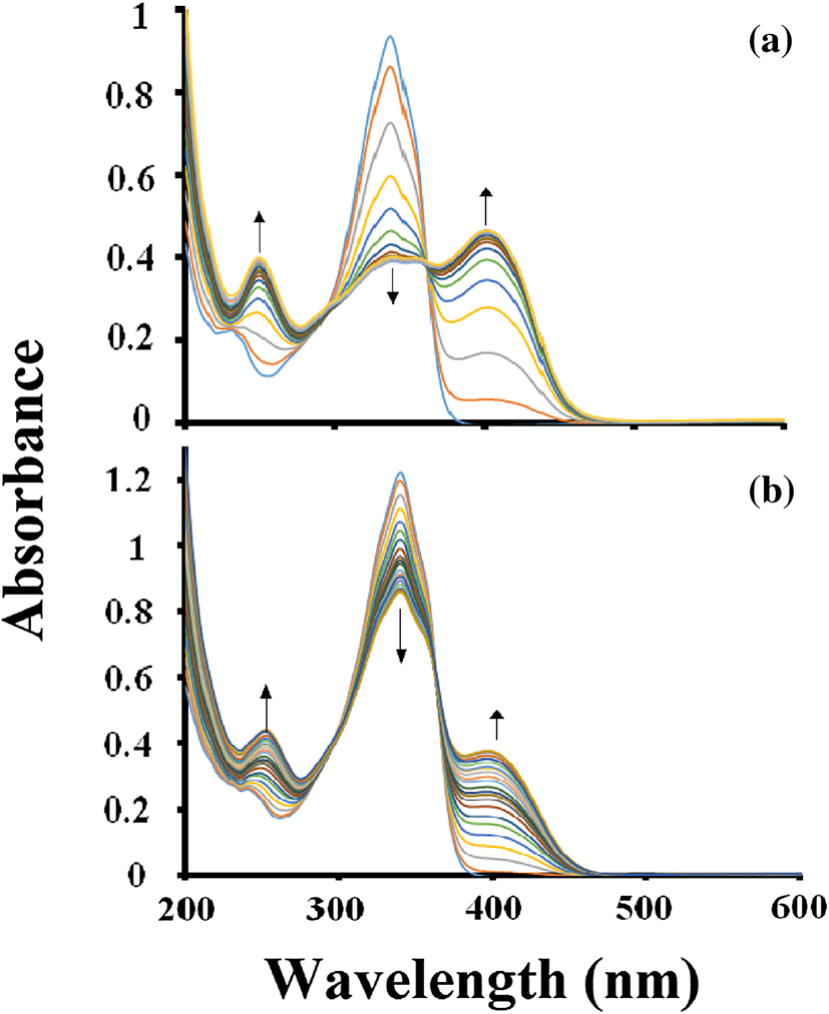
Absorption spectra for probe **5** (3 ml, 3 × 10^–5^ M) in acetonitrile solution plotted against incremental addition (0–10 equiv.). (**a**) Fluoride anions and (**b**) acetate anions.

A comparison of the fluoride and acetate anions titration plot with model receptor **5** are shown in Fig. 3. It could be seen that the absorption maxima of **5** collapsed upon titration with fluoride while the absorption maxima reduced only 20% upon titration with acetate. Hence we propose that these changes are due to the direct interaction of the acetate anion with the receptor probes in a specific and unique manner and not by a common mechanism such as deprotonation of the thiourea protons, as such a mechanism would have led to the formation of the same product for all the anions and hence, identical absorption spectrum.

The detection limit is an essential function of the anion sensor. All the receptors **1–6** were efficient sensors for fluoride anion with the detection limits in the range of 4.49 × 10^–7^ M to 2.57 × 10^–6^ M. The detection limit of 4.49 × 10^–7^ M for fluoride was much lower compared to some recent reports on chemosensors^37^. The cyanide anion detection limits was in the range of 4.78 × 10^–7^ M to 1.98 × 10^–6^ M. The detection limit of 1.98 × 10^–6^ M was much less that phenylthiourea derivatives^38^, and azo-benzyldenethiourea reported previously According to the World Health Organization, concentrations lower than 5.3 mM for fluoride are acceptable in drinking water^39^, which means that probe **1–6** are valuable tools for the detection of fluoride in safe drinking. The stoichiometry of the receptor to anion ratio was determined by taking equimolar combinations of receptors and tetra-butyl-ammonium salts of anions in CH_3_CN. The calculations were done using Job’s Plot. All the compounds show receptor: anion ratio as 1:2 for **1–6** (Fig. S2a–f). From the above titration results, the anions indeed interacted with the (N–H) protons of the semicarbazone^40^. The Benesi-Hildebrand equation was applied to calculate the association constant^41^. It was found that nitro-based receptors show a lower association constant value compared to fluoro-based receptors. Specifically, comparing **1** and **4,** their associations constant were 1.64 × 10^4^ and 1.63 × 10^5^ respectively where the receptor unit are the same only the chromophoric units are different (Table 1). Similar substituent effect have been reported recently^42^. Among the nitro-based receptors, compound** 3** has the highest association constant which could be attributed to the push effect of the tertiary amine group on the receptor. Whereas, among the fluoro-based receptors, probes **4 **and **6** had high association constants which are attributed to the para- and ortho-para position of the fluorine substituent on the receptors respectively. It was very predictable that the highest association constant would be for **6** with ortho-para difluoride substituted receptor unit which provides a greater electron-withdrawing effect. Our result are inline with our previous report of Coumarin-thiosemicarbazones and Acridine based thiosemicarbazone receptor compounds as shown in the Table 1, however, the association constant values for compound **6** are higher in the current study compared to the earlier reports. The nature of interaction between thiourea protons and the anion plays a critical role. Most likely the sensing for acetate was due to hydrogen bonding interaction with thiourea protons, however for fluoride initial hydrogen bonding interaction followed by ion-pair complex formation (bifluoride ion) HF_2_^−^, due to its deprotonating ability^43^.

**Table 1:** Binding constants and detection limit values of receptor (1–6) in acetonitrile.

Receptor	Binding constant (mol L^−1^)			Detection limit (M)			References
	F^−^	CN^−^	AcO^−^	F^−^	CN^−^	AcO^−^	
1	1.64 × 10^4^	2.62 × 10^4^	3.86 × 10^3^	3.56 × 10^–6^	3.89 × 10^–6^	4.39 × 10^–5^	This work
2	2.96 × 10^4^	3.19 × 10^4^	2.35 × 10^3^	2.18 × 10^–6^	2.39 × 10^–6^	1.36 × 10^–4^	This work
3	7.11 × 10^4^	5.74 × 10^4^	1.32 × 10^2^	4.49 × 10^–7^	4.78 × 10^–7^	5.88 × 10^–4^	This work
4	1.63 × 10^5^	2.35 × 10^5^	3.36 × 10^5^	4.87 × 10^–7^	3.68 × 10^–7^	5.55 × 10^–6^	This work
5	1.74 × 10^4^	1.92 × 10^4^	2.19 × 10^2^	2.57 × 10^–6^	1.98 × 10^–6^	3.55 × 10^–4^	This work
6	3.65 × 10^5^	3.26 × 10^4^	1.69 × 10^2^	6.92 × 10^–7^	2.97 × 10^–7^	6.68 × 10^–4^	This work
Coumarin-thiosemicarbazones based sensor	3.48 × 10^4^	3.51 × 10^4^	2.98 × 10^4^	4.34 × 10^–6^	4.30 × 10^–6^	3.95 × 10^–6^	^44^
Acridine based thiosemicarbazones sensor	2.86 × 10^3^	–	–	6.17 × 10^–5^	–	–	^45^

Benesi–Hildebrand equation was used to calculate binding constants;

1 $$\frac{{\varvec{b}}}{\Delta {\varvec{A}}}=\frac{1}{{{\varvec{S}}}_{{\varvec{t}}}{{\varvec{K}}}_{{\varvec{a}}}\Delta{\varvec{\varepsilon}}}\times \frac{1}{\left[{\varvec{L}}\right]}+\frac{1}{{\varvec{S}}{\varvec{t}}\Delta{\varvec{\varepsilon}}}.$$

Δ***A*** = ***A***_substrate + anion_ − ***A***_substrate,_ St = Total concentration of substrate, K_a_ = Association constant or Binding Constant, Δε = ε_substrate+anion_ − ε_substrate_ − ε_anion_.

**Figure 4:**
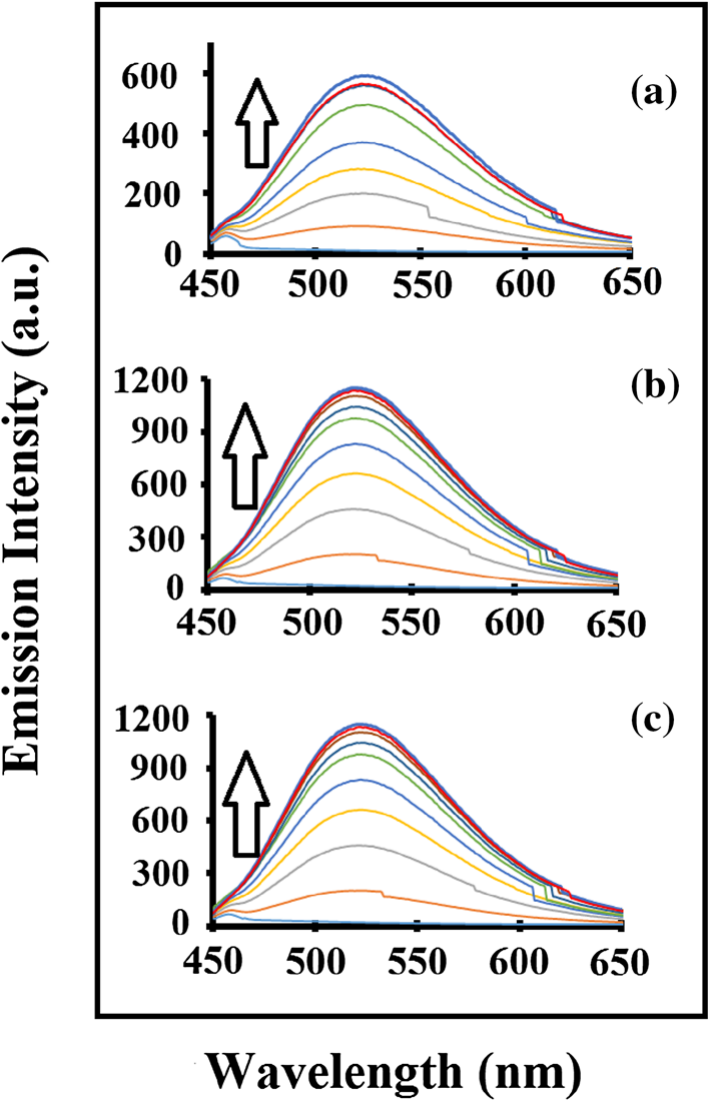
Changes in the fluorescence emission spectra (**a–c**) for compound **4–6** (1 × 10^–4^ mol L^–1^) in acetonitrile respectively upon incremental addition of TBAF (10 equiv) (λ_ex_ = 403 nm).

Dividing the intercept with slope from BH Plot, K_a_ was calculated. Compound **1–3** did not show fluorescence. This could be due release of absorbed energy in a non-radiative process such as transferring excess energy to surrounding molecules, decay to lower but close energy states or reacting with surrounding molecule as has been observed for non-fluorescent compounds^19^. Compound **4, 5,** and** 6** give fluorescence as expected reason being imine derivatives of 4-fluorocinnamaldehyde having fluoro-substituent attached to phenyl rings on both sides of thiourea moiety. They were further investigated for fluorescence studies. It was observed that a new band has appeared at 525 nm when excited at 405 nm. Incremental addition of TBAF (10 equiv) in a fixed volume of compounds **4–6** (1 × 10^–4^ mol L^−1^) was observed to increase the intensity at 525 nm as shown in Fig. 4 below.

### Spectroscopic pattern analysis

**Figure 5:**
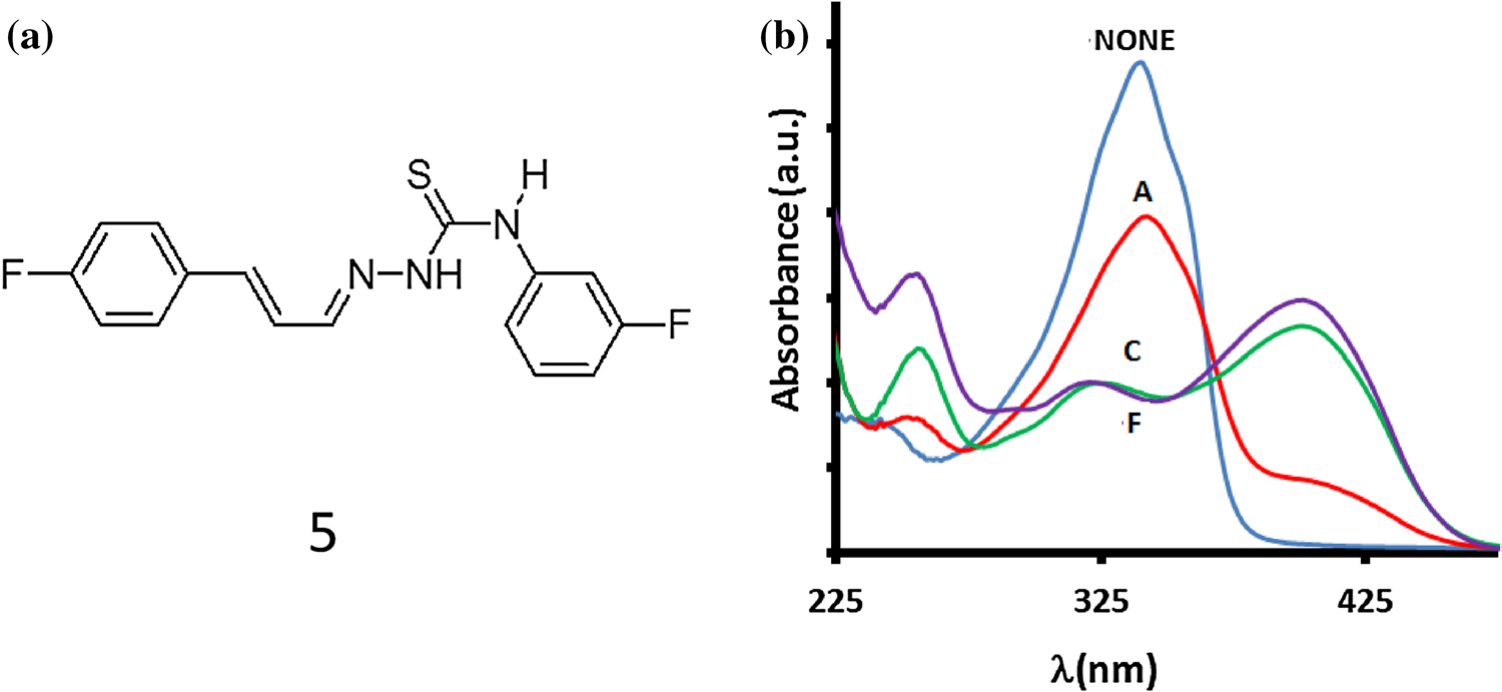
(**a**) Structure of probe **5**. (**b**) Change in spectrum of probe **5** (30 μM) upon the addition of 10 mM acetate (A), cyanide (C), or fluoride (F).

All compound (**1–6**) exhibit capability for sensing for F^−^, AcO^−^, and CN^−^ but derivatives **3** and **5** showed sensing responses to AcO^−^ anions with distinct spectral patterns. Some recent studies have reported fluoro-substituted phenyl giving a curved conformation to the compounds^42^. A curved conformations where a cavity is formed limits the receptor/anions interaction by allowing the anions to approach for the –N–H proton based on the anion size^44^. Among probes **4–6** we selected probe **5** with a fluoro-substituent at meta-position (Fig. 5a) as it has intermediate selectivity for anions but distinct spectral response (Fig. 5b). Hence we anticipated that the fluoro-derivative **5** due to the difference in the interaction of the receptor and anions (AcO^−^/F^−^/CN^−^) could lead us to optimize sequential chemical input and generating distinct spectral responses.

**Figure 6:**
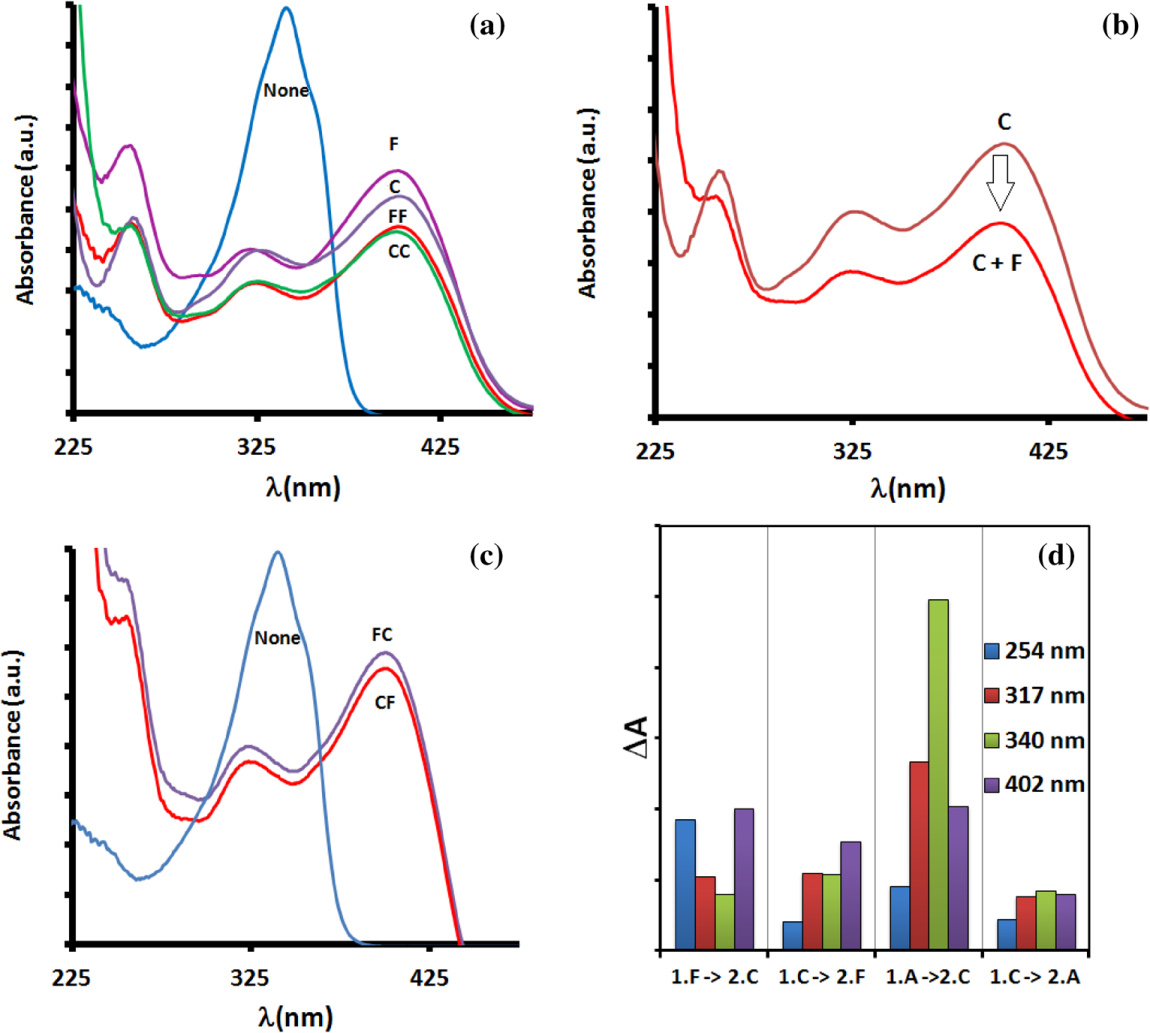
Changes in absorption spectra of **5** (30 µM) upon the addition of (**a**) fluoride (F) and cyanide (C) at different concentration, (**b**) upon changing anion, (**c**) in different orders, (**d**) response of **5** to different sequences of cyanide and fluoride (F) or acetate (A).

We first analyzed the ability of **5** to distinguish between ratios of the same input anions. We noticed that **5** was able to distinguish between the addition of anions (F or C), followed by the second addition of the same anion (FF and CC), indicating that **5** could lead to the generation of a distinguishable absorption pattern (Fig. 6a). According to this, changing the concentration affects the ratio between them. Secondly, we analyzed the ability of **5** to distinguish between different input anions. We used changing the anion i.e. input to CF, which revealed altered interaction of the probe **5** to give changed spectra (Fig. 6b). Upon reversing the sequence the spectra showed minor differences (Fig. 6c). However, probe **5** could recognize AcO^−^ and F^−^ at different spectral patterns. Probe **5** on the addition of AcO^−^ followed by F^−^ could induce 348 nm and 401 nm absorption bands; however, only 401 nm absorption band was observed when reversed the addition sequence. The absorption response at four different wavelengths 254, 317, 340, and 402 nm were recorded to see the change in the spectra between different sequential input systems. The plot in Fig. 6d shows a significant change between the different sequences analyzed. This indicated that the sequential addition of anions favorably interacts with the same sites. This tendency are higher in multivalent receptors which exhibit binding and conformation dynamics which allows the compounds to discriminate among different sequential inputs^46^. Thus we found that our system could produce spectral patterns of distinctive nature and could also distinguish among “passwords” containing different inputs and different ratios of inputs.

**Figure 7:**
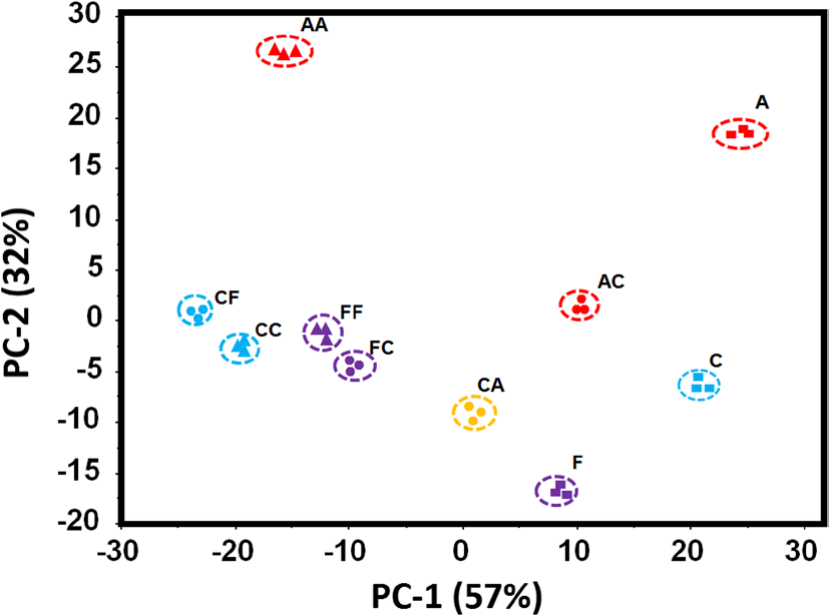
PCA mapping of absorption patterns generated by probe **5** upon the addition of acetate (A) fluoride (F) and cyanide (C) in three replicates of different sequences and concentrations.

The principal component analysis (PCA, Fig. 7) of the complete spectral data shows the probe **5** could discriminate between all possible permutations of anions (F, C, A, FF, CC, FC, CF, etc.) akin to an equivalent 2-digit electronic keypad device. The PCA score plot in Fig. 7 shows the complete segregation of keypad passwords of different combinations. However, the complete separation among the different combinations of passwords are clearly seen along PC-1. The score plot of the PCA model also shows that PC-1 contributes more to separate among sequential combinations as compare to factor-2. PC-1 carries 57% of the spectral variation and PC2, 32% respectively.

**Figure 8:**
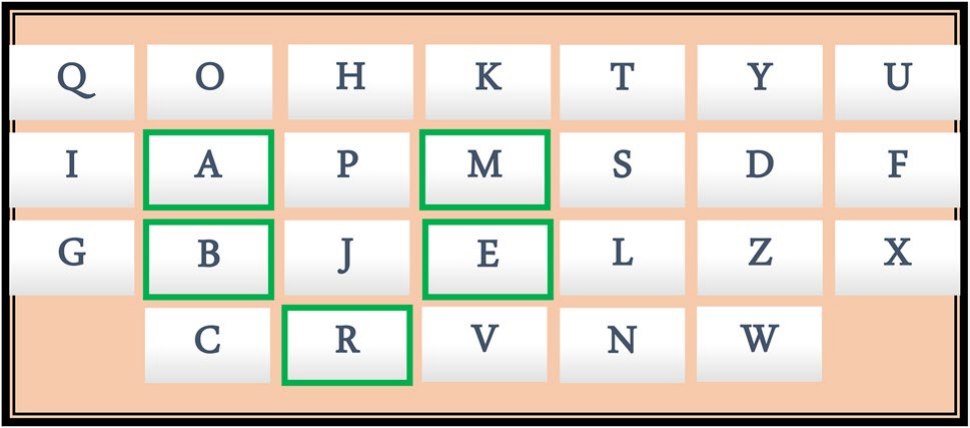
Keypad lock for accessing the secret password generated by sequential chemical inputs of AcO^−^ and F^−^resulting absorption signals at 348 nm and 401 nm respectively.

Since probe **5** shows absorption maxima at 348 nm and 401 nm for AcO^−^ and F^−^ respectively hence sequential chemical input AcO^−^ and F^−^could be utilized as password of the molecular keypad lock for molecular traffic signal with 2-chemical inputs. These observations inspire us to explore probe **5** to develop molecular keypad locks or chemical passwords. To develop a simple molecular keypad lock, chemical input-1 and input-2 were assigned at “A” and “B” while, the outputs signals at 348 nm and 401 nm were assigned at “M” and “E”. Key “R” was assigned for the “OPEN” state of the lock. Now when the input “A” was added first giving 348 nm followed by “B” giving 401 nm absorption band, when detected by the system, this would result in “OPEN” state of the lock. This sequence of chemical inputs would open the lock and the password would be “AMBER” (Fig. 8). All the other sequences would fail to open the lock.

### Combinatorial 3-input password analysis

The changes in the anion (A *vs.* B) and /or its concentration (i.e. A *vs.* AA or B *vs.* BB) could lead to distinct patterns for passwords A, B, AA, and BB. Thus this system could generate unique optical “fingerprints”. Upon addition of OAc^−^, F^−^, and CN^−^ anion, as distinct code entries denoted by A, B, or C a more advanced molecular security system that has the capability of the 3-digit passwords consisting of different permutations of 3-input keys could be developed. Although this possess the challenge of differentiating between the 27 combination as shown in Table 2 below, however, many could be readily be differentiated by the molecular device.

**Table 2:** All possible entry codes of a 3-input keypad lock.

1-Key input	AAA	BBB	CCC
2-Keys input	AAB	BAA	CAA
	ABA	BAB	CAC
	ABB	BBA	CCA
	AAC	BBC	CBB
	ACA	BCB	CBC
	ACC	BCC	CCB
3-Keys input	ABC	BCA	CBA
	ACB	BAC	CAB

**Figure 9:**
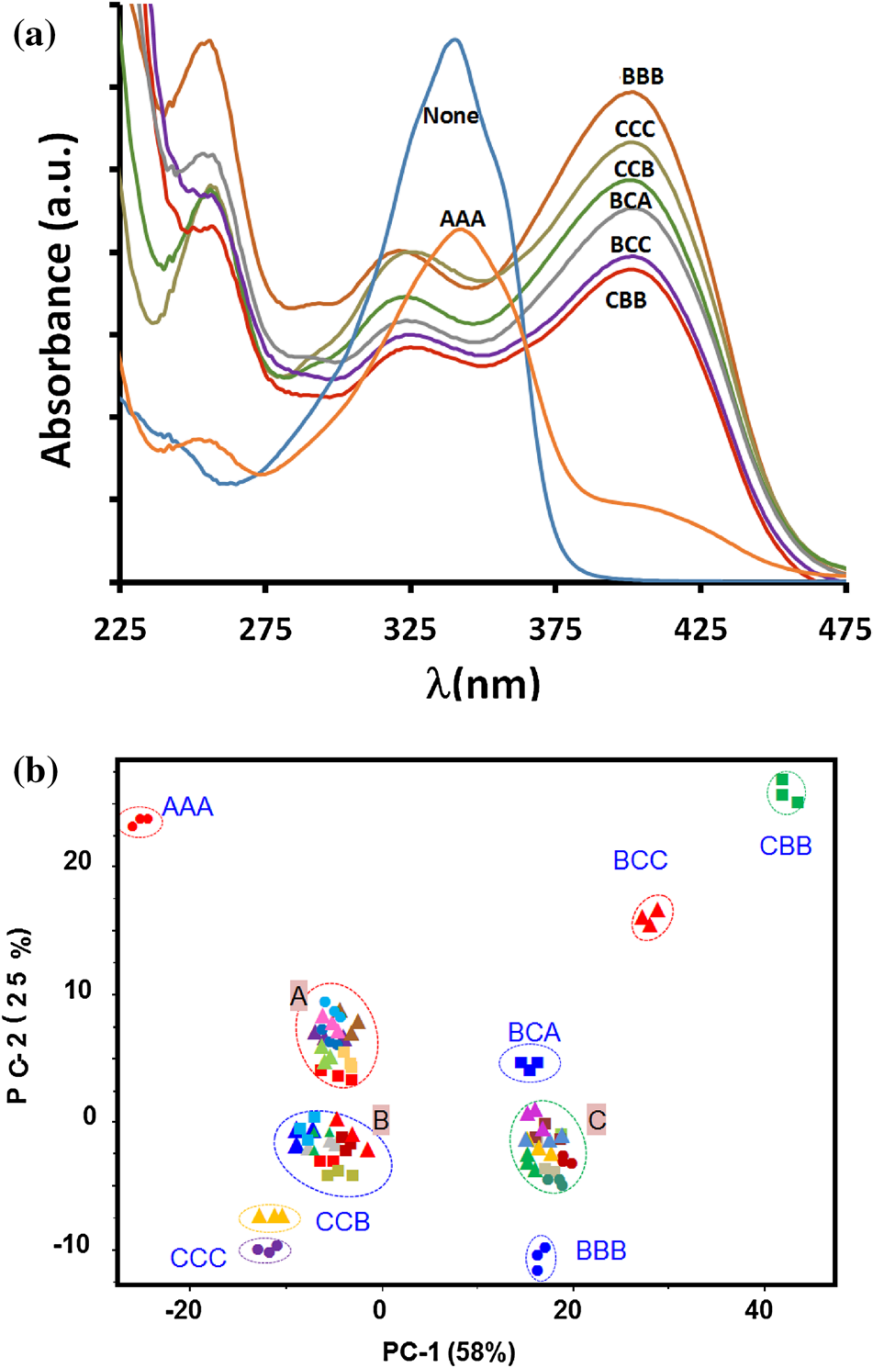
Absorption spectra of **5** (**a**) upon the addition of acetate (A), fluoride (B) and cyanide (C) in different combinations. (**b**) The corresponding PCA plot.

We conducted the sequential additions and the final 3-digit molecular password system was created after testing a wide range of permutation and eliminating any group of sequences that generated overlapping spectra. The non-overlapping patterns generated by different passwords consisting of acetate (A), fluoride (B), and cyanide (C) as input signals are presented in Fig. 9a. The resulting PCA plot was applied to differentiate between the unique chemical passwords (i.e., BBB, CCC, CCB, BCA, BCC, CBB, and AAA) and the remaining combinations that resulted in overlapping patterns i.e. Groups A–C. The pattern analysis of the combinations reveals that seven 3-digit passwords could be authorized by the unimolecular security systems (Fig. 9b). The overlapping spectra formed in three groups. Group A formed from overlapping BBA, CCA, ACC, BAA, AAC, CAA, CAC, ACA while Group B formed from overlapping ABA, BAB, CAB, ABC, ABB, CBA, and group C had BBC, ACB, AAB, BCB, CBC, BAC.

Thus our attempted to explore the ability of this system to process a diverse set of chemical inputs shows that probe **5 **possesses the ability to generate patterns based on the chemical inputs, secondly the pattern generated by the UV–Vis system was efficient in distinguishing among different concentrations of the chemical inputs. This system could authorize multiple users without changing the “lock” or “the key”. Each user could change his password by altering the chemical input. The chemical anions are all colorless and transparent in the visible region and hence their structural and concentration level cannot be determined visually which gives an added security to this system. This work presents an insight to design anion sensors for future information security systems and multifunctional logic devices.

### Theoretical analysis

**Figure 10:**
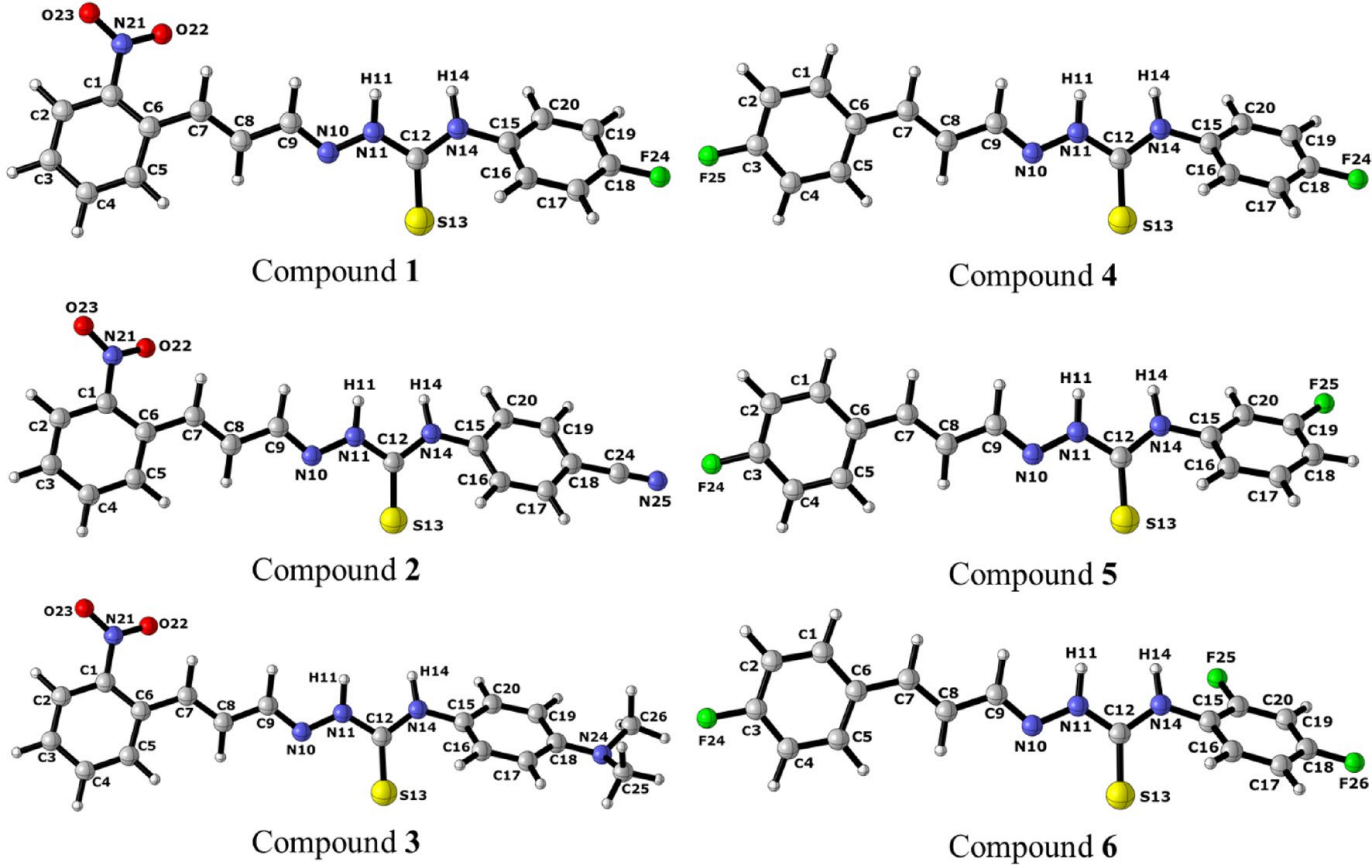
Scheme with the optimized geometries with B3LYP-D3/6–311 + (d,p)/SMD method of compounds **1**–**6**.

The scheme of optimized geometries with B3LYP-D3^47,48^, /6–311 + G(d,p)^49–51^, with water as solvent by applying the implicit solvent model SMD^52^, are shown in the Fig. 10 and Figs. S9 and S10. These calculations were used to obtain the thermodynamic data to calculate the equilibrium constants, pK_a_ and Hammet constants of sensors. These geometries were after utilized to perform the TD-DFT calculations with ωB97X-D3^53^, functional and basis set ma-def2-TZVP^44,54^, with water as solvent by applying the SMD model.

Initially it was investigated the reactivity of the chemosensors **1**–**6** through frontier molecular orbital (FMO) analysis (Table 3). Though the FMO analysis was verified that overall, the chemosensors showed FMO energies remarkably close with an even larger similarity between the chemosensors derived from nitrocinnamaldehyde, particularly **1** and **2** and between derived from fluorocinnamaldehyde, **4**–**6**. The compounds **1** and **2** showed lower HOMO and LUMO values and with lower ΔE_HOMO-LUMO_ energy than **4–6** (fluorocinnamaldehyde derivatives). Compound **3** showed an equivalent LUMO energy to **2**, however with higher HOMO energy than **1** and **2** thus presenting the lowest ΔE_HOMO-LUMO_ between them. This behavior could be explained by the donor nature of the amine group. The compounds derived of fluorocinnamaldehyde (**4**–**6**) showed a behavior remarkably analogous to compound **0** (non-substituted) demonstrating a low influence of –F groups on the FMO of chemosensors in relation to the –NO_2_ group. Although the compound **6** (with two –F substituent groups) demonstrated lowest HOMO and LUMO energies than **4** and **5** and higher ΔE_HOMO-LUMO_.

**Table 3:** Frontier molecular orbitals energies and energy of HOMO → LUMO excitation of compounds **0** and **1**–**6**.

Compound	HOMO (eV)	LUMO (eV)	ΔE (eV)
**0**	− 7.88	− 0.25	7.64
**1**	− 8.12	− 0.93	7.19
**2**	− 8.18	− 0.95	7.23
**3**	− 7.56	− 0.95	6.61
**4**	− 7.87	− 0.20	7.67
**5**	− 7.88	− 0.24	7.64
**6**	− 7.90	− 0.22	7.68

**Figure 11:**
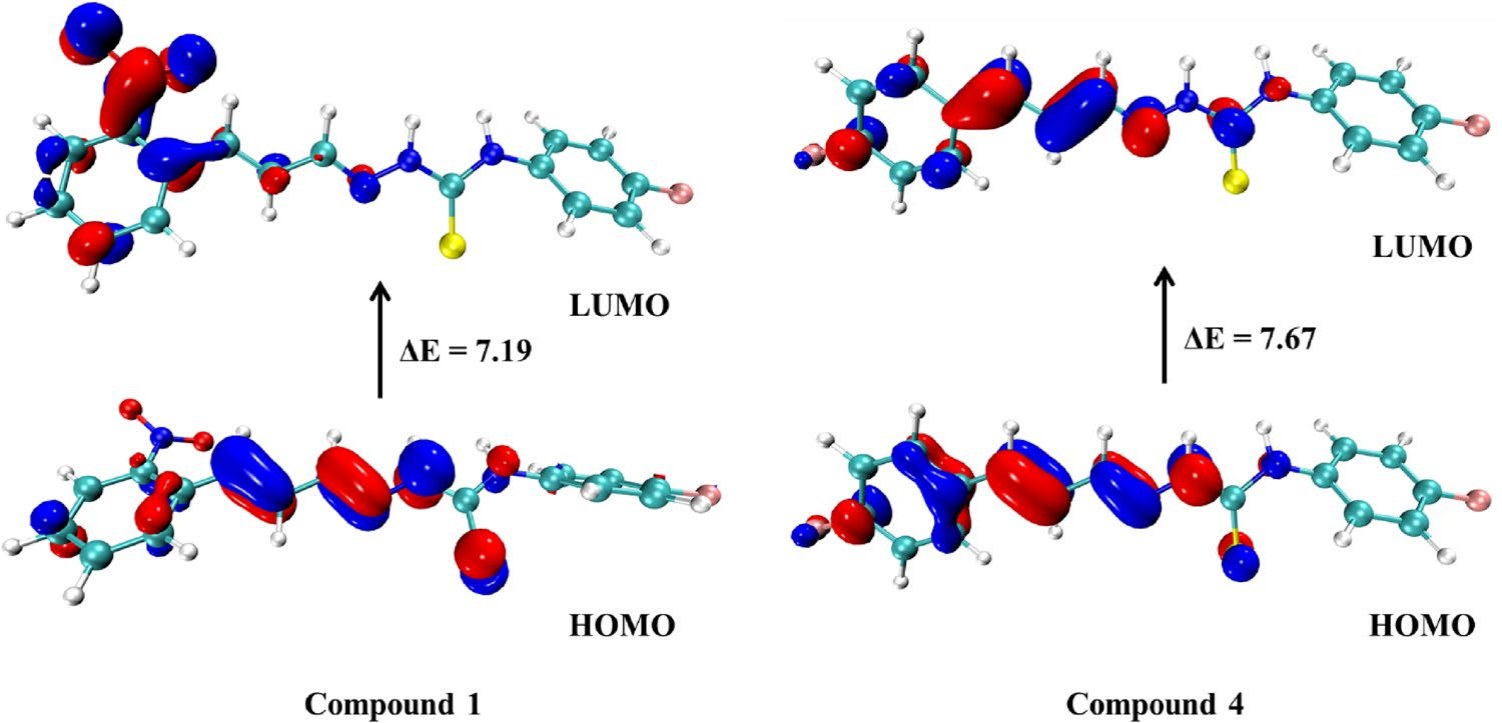
HOMO and LUMO orbitals surfaces of compound **1** (left) and compound **4** (right) with their respective ΔE_HOMO-LUMO_ (eV).

**Figure 12:**
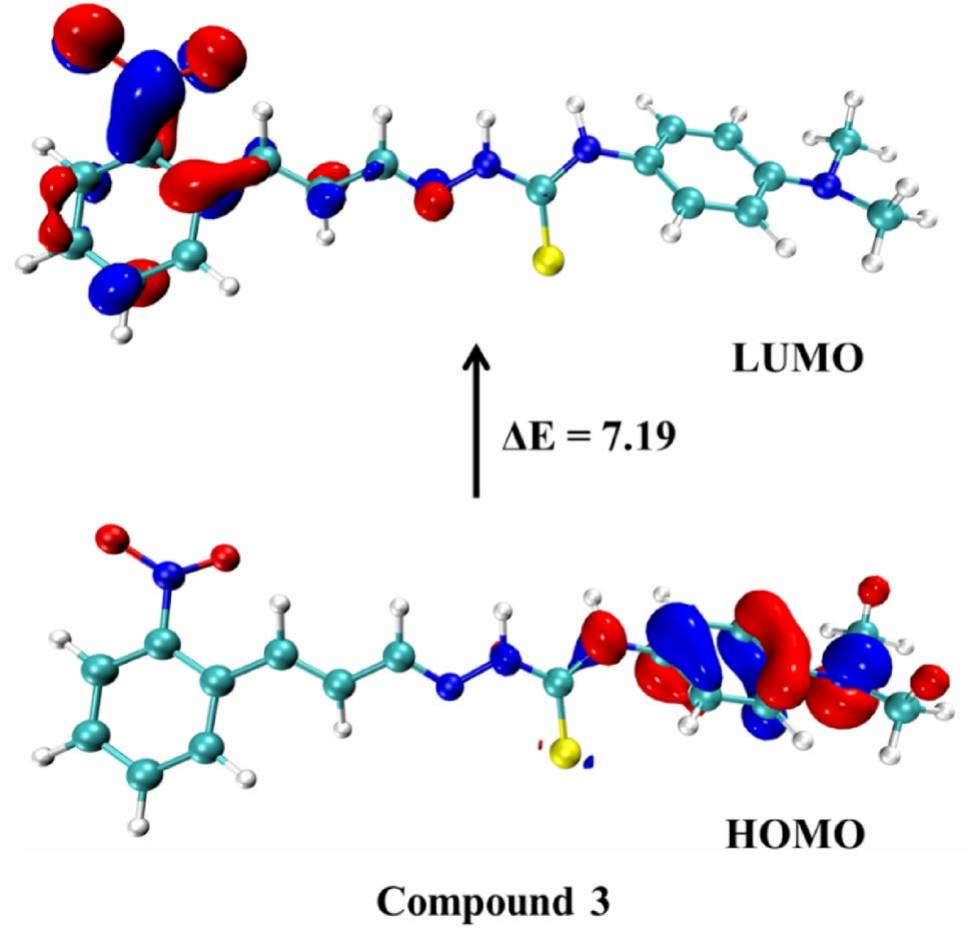
HOMO and LUMO orbitals of compound **3** with its respective ΔE_HOMO-LUMO_ (eV).

The analysis of the surfaces of HOMO and LUMO orbitals of compound **0** and **1**–**6** showed that the HOMO and LUMO orbitals are remarkably similar for all compounds (Table S1), however, the chemosensors derived from nitrocinnamaldehyde (**1**–**3**) have their LUMO surface with a large concentration in the –NO_2_ group while the derived of fluorocinnamaldehyde have low participation of –F in the LUMO orbital. Demonstrating the higher influence of –NO_2_ group on the FMO of chemosensors. Figure 11 demonstrates this behavior. Besides that, it was verified that the HOMO of compound **3** (Fig. 12) was concentrated near of amine group, which explains its slightly higher energy than other chemosensors as discussed before.

The GRD indices of chemosensors are shown in the Table 4 and demonstrated that the compounds **1**–**6** have a significant analogous reactivity which was expected from the FMO analysis. The high ΔE_HOMO-LUMO_ showed by the compounds **1**–**6** demonstrate high stability in which the **4**–**6** indicates slightly higher chemical stability than **1**–**3**.

**Table 4:** Global reactivity descriptors (GRD) of compounds **0** and **1**–**6**.

Compound	IP	EA	χ	μ	η	ω	σ
**0**	7.88	0.25	4.06	− 4.06	3.82	2.16	96.95
**1**	8.12	0.93	4.53	− 4.53	3.59	2.85	103.02
**2**	8.18	0.95	4.56	− 4.56	3.61	2.88	102.47
**3**	7.56	0.95	4.25	− 4.25	3.30	2.74	112.03
**4**	7.87	0.20	4.04	− 4.04	3.83	2.12	96.59
**5**	7.88	0.24	4.06	− 4.06	3.82	2.16	96.90
**6**	7.90	0.22	4.06	− 4.06	3.84	2.15	96.44

The compound **2** present the highest ionization potential (IP) values which indicate higher chemical stability and inertness. Overall, the compounds **1**–**3** showed higher values of electronic affinity (EA) than **4**–**6** which demonstrate that compounds **1**–**3** can easily take electron than **4**–**6**; this behavior should be related to the –NO_2_ group. The sensors **1**–**3** also showed slightly higher values of electronegativity (χ) and higher negative values of chemical potential than **4**–**6**, these two indices associated indicate that compounds **1**–**3** have a higher tendency to attract electrons than **4**–**6**. However, the compounds **4**–**6** showed higher hardness (η) values than **1**–**3**; this behavior should be related to the presence of –F groups. The compounds **1**–**3** also showed higher values of electrophilicity (ω) than **4**–**6**. Thus, the chemosensors **1**–**3** should attract the anions more than **4**–**6**. The sensors **1**–**3** also presented higher values of softness (σ) than **4**–**5**.

To further, investigate the excitations of chemosensors were performed the hole-electron analysis of chemosensors. The first ten excitations (states 1–10) of chemosensors **1–6** were calculated (Tables S2–S8). However, to facilitate our analysis just the two main excitations of chemosensors (**1**–**6**) were further evaluated. Thus, the two excitations with the highest *f*_osc_ were chosen. These excitations are arranged in Table 5 (largest *f*_osc_) and Table 6 (second largest *f*_osc_). Were observed that for chemosensors **1**–**3** (nitrocinnamaldehyde derivatives) the excitations with larger *f*_osc_ are the excitations S_0_ → S_4_ (state 4) and for chemosensors **4**–**6** (fluorocinnamaldehyde derivatives), are the excitation S_0_ → S_2_ (state 2). Overall, the nitrocinnamaldehyde showed lower wavelengths and higher excitation energies (for its equivalent excitations) than fluorocinnamaldehyde derivatives.

**Table 5:** Hole-electron analysis indices for the excitations with highest *f*_osc_ of compounds **0** and **1**–**6**.

Compound	0	1	2	3	4	5	6
Excitation	S_0_ → S_2_	S_0_ → S_4_	S_0_ → S_4_	S_0_ → S_4_	S_0_ → S_2_	S_0_ → S_2_	S_0_ → S_2_
Wavelength (nm)	298.1	279.6	282.1	278.7	296.3	298.0	295.7
*f*_osc_	1.961	0.833	1.007	1.066	1.918	1.950	1.908
E (eV)	4.16	4.43	4.39	4.45	4.18	4.16	4.19
*D* (Å)	0.21	1.17	0.95	1.39	0.11	0.07	0.11
E_Coul_ (eV)	4.75	4.23	4.07	3.89	4.76	4.72	4.76
S_r_	0.79	0.73	0.73	0.74	0.79	0.79	0.79
*H* (Å)	3.76	4.06	4.37	4.70	3.75	3.80	3.74
*t* (Å)	− 3.17	− 2.03	− 2.25	− 2.77	− 2.60	− 2.24	− 2.41
HDI	7.62	7.11	6.81	6.26	7.52	7.57	7.52
EDI	6.99	6.78	6.92	6.78	7.09	6.98	7.10

**Table 6:** Hole-electron analysis indices for the excitations with second highest *f*_osc_ of compounds **0** and **1**–**6**.

Compound	0	1	2	3	4	5	6
Excitation	S_0_ → S_3_	S_0_ → S_1_	S_0_ → S_2_	S_0_ → S_1_	S_0_ → S_3_	S_0_ → S_3_	S_0_ → S_3_
Wavelength (nm)	260.2	279.6	331.8	326.5	256.3	260.5	251.2
*f*_osc_	0.361	0.833	0.894	0.790	0.346	0.369	0.263
E (eV)	4.76	3.73	3.74	3.80	4.84	4.76	4.94
*D*(Å)	1.31	1.65	1.51	1.30	1.34	1.14	1.00
E_Coul_ (eV)	4.66	4.15	4.36	4.30	4.47	4.72	3.93
S_r_	0.67	0.68	0.69	0.69	0.68	0.68	0.73
*H* (Å)	3.27	3.96	3.73	3.84	3.51	3.27	4.49
*t* (Å)	− 1.35	− 1.42	− 1.36	− 1.80	− 1.54	− 1.42	− 2.70
HDI	9.62	6.34	6.72	7.68	9.08	9.86	8.25
EDI	7.37	9.45	9.86	10.42	7.31	7.43	6.21

As for FMO and GRD analysis, the hole-electron analysis showed indices values remarkably similar for compounds **1**–**6**. However, the nitrocinnamaldehyde derivatives (**1**–**3**) presented some slight differences in relation to fluorocinnamaldehyde derivatives (**4**–**6**). Though the analysis of hole and electron distribution were verified that the *D* indices for **1**–**3** present medium values, however, the compounds **4**–**6** presented low *D* indices values which indicate that the S_0_ → S_2_ excitations of **4**–**6** have a lower distance between hole and electron distribution than S_0_ → S_4_ excitations of **1**–**3** compounds, and consequently higher E_Coul_ than **4**–**6**. The Sr index of compounds **4**–**6** was also higher than **1**–**3**. However, the H index of compounds **1**–**3** was higher than **4**–**6**, which indicates that a higher extension of the hole and electron distribution of compounds **1**–**3**. The *t* index was substantially < 0 indicating that the main excitation of compounds **1**–**6** are a local excitation (LE). Finally, the HDI and EDI indices of sensors **1**–**3** showed slightly lower than **4**–**6** indicating a higher spatial extension of hole and electron of **1**–**3**. The hole–electron analysis indices demonstrated that the main excitation of compounds **1**-**6** is a local excitation (LE)of π–π* type, as verified by experimental results.

**Figure 13:**
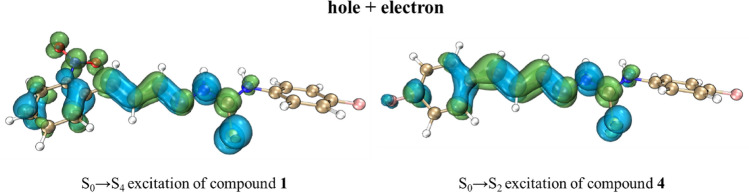
Hole + electron surfaces plot of excitationsS_0_ → S_4_ of compound **1** (left) and S_0_ → S_2_ of compound **4** (right). Hole are the blue surface and electron are the green surface.

The analysis of hole + electron surfaces (Tables S9–S15) demonstrates that the excitation with highest *f*_osc_ along **1**–**6** are again similar. However, it could be observed in Fig. 13 that **1** respective excitation has a higher hole extension and electron spatial distribution than **4** besides a higher separation between hole and electron. This behavior should be related to the presence of –NO_2_ group in the sensors **1**–**3** and demonstrates a higher CT character in **1**–**3** than **4**–**6**.

For the excitations of sensors **1**–**6** with the second highest *f*_osc_, although the values of hole-electron analysis indices are remarkably similar, the trend between **1**–**3** and **4**–**6** were exchanged. The sensors **1**–**3** showed higher wavelength and lower excitation energies than **4**–**6**. For this excitation, the sensors **4**–**6** showed medium values of *D* indices as for **1**–**3** sensors, although also lower than **1**–**3**. The S_r_ index values of this excitation are lower than the excitation with the highest *f*_osc_ which indicates a possibility of *n*–π* excitation type. The t index remained with a value < 0, but with lower values, which indicates an increase of CT in the excitation. The HDI and EDI also are higher than the previous excitation and indicate an increase of CT in the excitation. However, this excitation were still a local excitation.

**Figure 14:**
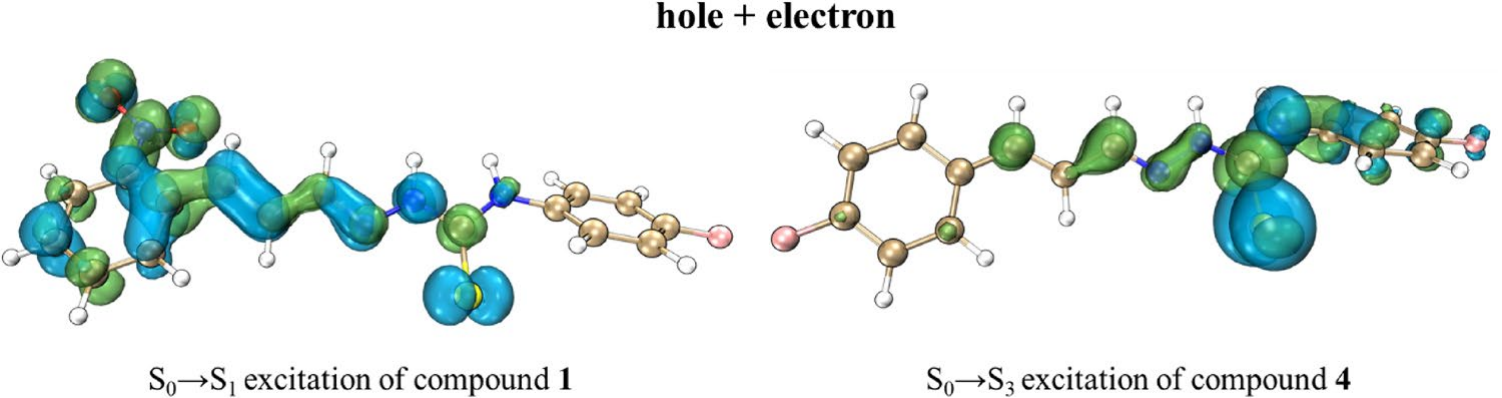
Hole + electron surfaces plot of excitationsS_0_ → S_1_ of compound **1** (left) and S_0_ → S_3_ of compound **4** (right). Hole are the blue surface and electron are the green surface.

It was verified, through the analysis of hole + electron surface (Fig. 14) and the analysis of atomic contributions to the hole, that the excitation S_0_ → S_1_ of compound **1** have a contribution of 11.93% of S atom to the hole and the excitation S_0_ → S_3_ o sensor **4** have a contribution of 50.45% of S atom to the hole (Tables S17, S20, respectively). Therefore, along with the hole–electron indices analysis, the hole + electron surfaces indicate that the excitation S_0_ → S_1_ of compound **1** was an excitation of π–π* type while the S_0_ → S_3_ of compound **4** was an excitation of *n*–π* type which demonstrate the importance of the S atom of thiosemicarbazides to the excitations of the molecule which should be reflected in the UV–vis spectrum of sensors.

Besides that, other important excitation with lower *f*_osc_ beyond low excitation energies are excitation of *n*-π* type with the *n* orbitals of S atom. This excitation was present in all sensors and has in general the lowest excitation energy with an exception for compounds **1** and **3**.

**Figure 15:**
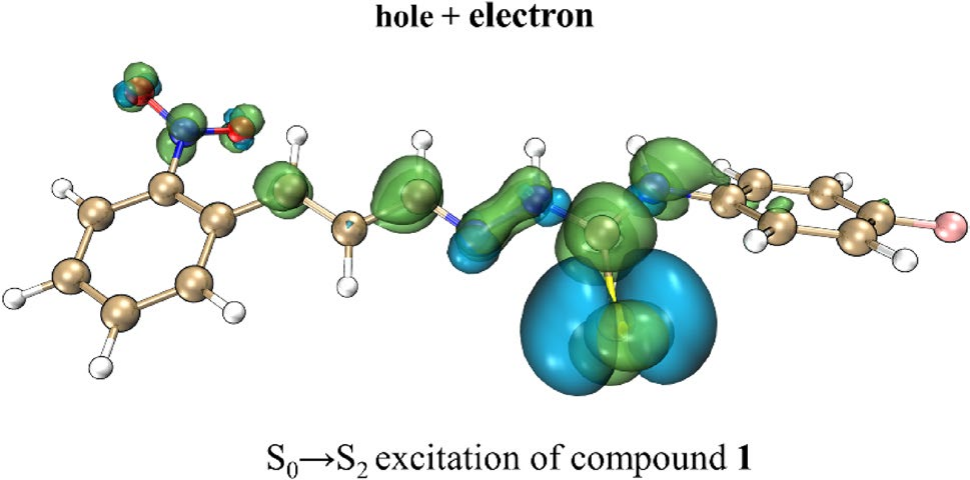
Hole + electron surfaces plot of excitation S_0_ → S_2_ of compound **1**. Hole are the blue surface and electron are the green surface.

The plot of the hole and electron distribution of this excitation were shown in the Fig. 15, for compound **1** this excitation was the excitation S_0_ → S_2_, but for compounds **0** (reference, non-substituted), **2**, **4**, **5** and **6** was the excitation S_0_ → S_1_, and finally for sensor **3** was the excitation S_0_ → S_3_ as could be verified in Tables S9–S15. This excitation demonstrates the importance of the S atom as a donor in the sensors. In the compounds **4**–**6** the influence of the S atom were even more highlighted representing on average 80% of contribution to the hole of the first excitation (Tables S16–S22).

Is also, important highlight, that the hole and electron of compounds **1**–**6** are composed of more than one molecular orbital (MO) with different percentages of contributions to the formation of holes and electrons. In the supplementary material are showed the contributions of each MO with the percentage of contribution above 5% to the hole or electron (Tables S16–S22). The main MO that contributes to the composition of hole and electron of main excitations of compounds 1–6 are shown in Tables S23–S29.

In order to further understand the nucleophilic regions of the sensors were performed a charge analysis of compounds **1**–**6**. Through the analysis was verified that the bond N–H of thiosemicarbazides were the more nucleophilic bonds and the charges of H atoms and atoms that are neighbored to it were shown in Table 7. The GRD analysis indicates that sensors **1**–**3** showed slightly high nucleophilicity and the CHelpG charge distribution of H atoms bonded to N atom from thiosemicarbazides agree with this behavior. The charge analysis demonstrates that H11 and H14 atoms of compounds **1**–**3** were more positively charged than **4**–**6**. Besides that, the H14 atom was more positively charged than H11 for all compounds along with the N14 atom (bonded to H14) was negatively charged against the N11 atom (bonded to H11) was positively charged, which indicates that H14 should be the H atom first dissociated in an acid–base equilibrium. Thus, the H14 atom was considered more acid for the dissociation of the sensor in the acid–base equilibrium to the calculation of acid–base equilibrium and pK_a_ of **1**–**6**. The optimized structures of sensor anions are shown in the Fig. S1 and the geometries for compound 0 and its anion are shown in the Fig. S2.

**Table 7:** CHelpG charges of selected atoms of compounds **0** and **1**–**6**.

Atom	Compound						
	0	1	2	3	4	5	6
N11	+ 0.0393	+ 0.0095	+ 0.0208	+ 0.0230	+ 0.0173	+ 0.0210	− 0.0493
H11	+ 0.2612	+ 0.2749	+ 0.2769	+ 0.2655	+ 0.2666	+ 0.2650	+ 0.2795
N14	− 0.7065	− 0.6527	− 0.7151	− 0.6538	− 0.6508	− 0.6751	− 0.5648
H14	+ 0.4080	+ 0.3980	+ 0.4188	+ 0.3982	+ 0.3975	+ 0.4080	+ 0.3944
C12	+ 0.4773	+ 0.4715	+ 0.4943	+ 0.4699	+ 0.4636	+ 0.4719	+ 0.4833
S13	− 0.5742	− 0.5571	− 0.5390	− 0.5748	− 0.5701	− 0.5604	− 0.5573

To study the acidity of chemosensors and the influence of substituents its $p{K}_{a}$ and theoretical Hammet constants were calculated. To validate our results were performed a brief test of methodology by calculating the $p{K}_{a}$ and ${\sigma }_{X}$ of the benzoic acid and some and substituted benzoic acids (R-bza, R = *m*-NO_2_–, *p*-NO_2_–, *m*-F–, or *p*-F) and compare with experimental results^55^. The results are shown in Tables S30 and S31. It was observed a large error of the calculated ${p{K}_{a}}_{calc}$ and ${{\sigma }_{X}}_{calc}$ with implicit solvent SMD in relation to experimental values, as verified by Thapa and Schlegel^56^. Although, the average error for $p{K}_{a}$ was about 34%, the acidity trends and influence of substituents in the reactivity of the compounds was maintained. Thus we considered this method suitable to the study of chemosensors.

The calculated results for receptors **1**–**6** are shown in the Table 8 and the thermochemical data used to calculate it are shown in Table S32. The analysis demonstrated that in general compounds **1**–**3** was slightly more acids than **4**–**6**, with an exception for compound **6** that showed a second higher acidity followed by **3**. This behavior demonstrates that the push–pull effect in **3** (due to the presence of the donor amine group and the electron-withdrawing substituent –NO_2_) besides the inductive electron-withdrawing effect of two –F substituents in **6**make the receptors more capable to interact with anions as verified by experimental results of association constants. However, the compound **4** showed the lowest acidity along with all receptors, including the reference 0 without substituents, which do not agree with experimental association constants. This behavior demonstrates that other effects should be associated with receptors reactivity beyond the widespread substituent effects.

**Table 8:** Relative Gibbs free energy ($\Delta {G}_{aq}^{*}$, calculate by using the Eq. (10)) in kcal/mol, equilibrium constant (${K}_{a}$, calculate by using the Eq. (14)), $p{K}_{a}$(calculate by using the Eq. (10), and Hammet constant (${\sigma }_{X}$, calculate by using the Eq. (12)).

Compound	$\Delta {G}_{aq}^{*}$	${K}_{a}$	$p{K}_{a}$	${\sigma }_{X}$
0	22.51	3.08 × 10^–17^	16.51	0.00
1	19.99	2.16 × 10^–15^	14.66	1.85
2	17.41	1.69 × 10^–13^	12.77	3.74
3	22.14	5.76 × 10^–17^	16.24	0.27
4	22.92	1.55 × 10^–17^	16.81	− 0.30
5	20.90	4.68 × 10^–16^	15.33	1.18
6	18.71	1.90 × 10^–14^	13.72	2.79

The calculated Hammet constants ${\sigma }_{X}$ reveal that all sensors showed positive value of ${\sigma }_{X}$, which demonstrate that the substituents favored the proton dissociation, except for the compound **4** that showed a ${\sigma }_{X}$ =  − 0.30. It was observed by analyzing the Hammet constants, that the sensors which most favor the proton dissociation are the compounds **3** and **6**, with the highest ${\sigma }_{X}$ values (3.74 and 2.79, respectively), in agreement with experimental constant dissociations.

The equilibrium constants of proton dissociation equilibrium between sensors and anions (F^−^, AcO^−^, Br^−^, Cl^−^, HSO_4_^−^, ClO_4_^−^, CN^−^ and SCN^−^) were also calculated and are shown in the Table S34. It was observed through our analysis that just the anions F^−^, AcO^−^ and CN^−^ presented equilibrium constants above the order of 10^–10^, demonstrating equilibrium with these three anions are favored, in agreement with experimental results. It was observed through our analysis that just the anions F^−^, AcO^−^ and CN^−^ presented equilibrium constants above the order of 10^–10^, demonstrating a equilibrium with this three anions are favored, in agreement with experimental results. Furthermore, the ${K}_{eq}$ for F^−^, AcO^−^ and CN^−^ anions showed the follow trend: CN^−^ > F^−^ > AcO^−^ (Table 9), in agreement with experimental trend for binding constants, except for compounds **3** and **6**. Is important to highlight that, although the experimental trend was retained, the absolute ${K}_{eq}$ values are far from binding constants experimental values because of implicit solvent model limitations for the calculation of solvation energy (ΔG_sol_) for such chemical systems. However, as discussed before, this model was considered suitable for a qualitative evaluation of dissociation equilibrium between sensors and anions.

**Table 9:** Equilibrium constants (${K}_{eq}$) for compounds **1**–**6** proton dissociation equilibrium between sensors and anions (F^−^, CN^−^, and AcO^−^).

Compound	${K}_{eq}$		
	F^−^	CN^−^	AcO^−^
**1**	1.41 × 10^–4^	4.05 × 10^–3^	3.40 × 10^–8^
**2**	1.10 × 10^–2^	3.15 × 10^–1^	2.65 × 10^–6^
**3**	3.76 × 10^–6^	1.08 × 10^–4^	9.06 × 10^–10^
**4**	1.01 × 10^–6^	2.89 × 10^–5^	2.43 × 10^–10^
**5**	3.06 × 10^–5^	8.75 × 10^–4^	7.36 × 10^–9^
**6**	1.24 × 10^–3^	3.56 × 10^–2^	2.99 × 10^–7^

Is expected that anions interact with sensors though H-bonds with thiourea protons before its deprotonation though a excited state proton transfer (ESPT)^57,58^. In order to evaluate this anion-sensor interactions were performed geometry optimization calculations of receptors interacting with anions through H-bonds with thiourea protons. The relative Gibbs free energies and enthalpy energies for the receptor-anion interaction were calculated and their values are shown in the Tables S35–S36. It was verified that the Gibbs free energies (Table S35) presented negative values for receptor-anion interactions for the complexes with F^−^ anion. Meanwile the receptor complexes with CN^−^ and AcO^−^ anions showed endergonic, except for compound **2** that showed slightly negative values of interactions with CN^−^ and AcO^−^ (− 0.03 and − 0.04 kcal/mol, respectively). However, the enthalpy energies (Table S36) showed negative values and demonstrated that the strength of interaction between receptors and anions follows the trend F^−^ > AcO^−^ > CN^−^. This trend is different of the binding energies trend that showed higher binding constants for CN^−^ than AcO^−^, but this behavior is associated to the H-bonds with thiourea protons and neglect the equilibrium and interactions between anions and solvent as discussed before.

**Figure 16:**
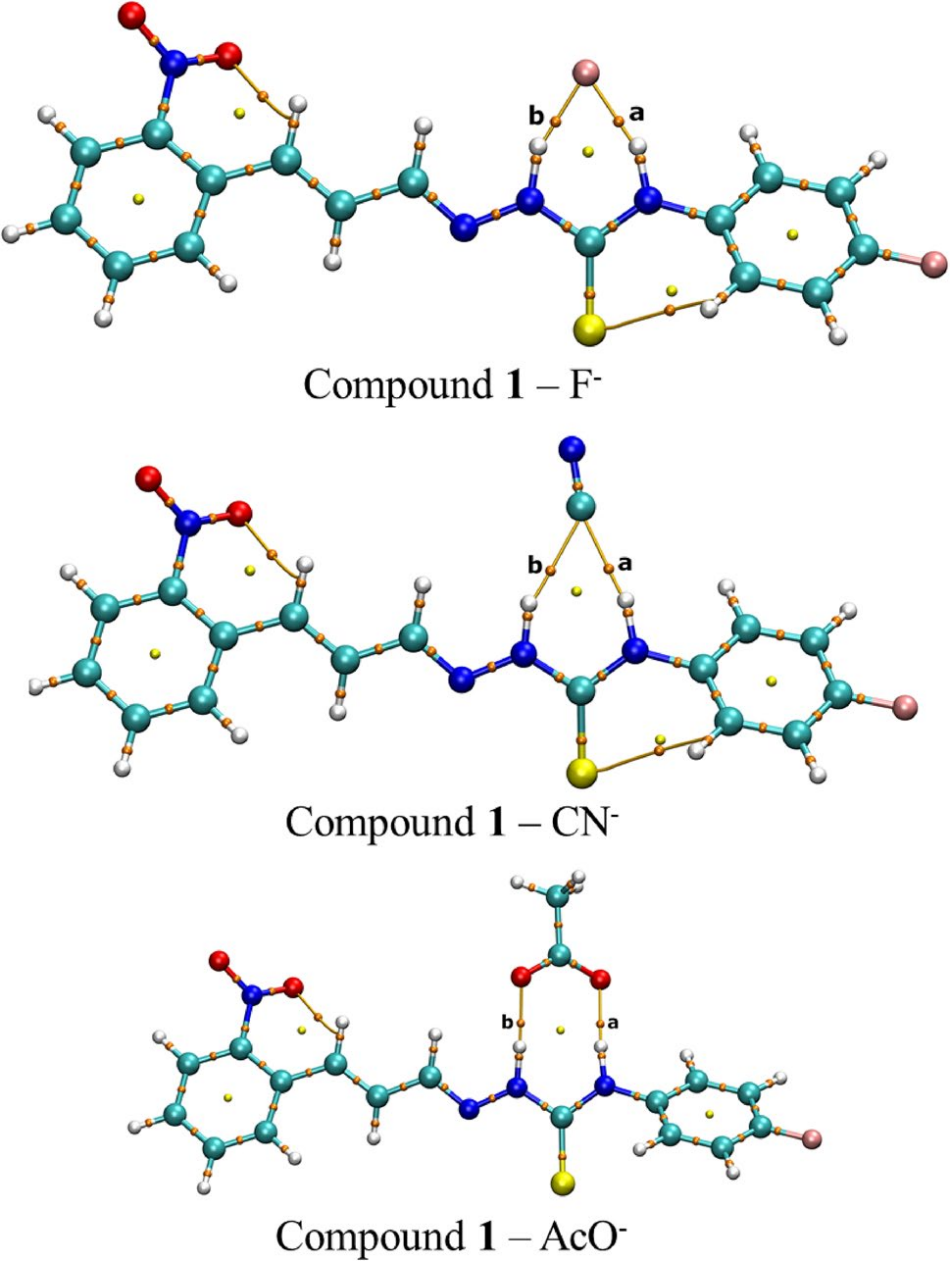
AIM molecular graphs of optimized geometries with B3LYP-D3/6–311 + (d,p)/SMD method of compounds **1**–**6** complexed with anion F^−^. Tiny orange spheres (BCPs), tiny yellow spheres (RCP) and orange lines (BPs). H atoms (white spheres), C atoms (light blue spheres), N atoms (blue spheres), O atoms (red spheres), F atoms (pink spheres), and S atoms (yellow spheres).

To further investigate the H-bonds between receptors and anions F^−^, CN^−^ and AcO^−^ were performed AIM analysis of these complexes. The AIM molecular graphs for the receptor-anion complexes are shown in Fig. 16 (Compound **1** interacting with F^−^, CN^−^ and AcO^−^ anions) and Fig. S11 (compounds **1**–**6** interacting with F^−^ anion), Fig. S12 (compounds **1**–**6** interacting with CN^−^ anion), and Fig. S13 (compounds **1**–**6** interacting with AcO^−^ anion). The AIM properties for BCPs ***a*** and ***b*** with are relate to the H-bonds with thiourea hydrogen are shown in Table S37. It was observed, through the analysis of molecular graphs, the presence of bond paths, BPs (orange lines), between H atoms (of thiosemicarbazide group) and anions which proved the existence of the H-bond between sensor and anions. The bond critical points, BCPs (tiny orange spheres), of the interaction were labeled as ***a*** and ***b***.

The analysis of AIM properties (Table S37) demonstrate that H-bonds (BCP ***a*** and ***b***) are non-covalent interactions due the positive values of Laplacian of the electronic density, ∇^2^ρ(r), a long with electronic density values, ρ(r) < 0.1 a.u. In order to simplify the analysis of H-bonds strength through the AIM properties were performed calculations of binding energy of the H-bonds through the equation presented by Espinosa^59^, that allows to calculate the BE by applying the density of potential energy, *V*(r), at the BCP. The BE values are shown in the Table 10 and demonstrate a similar behavior to the enthalpy values for receptor-anions interaction. It was observed the same trend of H-bond strength (H-bond stronger between sensor and F^−^ followed by AcO^−^ and CN^−^, respectively) than verified trough enthalpy energy analysis.

**Table 10:** Binding energies (BE) calculate through the equation presented by Espinosa (BE = V(r)/2) for compounds **1**–**6** interactins with anions (F^−^, CN^−^, and AcO^−^).

Compound	BCP	BE (kcal mol^−1^)		
		F^−^	CN^−^	AcO^−^
**1**	***a***	− 12.86	− 4.64	− 10.48
	***b***	− 14.53	− 3.01	− 9.88
**2**	***a***	− 14.09	− 5.87	− 14.97
	***b***	− 14.97	− 2.42	− 10.64
**3**	***a***	− 12.02	− 3.33	− 9.63
	***b***	− 14.62	− 4.14	− 9.95
**4**	***a***	− 13.68	− 5.27	− 10.13
	***b***	− 13.05	− 2.35	− 9.91
**5**	***a***	− 13.93	− 5.37	− 10.07
	***b***	− 13.40	− 2.48	− 10.10
**6**	***a***	− 13.99	− 5.05	− 10.45
	***b***	− 13.30	− 2.57	− 10.17

Moreover, other important observation is that compound **4** has higher experimental association constant values than **1**. The theoretical calculations (through GRD, hole-electron and Hammet constant analysis) demonstrate that **1** have more electrophilic character than **4**, due to the electron-withdrawing effect of –NO_2_ group that exerts a withdrawal effect by resonance, which was verified through the hole–electron analyses of excitations with great participation of MO at –NO_2_ group. Although, this electrophilic behaviour for **1**, the AIM analysis demonstrate that the BCP ***a***, related to the H-bond between anion and the hydrogen that should be deprotonated, has higher BE for **4** (BE =  − 13.68 kcal mol^−1^) than **1** (BE =  − 12.86 kcal mol^−1^), that should related with higher experimental association constant values for **4** than **1**.

This trend observed for compounds **1** and **4**, through the theoretical calculations agrees with the experimental^55^, trends presented by Hammett constants(${{\sigma }_{m-N{O}_{2}}}_{exp}$= 0.71 and ${{\sigma }_{p-F}}_{exp}$= 0.06) which demonstrate a higher electron withdrawing of *m*-NO_2_ group (substituent in **1**) than *p*-F (substituent in **4**). Thus, the 4 experimental association constants higher than 1 should be related with other effects instead of widespread *p*-F electron-withdrawing. Overall, the theoretical results indicate that compounds **3** and **6** showed the higher electrophilic character and capacity to interact in the anions which agree with experimental association constants, although all the sensors presented in this work showed a remarkably similar reactivity behavior.

## Conclusion

In Summary, we have developed new types of cinnamaldehyde (2-nitro & 4-fluoro) sensors **1–3** and **4–6,** respectively, for anion sensing based on substituted thiosemicarbazides. These have been characterized by UV–Vis, IR, ^1^H NMR, ^13^C NMR, and mass and theoretical calculations. The theoretical analysis demonstrates that all receptors **1**–**6** showed a similar reactivity, although the GRD beyond the analysis of calculated pK_a_ and Hammet constants showed that compounds **3** and **6** showed higher electrophilicity in agreement with experimental association constants. The analysis of proton dissociation equilibrium between **1**–**6** and anions showed that higher equilibrium constants for equilibrium between **1**–**6** and anions F^−^, CN^−^ and AcO^−^. The electronic structures analysis of substituent factors demonstrates that –NO_2_ substituent showed a high withdrawing effect than –F substituent which reflected in the excitations of compounds and their nucleophilicity, pK_a,_ and Hammet constants. The presence of –NO_2_ demonstrates highlighted influence when the amino group were also a substituent in receptor due to the push–pull effect which increases the electrophilicity of **3**. It was observed that the main excitations in **1**–**6** was π–π* type, however, it was verified that overall *n*–π* excitations showed lower excitation energies with a large participation of S atom to the excitation. Besides that, compounds **4**–**6** showed higher participation of the S atom in the excitations due to the influence of –F substituent. It was observed that all receptors **1–3** could recognize fluoride anion in ACN solvent and receptor **4–6** could recognize AcO- and F- in ACN medium. Further, receptor **5** with AcO^−^ and F^−^ has been used to construct a password for molecular keypad lock with AcO^−^ and F^−^ as inputs. Combinatorial sensing was demonstrated as a powerful technique for password protection at the molecular scale. Eleven 3-digit passwords could be authorized by the uni-molecular security system. This work provides new insight into the design of anions sensors for future multifunctional logic devices.

## Materials and methods

All the reactions were carried out at room temperature. All chemicals and solvents (HPLC grade) were procured from Merck and were used without further purification. Tetra *n*-butyl ammonium salts for anions employed for colorimetric analysis were obtained from Sigma Aldrich and stored in a fridge or desiccators until use. Progress of the reaction was followed by thin layer Chromatography. Shimadzu 840/Shimadzu prestige-21 and Bruker alpha FT-IR were used for the measurement of FT-IR spectra of the samples. ^1^HNMR and ^13^C NMR spectra were recorded using Bruker (Rhenistetten-Forchheim, Germany) AM 300 MHz and 75 MHz spectrometers. MestNova software was used for integration and calculating coupling constants. Deuterated Dimethyl Sulfoxide (DMSO) and Chloroform (Chloroform-d) were used as standards with specific peaks. UV–Visible and fluorescence spectra were recorded using Shimadzu UV-1800 and spectrofluorophotometer Shimadzu RF-6000 respectively. UV absorbance data were recorded in the range from 200 to 800 nm. Absorbance was recorded in quartz cell (1-cm Width).

### Synthesis of chemosensor compounds (1–6)

#### General synthesis

The functionalization of receptors compounds **1–6** were carried out in two steps following our previously reported protocol. Briefly, the key thiosemicarbazide based receptor-ligands were prepared by reflux reaction in ethanol and addition of hydrazine hydrate (18 mmol) drop wise in reaction vial containing isothiocyanate (15 mmol) at 80 °C for 1–2 h. The reaction was followed by TLC until completion. Precipitates were collected by placing the reaction mixture in an ice bath and then filtered and washed repeatedly using cold ethanol. In the next step reflux condensation reaction of 2-nitrocinnamaldehyde (**1–3**) and 4-fluorocinnamaldehyde (**4–6**) with corresponding thiosemicarbazides in a 1:1 stoichiometry, at 80 °C in ethanol in presence of Hydrochloric acid (0.1 mmol) as catalyst afforded the pure products (Scheme 1).

#### General method for the synthesis of receptor compounds (1–6)

The receptor compounds (**1–6**) were prepared by reacting 2-Nitro (3.28 mmol, 0.581 g) or 4-Fluorocinnamaldehyde (3.28 mmol, 0.492 g) with corresponding thiosemicarbazide (3.31 mmol) at 80 °C in ethanol. Hydrochloric acid (0.1 mmol) was used as catalyst. Solid obtained was separated by the process of filtration and then wash with cold ethanol. Product was dried in desiccators and recrystallized in Chloroform.

#### 4-(4-Fluorophenyl)-1-((E)-3-(2-nitrophenyl) allylidene) thiosemicarbazide (1)


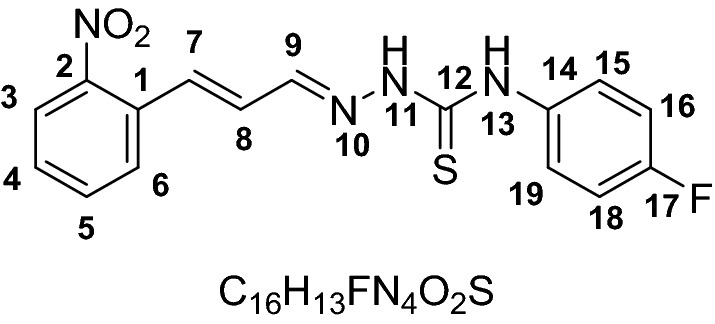


Yield (0.959 g, 85%). M.P 146–148. ESI–MS: m/z 345.077 (*M*^+^); Calculated: 344.363, IR (CH_2_Cl_2_): 1186 cm^−1^ ν(C=S), 1585 cm^−1^ ν(C=N), 3119 & 3318 cm^−1^ ν(N–H). ^1^HNMR (600 MHz, *Chloroform-d*) 11.54 (s, 1H, NH, H-13), 9.13 (s, 1H, NH, H-11), 8.24 (bs, 1H, H3), 7.81 (d, *J* = 7.6 Hz, 2H, H16,18), 7.59 (d, *J* = 7.3 Hz, 1H, H6), 7.48 (bs, 1H, H7), 7.26–7.32 (m, 2H, H4,5), 6.97–7.0 (m, 3H, H15,19, H9), 6.74 (dd, *J* = 15.3 Hz, 9.5 Hz, 1H, H8), ^13^CNMR (151 MHz, *Chloroform-d*) 175.3, 155.4, 153.7, 147.3, 143.8, 133.4, 132.8, 130.8, 129.5, 128.7, 127.7, 126.1, 125.9, 125.7, 125.7, 125.1, 124.3, 123.3, 123.3, 114.8, 114.7.

#### 4-(4-Cyanophenyl)-1-((E)-3-(2-nitrophenyl) allylidene) thiosemicarbazide (2)


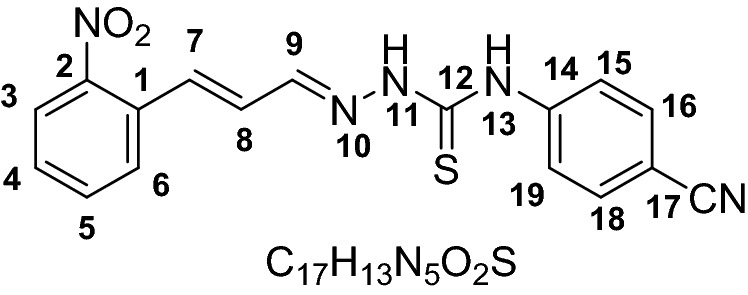


Yield (1.014 g, 88%). M.P 115–153. ESI–MS: m/z 352.0484 (*M*^+^), Calculated: 351.382, IR (CH_2_Cl_2_): 1189 cm^−1^ ν(C=S), 1584 cm^−1^ ν(C = N), 3138 & 3337 cm^−1^ ν(N–H). ^1^HNMR (600 MHz, *Chloroform-d*) 11.67 (s, 1H, NH, H-13), 9.24 (s, 1H, NH, H-11), 7.94 (bs, 1H, H3), 7.72 (dd, 2H, *J* = 7.7 Hz, H16,18), 7.67 (bs, 1H, H6),7.46 (bs, 1H, H7), 7.39 (bs, 1H, H4), 7.23–7.15 (m, 4H, H5, 9, 15, 19), 6.51 (dd, *J* = 13.26, 10.74 Hz, 1H, H8). ^13^CNMR (151 MHz, *Chloroform-d*) 187.3, 175.1, 147.2, 144.2, 138.8, 133.6, 132.9, 130.7, 129.3, 128.9, 128.8, 128.1, 127.7, 127.6, 126.4, 124.4, 117.9, 111.6, 77.3, 77.1, 76.8, 39.9, 39.8, 39.6, 39.5, 39.4, 39.2, 39.1.

#### 4-(4-(Dimethylamino)phenyl)-1-((E)-3-(2-nitrophenyl) allylidene) thiosemicarbazide (3)


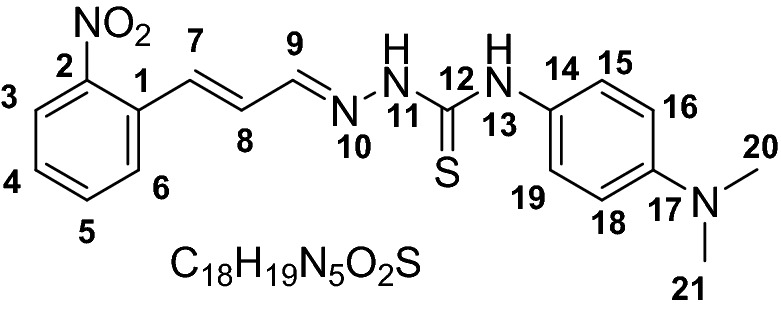


Yield (0. 958 g, 79%). M.P 165–167. ESI–MS: m/z 370.1462 (*M*^+^), Calculated: 369.440. IR (CH_2_Cl_2_): 1198 cm^−1^ ν(C=S), 1590 cm^−1^ ν(C=N), 3132 & 3334 cm^−1^ ν(N–H). ^1^HNMR (600 MHz, *Chloroform-d*) 10.03 (s, 1H, NH, H-13), 8.96 (s, 1H, NH, H-11), 8.02 (d, *J* = 8.0 Hz, 1H, H3), 7.77 (d, *J* = 9.1 Hz, 1H, H6), 7.72 (d, *J* = 7.7 Hz, 1H, H7), 7.64 (t, *J* = 7.5 Hz, 1H, H5), 7.50–7.46 (m, 2H, H4, 9), 7.44–7.41 (m, 2H, H15, 19), 6.88 (dd, *J* = 15.7 Hz, 9.2, 1H, H8), 6.77 (d, *J* = 8.3 Hz, 2H, H16, 18), 2.99 (s, 6H, H-20, 21). ^13^CNMR (151 MHz, *Chloroform-d*) 176.4, 149.3, 147.9, 143.0, 134.3, 133.4, 131.5, 129.4, 128.3, 126.4, 125.2, 112.5, 77.37, 77.2, 76.9, 40.8.

#### (E)-4-(4-fluorophenyl)-1-((E)-3-(4-fluorophenyl) allylidene) thiosemicarbazide (4)


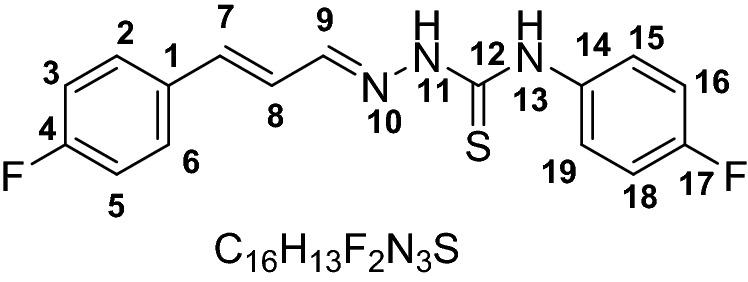


Yield (0. 794 g, 76%). M.P 148–149. ESI–MS: m/z 318.0918 (*M*^+^), Calculated: 317.356. IR (CH_2_Cl_2_): 1188 cm^−1^ ν(C=S), 1586 cm^−1^ ν(C=N), 3219 & 3328 cm^−1^ ν(N–H). ^1^HNMR (600 MHz, *Chloroform-d*) 10.88 (s, 1H, NH, H-13), 9.19 (s, 1H, NH, H-11), 8.37 (bs, 1H, H19), 7.78 (d, *J* = 9.2 Hz, 1H, H9), 7.40–7.38 (m, 2H, H16, 18), 7.09–7.06 (m, 3H, H3, 5, 15), 6.99 (t, 2H, *J* = 8.3 Hz, H2, 6), 6.86 (d, 1H, *J* = 16.0 Hz, H7), 6.74 (dd, 1H, *J* = 15.9 Hz, 9.3 Hz, 8H). ^13^CNMR (151 MHz, *Chloroform-d*) 175.5, 164.0, 162.3, 155.8, 154.2, 145.2, 139.2, 132.2, 128.8, 128.8, 126.5, 126.2, 126.2, 125.5, 124.5, 123.8, 116.0, 115.9, 115.3, 115.2.

#### (E)-4-(3-fluorophenyl)-1-((E)-3-(4-fluorophenyl)allylidene) thiosemicarbazide (5)


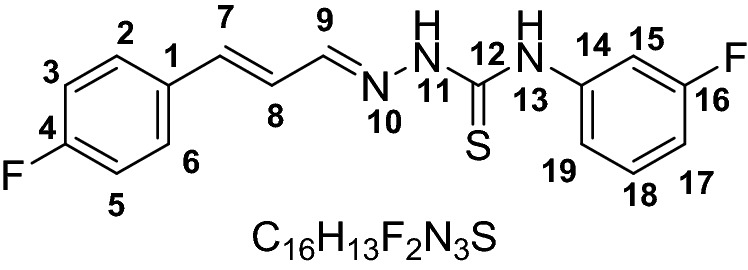


Yield (0.835 g, 80%). M.P 156–157. ESI–MS: m/z 318.0900 (*M*^+^), Calculated: 317.356. IR (CH_2_Cl_2_): 1189 cm^−1^ ν(C=S), 1580 cm^−1^ ν(C=N), 3212 & 3337 cm^−1^ ν(N–H). ^1^HNMR (600 MHz, *Chloroform-d*) 9.89 (s, 1H, NH, H-13), 9.16 (s, 1H, NH, H-11), 7.67 (t, 2H, *J* = 9.24 Hz, H3,5), 7.42–7.44 (m, 2H, H15,17), 7.35 (d, 1H, *J* = 8.0 Hz, H7), 7.30 (dd, *J* = 15.0 Hz, 1H), 7.05 (t, 2H, *J* = 8.3 Hz, H2,6), 6.87–6.92 (m, 2H, H15, 19), 6.77 (dd, *J* = 16.0 Hz, 9.2 Hz, H8). ^13^CNMR (151 MHz, *Chloroform-d*) 175.4, 164.3, 163.6, 162.6, 161.9, 144.7, 139.8, 139.6, 132.1, 129.9, 129.8, 129.0, 128.9, 123.9, 119.1, 119.1, 116.3, 116.1, 112.7, 112.6, 111.1, 110.9, 77.4, 77.2, 76.9.

#### (E)-4-(2,4-difluorophenyl)-1-((E)-3-(4-fluorophenyl) allylidene) thiosemicarbazide (6)


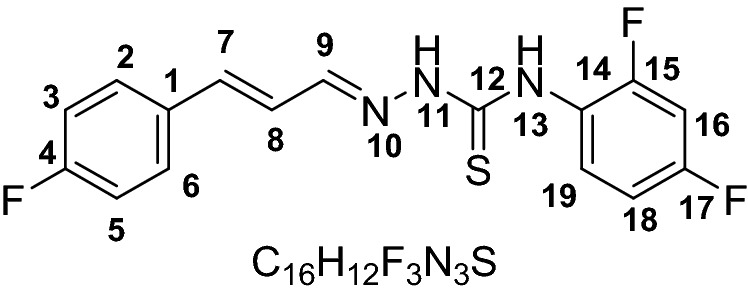


Yield (0. 970 g, 88%). M.P 138–140. ESI–MS: m/z 336.0817 (*M*^+^), Calculated: 335.346. IR (CH_2_Cl_2_): 1188 cm^−1^ ν(C=S), 1585 cm^−1^ ν(C=N), 3125 & 3334 cm^−1^ ν(N–H). ^1^HNMR (600 MHz, *Chloroform-d*) 10.33 (s, 1H, NH, H-13), 9.01 (s, 1H, NH, H-11), 8.17 (m, 1H, H7), 7.74 (d, 1H, *J* = 9.2 Hz, H9), 7.42–7.44 (m, 2H, H3,5), 7.04 (t, 2H, *J* = 8.4 Hz, H, 18,19), 6.92–6.86 (m, 3H, H2,6,16), 6.76 (dd, *J* = 16.02 Hz, 9.21 Hz, H8). ^13^CNMR (151 MHz, *Chloroform-d*) 176.1, 164.3, 162.6, 161.3, 161.2, 159.6, 159.5, 156.5, 156.5, 154.9, 154.8, 145.2, 139.9, 132.1, 132.0, 129.0, 128.9, 127.6, 127.6, 124.0, 122.6, 116.2, 116.1, 111.1, 110.9, 104.3, 104.1, 103.9, 77.4, 77.2, 76.9.

### Spectrophotometric experiments (UV/visible and florescence studies)

UV–visible absorption titrations were performed in water at room temperature. Freshly prepared solutions of sensor **1–6** (5 × 10^−5^ mol L^−1^) were used. Aliquots of aqueous stock solutions of anions were added fluoride (F^−^), chloride (Cl^−^), bromide (Br^−^), iodide (I^−^), perchlorate (ClO_4_^−^), acetate (CH_3_COO^−^), bisulphate (HSO_4_^−^), cyanide (CN^−^)-, and thiocyanate (SCN^−^) diluted with distilled water. The solutions were prepared with 5 × 10^−5^ mol L^−1^ of sensor in distilled water with varying volumes of anions. For Job’s plot equimolar (1 × 10^−4^ M) solutions of Tetra-*n*-butylammonium fluoride (TBAF) and receptors were prepared.

The absorption data were fitted to the equation

$$-\mathrm{log}\left[{A}^{-}\right]=\mathrm{ log}{\beta }_{\mathrm{A}-} +\mathrm{ log}\left[\frac{{A}_{\mathrm{max}}-A}{A-{A}_{\mathrm{min}}}\right],$$

where log [A^−^] is the logarithm molar anion concentration at that point, log *β*_A−_ is the dissociation constant, *A*_max_ is the maximum absorbance at the selected wavelength, *A*_min_ is the minimum absorbance at the given wavelength and *A* is the observed absorbance at that specific wavelength.

Plotting log [(*A*_max_ − *A*)/(*A* − *A*_min_)] *versus* the − log [A^−^], the log *β*_A−_ was derived from the slope of the resulting plot. Titrations were repeated at least twice until the results were reproducible.

Fresh stock solutions were prepared for ligands of strength (5 × 10^–5^ mol L^−1^) and anions of strength (5 × 10^–4^ mol L^−1^). All compound 1–6 were screened for chemo sensing properties. UV scanning was done between 200 and 800 nm upon addition of 30 equiv. of anion solution in ligands solution. Variation in absorption in a fixed volume of ligands 3 mL (3 × 10^–5^ mol L^−1^) was plotted against incremental addition of fluoride (1 × 10^–2^ mol L^−1^) Association constants was calculated using Benesi–Hildebrand Eq. (1);

Florescence studies were carried out using emission spectrum of (**4–6**) molecules having fluorophore nuclei on both sides of the thiourea moiety.

### UV–visible and fluorescence titration

Freshly prepared solutions of probes 1–6 (5 × 10^−5^ mol L^−1^) in acetonitrile were used. Similarly the stock solution of (1 × 10^−4^ mol L^−1^) of different anions like fluoride (F^−^), chloride (Cl^−^), bromide (Br^−^), iodide (I^−^), perchlorate (ClO_4_^−^ ), acetate (AcO^−^), bisulfate (HSO_4_^−^), cyanide (CN^−^), and thiocyanate (SCN^−^)from tetra-*n*-butylammonium salts were also prepared in acetonitrile. The sensing and recognition abilities were evaluated by measuring absorption and emission during the titration with anions.

### Measurements and pattern analysis

#### 2-Input chemical passwords

The dependence of the absorption pattern on the sequence of addition was demonstrated by adding 10 equivalents of two different anion to acetonitrile solution of **5** (30 µM, 3 mL) in different orders. The mixture was allowed to equilibrate for about 1 min after each anion addition. Solutions of acetate anion (10 mM) and fluoride (10 mM); Fluoride (10 mM) and Cyanide (10 mM), were used for obtaining the various 2-input chemical passwords. Absorption experiments were performed in three replicates and principal component analysis (PCA) was applied to distinguish between the absorption patterns generated.

#### 3-Input chemical passwords

As a first step toward developing 3-input chemical passwords, solutions of various Anion were prepared (75 µL, 10 mM) and each solution was added separately to a solution of **5** (30 µM, 3 mL) in Acetonitrile. Following a 1 min equilibration absorbance spectra were recorded in three replicates and PCA was applied to identify chemical inputs that could potentially be used for obtaining 3-digit chemical passwords, namely, the anions that induced the most distinguishable changes to the absorption patterns.

### Principle component analysis

PCA is a non-supervised multivariate exploratory data analysis tool used to reveal the hidden structure within large data sets, to reduce the dimensionality of a complex data set without much loss of information, to extract the most important information from the data, to identify noise and outlier in the data set and to visualize the pattern of grouping based on similarities and dis-similarities in the data. It is also known as a projection method, because it takes information carried by the original variables and projects them onto a smaller number of latent variables called Principal Components (PC). Each PC explains a certain amount of the total information contained in the original data and the first PC contains the greatest source of variation in the data set.

The procedure of PCA is based on to convert a set of correlated variables into a new set of uncorrelated variables called principal components. PCA redistributes the total variance of the data set in such a way that the first principal component has maximum variance, followed by second component and so on. The covariance of any of the principal component with any other principal component is zero (uncorrelated) and they are orthogonal to each other. By plotting PCs important sample and variable interrelationships could be revealed, leading to the interpretation of certain sample groupings, similarities or differences.

In this study PCA was applied to classify sensing behavior of a molecular level keypad lock stimulated by two sequential chemical inputs (AcO- and F-), which has potential for application in security devices. In order to build the PCA models UV–Vis absorption spectral data were used data to build the PCA model and model was internally validated, using a leave one out full cross validation procedure using Singular Value Decomposition (SVD) algorithm with 67 segments and total of 7 components.

### Computational details

All the compounds **1**–**6** were optimized with Gaussian 09^60^, package by applying the DFT method, with B3LYP-D3^47,48^, functional and 6–311 + G(d,p)^49–51^, basis set with water as solvent by applying the implicit continuum solvent model SMD^52^, B3LYP-D3/6–311 + G(d,p)/SMD. Were also performed the geometrical optimization of a non-substituted thiosemicarbazide derivatives (compound **0**) to be used as reference.

In order to explore the non-covalent interactions between receptors and anions it was performed AIM (Atom in Molecules) analysis^61–63^, by applying the Multiwfn package^64^. The AIM data was calculated with B3LYP-D3/6–311 + G(d,p) method with solvent model SMD (water). In order to obtain a more accurate analysis of the excitations and frontier molecular orbitals (FMO) were performed TD-DFT calculations from the optimized structures of **0** and **1**–**6** with ωB97X-D3^53^, functional and ma-def2-TZVP^44,54^, basis set with continuum solvent SMD (water) in Orca package (version 4.2.1)^65,66^, were performed and analysis of the global reactivity descriptors (GRD) which were obtained by applying the equations shown below. The GDR analysis is based in FMO that is in the highest occupied molecular orbital (HOMO) and lowest unoccupied molecular orbital (LUMO) energies (in *eV*) of compounds **0** and **1**–**6**.

The ionization potential (IP) and electron affinity (EA) were given by the Eqs. (2) and (3), respectively^67^.

2 $$IP=-{\mathrm{E}}_{\mathrm{HOMO}},$$

3 $$EA=-{\mathrm{E}}_{\mathrm{LUMO}}.$$

The Koopman’s theorem was used to calculate the global hardness (η)^68^, the electronegativity (χ)^69^, and the chemical potential (μ)^70^, by applying the equations below^71^:

4 $$\chi =\frac{\left[IP+EA\right]}{2},$$

5 $$\eta =\frac{\left[IP-EA\right]}{2},$$

6 $$\mu =\frac{{\mathrm{E}}_{\mathrm{HOMO}}{+\mathrm{E}}_{\mathrm{LUMO}}}{2}.$$

The electrophilicity index (ω)^72^, was calculated through the Eq. (7) reported by Parr et al.:

7 $$\omega =\frac{{\mu }^{2}}{2\eta }.$$

The global softness (σ)^73^, was calculate by the Eq. (8).

8 $$\sigma =\frac{1}{2\eta }.$$

To study the excitations in the chemosensors was applied the hole–electron theory^74–76^. In this theory the electron leaves the hole and goes to the electron in an excitation. For an excitation that is perfect described as a HOMO → LUMO transition, the HOMO could be described as the hole and LUMO as the electron. However, as for Natural Transition Orbitals (NTOs), commonly the hole and electron are composed by multiple molecular orbitals (MO) contributions with different participation percentages to its representations.

The hole and electron distribution could be quantitative characterized through indexes that measure the extension of hole and electron distributions and the separation between them which could be used to evaluate the type of excitation (charge transference, CT, or local excitation, LE). The hole delocalization index (HDI) and electron delocalization index (EDI) measures the breadth of spatial distribution of hole and electron. The HDI and EDI indices showed larger values for concentrate spatial distributions of hole and electron and lesser HDI and EDI indices for delocalized spatial distributions. In a local excitation (LE) the spatial distribution of hole and electron are close. The charge-transfer excitation (CT) has a separation between the hole and electron spatial distribution. The S_r_ index is a measure of the extension of the overlap between hole and electron distribution. A large S_r_ index indicates an excitation of type π–π* while lower S_r_ index indicates a n–π* type of excitation. The *D* index is the magnitude of charge transference (CT) length that is a measure of the distance between centroid of hole and electron, thus high values of D index reveals a CT excitation. The *H* index is a measure of spatial extension of hole and electro distribution. The t index measures the separation degree between hole and electron in CT direction. A *t* index < 0 indicates a small separation between hole and electron due to CT while *t* index > 0 reveals a substantially separation between hole and electron distribution that indicate a CT excitation. Finally, the Coulomb attraction energy (*E*_coul_, also called excitation binding energy between hole and electron) is also related with the excitation type along with *D* index in which higher *D* index leads to lower *E*_coul_ values.

To evaluate the de acidity through $p{K}_{a}$ values and the influence of –R substituent groups in thiosemicarbazide derivatives were calculate the Hammet constants^77^, in terms of calculated $p{K}_{a}$ values, beyond that these results were compared with calculated aromatic indices. All this indices were calculated from thermochemical data obtained through the geometrical optimizations with B3LYP/6–311 + G(d,p)/SMD method.

The $p{K}_{a}$ values were calculated using as reference the work ofThapa and Schlegel^56^, by applying the Eq. (9) below:

9 $$p{K}_{a}=\frac{\Delta {G}_{aq}^{*}}{2.303RT}.$$

The compounds **0** and **1**–**6** was considered as a protonated sensor ($SH$) in a proton dissociation equilibrium in water (simulated through continuum solvent SMD), thus the equilibrium can be written as $SH \leftrightharpoons {S}^{-}+{H}^{+}$. Therefore, the by applying the Eq. (10):

10 $$\Delta {G}_{aq}^{*} = {G}_{aq}^{*}{(S}^{-})+{G}_{aq}^{*}\left({H}^{+}\right)-{G}_{aq}^{*}\left(SH\right).$$

In which, ${G}_{aq}^{*}{(S}^{-})$ and ${G}_{aq}^{*}\left(SH\right)$ are the Gibbs free energy of deprotonated and protonated, respectively, chemosensor in water (SMD) and ${G}_{aq}^{*}\left({H}^{+}\right)$ were calculated as demonstrated in the Thapa and Schlegel^56^, work and presented a value of − 270.296808 kcal/mol. The theoretical Hammet constants^77^, were calculated from an adaptation of the Eq. (11) presented in the work of Hansch et al.^78^.

11 $${\sigma }_{X}=\mathrm{log}{K}_{X}-\mathrm{log}{K}_{H}.$$

Thus, adapting the Eq. (4) we can write the Hammet constant as the Eq. (12):

12 $${\sigma }_{X}={p{K}_{a}}_{H}-{p{K}_{a}}_{X}.$$

In our approach in the adapted Hammet Eq. (5), the ${p{K}_{a}}_{H}$ is the $p{K}_{a}$ of non-substituted thiosemicarbazide derivative (compound **0**) and the ${p{K}_{a}}_{X}$ is the $p{K}_{a}$ of chemosensors **1**–**6**.

To evaluate the chemosensor acid–base equilibrium between sensors ($SH$) and anions (${A}^{-}$), given by $SH+{A}^{-}\leftrightharpoons {S}^{-}+AH$, were calculated its equilibrium constants through the Eq. (13):

13 $${\Delta G}_{eq} = {[G}_{aq}^{*}{(S}^{-})+{G}_{aq}^{*}\left(AH\right)]-\left[{G}_{aq}^{*}\left(SH\right)+{G}_{aq}^{*}\left({A}^{-}\right)\right] .$$

The equilibrium constant was calculated through the Eq. (14):

14 $${K}_{eq}={e}^{\left(\frac{-{\Delta G}_{eq}}{RT}\right).}$$

The electron excitation analysis, FMO, hole-electron analysis and the AIM analysis were performed by applying the Multiwfn program^64^, (version 3.8) and the visualization of analysis was performed with VMD software^79^, and the visualization of optimized structures were performed with CYLview software^80^.

## Supplementary Information

Supplementary Information.
