# Supplementary material for: 2-Nitro- and 4-fluorocinnamaldehyde based receptors as naked-eye chemosensors to potential molecular keypad lock
Source: Sci Rep. 2021 Oct 21;11:20847. doi: 10.1038/s41598-021-99599-w (PMC8531455; doi:10.1038/s41598-021-99599-w)
Supplement: Supplementary file 1 — Supplementary Information. [file 41598_2021_99599_MOESM1_ESM.docx]

Supporting Information

2-Nitro- and 4-Fluorocinnamaldehyde Based Receptors as Naked-Eye Chemosensors to Potential Molecular Keypad Lock

Muhammad Islam,^1^ Zahid Shafiq,*^1^ Fazal Mabood,^2^ Hakikulla H Shah,^2^ Vandita Singh,^3^ Muhammad Khalid,*^4^ Sara Figueirêdo de Alcântara Morais,^5^ Ataualpa Albert Carmo Braga,^5^ Muhammad Usman Khan,^6^ Javid Hussain,^2^ Ahmed Al-Harrasi ^2^, Najat Marraiki,^7^ and Nouf S. S. Zaghloul,^8^

*^1^ Institute of Chemical Sciences, Bahauddin Zakariya University, Multan-60800, Pakistan*

*^2^Department of Biological Science and ChemistFry, College of Arts and Sciences, University of Nizwa, Post Box 33, PC 616, Nizwa, Oman*

*^3^Department of Food Science and Human Nutrition, College of Applied and Health Sciences,* *A’Sharqiyah University, P. O. Box 42, Ibra, Oman*

*^4^Department of Chemistry, Khwaja Fareed University of Engineering & Information Technology, Rahim Yar Khan, 64200, Pakistan*

*^5^Departamento de Química Fundamental, Instituto de Química,* *Universidade de São Paulo, Avenida Professor LineuPrestes, 748, São Paulo 05508-000, Brazil*

*^6^Department of Chemistry, University of Okara, Okara-56300, Pakistan*

*^7^Department of Botany and Microbiology, College of Science, King Saud University, P.O. 2455, Riyadh, 11451, Saudi Arabia*

*^8^Bristol Centre for Functional Nanomaterials, HH Wills Physics Laboratory, Tyndall Avenue, University of Bristol, Bristol, BS8 1FD, UK*

# UV-vis absorption spectra

##
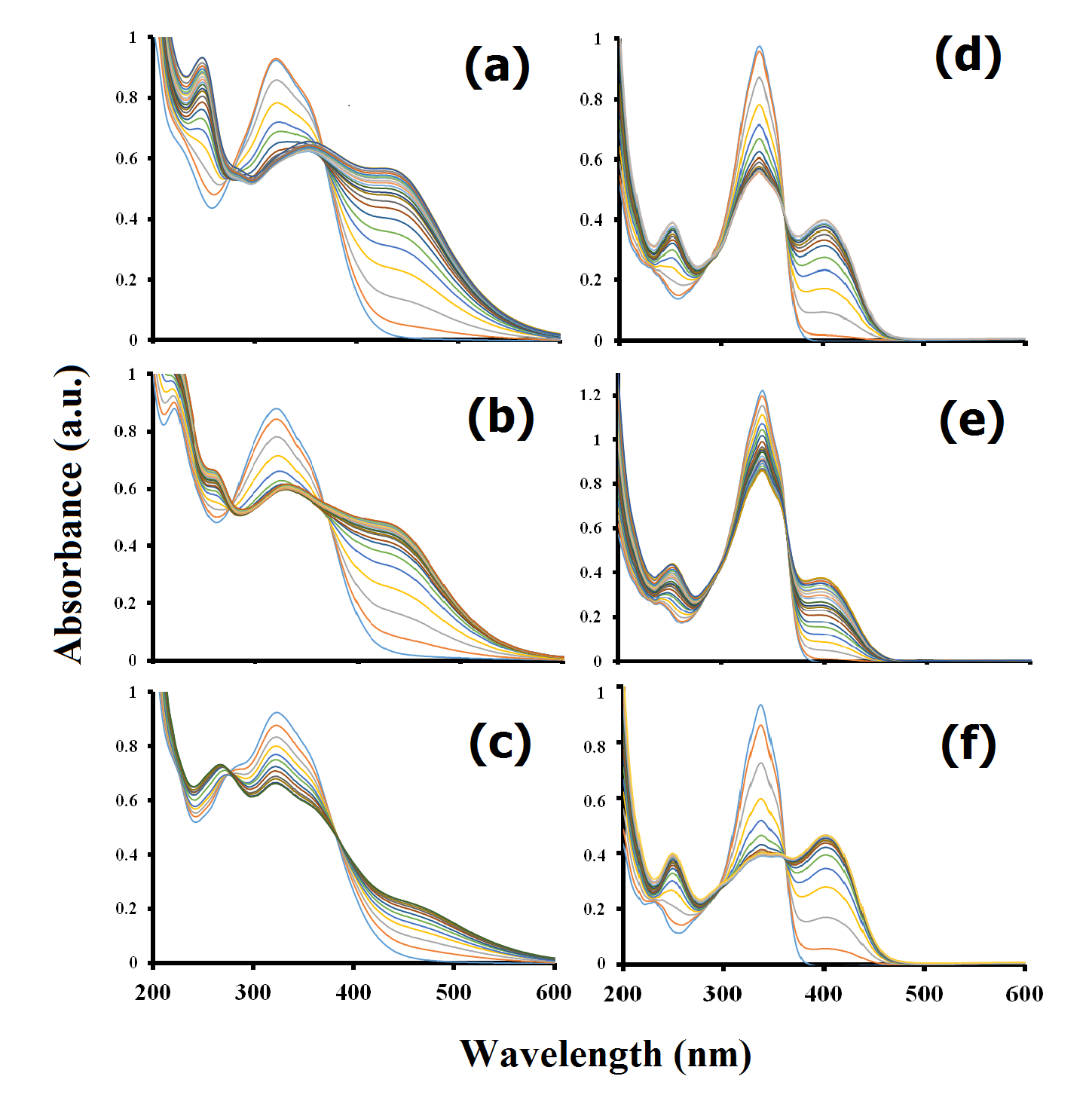
 Figure S1. Changes inthe UV-vis absorption of Receptor compounds 1-6 (3ml, 3 x 10^-5^M) was plotted against incremental addition of TBAF (1 x 10^-2^M).

# Job’s Plot

**
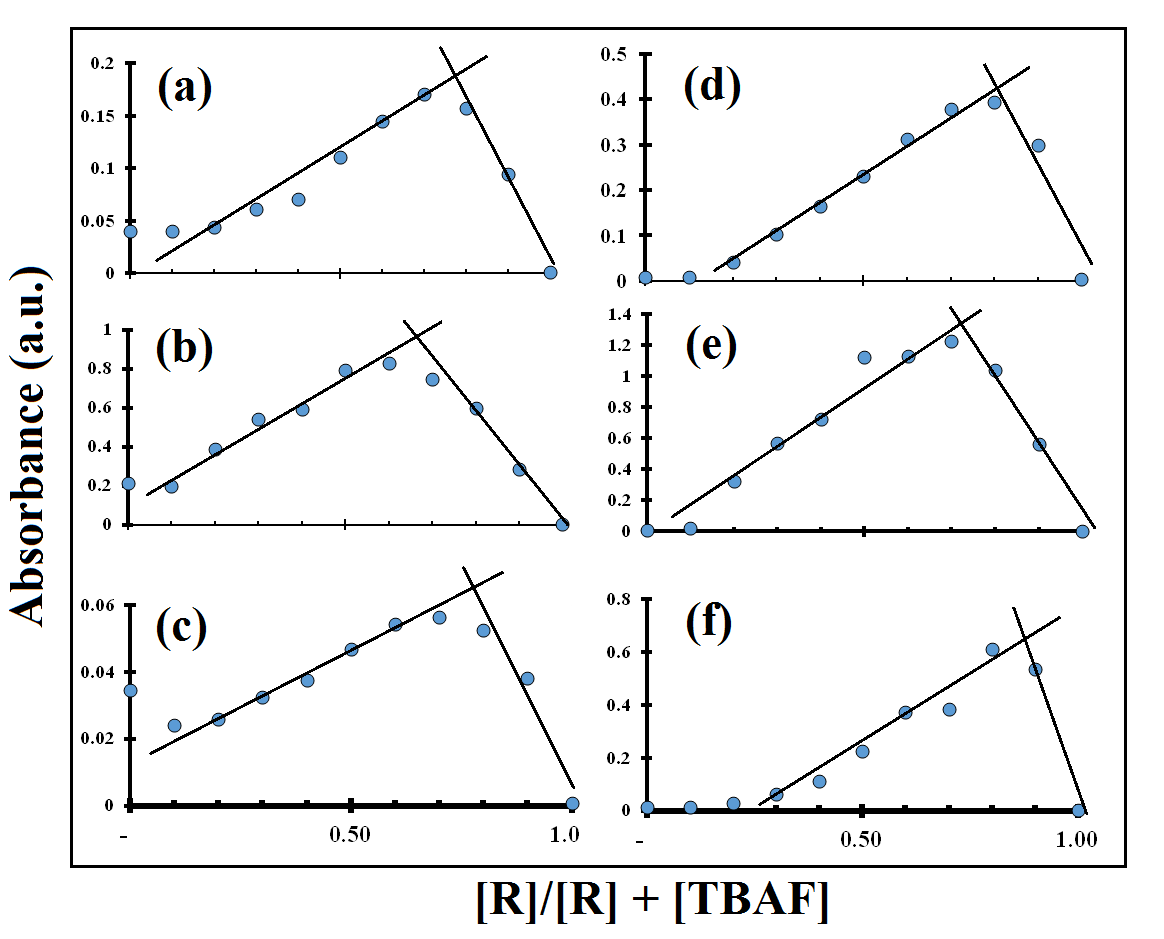
**

**Figure S2. Job’s Plot for anion ligand interaction of compound determined by UV-visible spectrophotometry in acetonitrile (TBAF: Ligand) (2:1) for 1 -6(a -f) respectively.**

# NMR Spectra

**
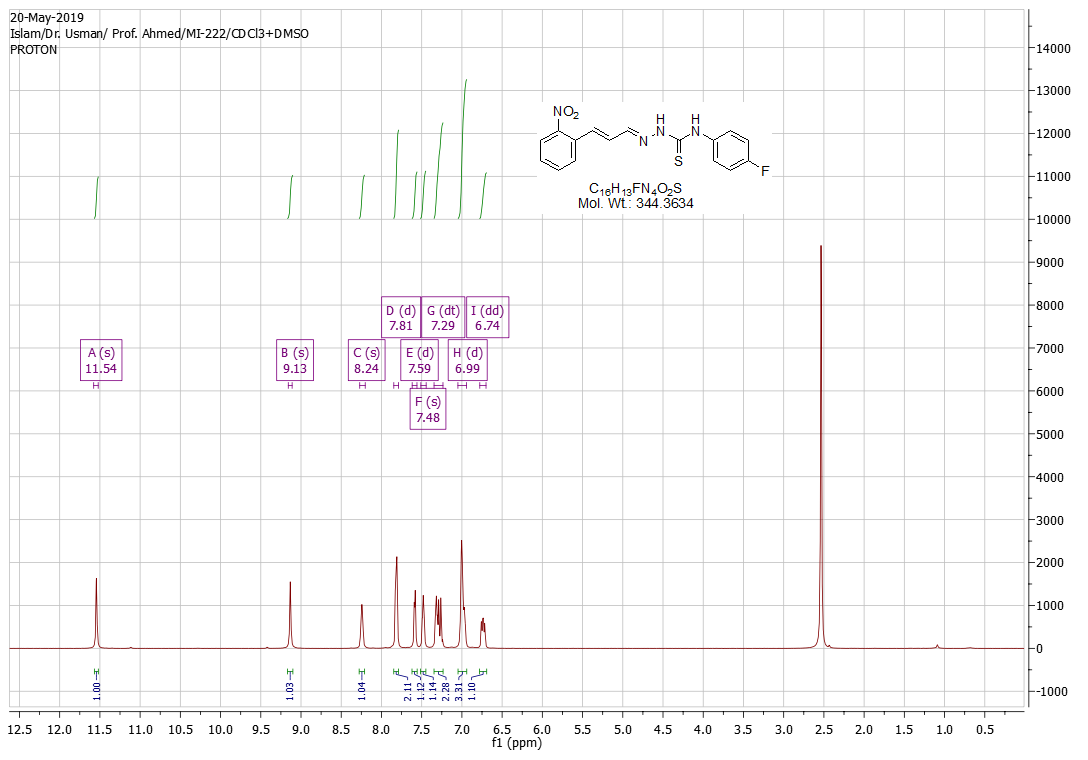

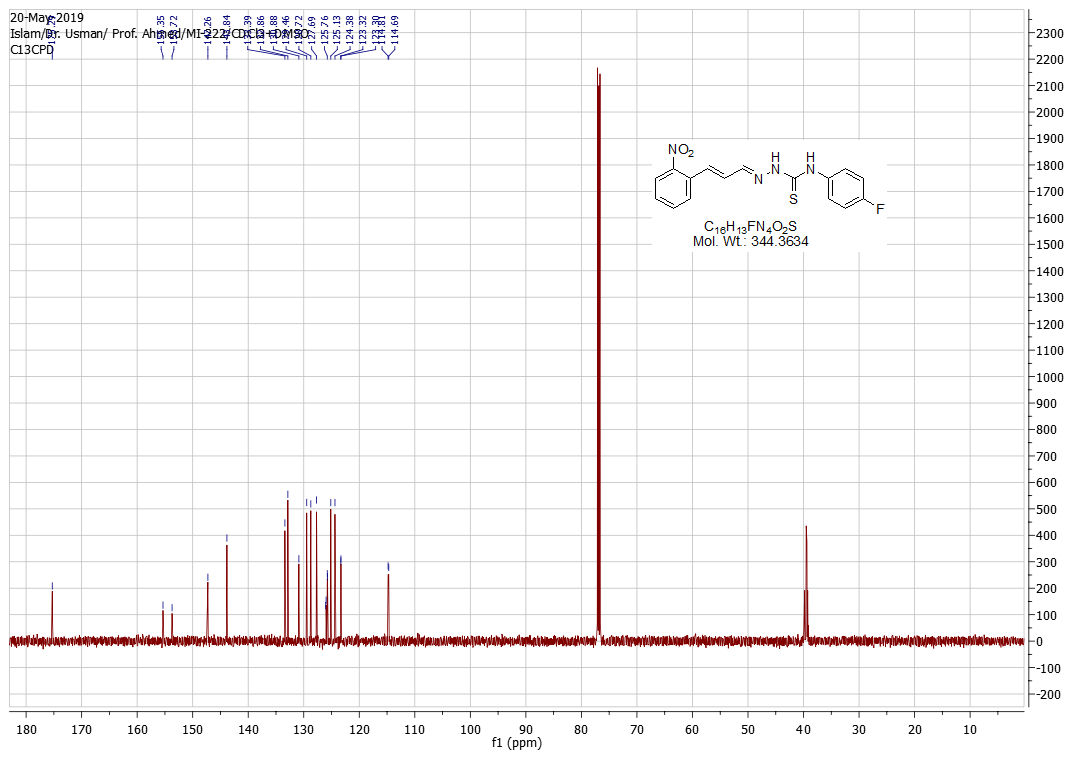
**

1

1

## Figure S3. Characterization of compound 1 by ^1^HNMR (600 MHz, Chloroform-d), ^13^CNMR (151 MHz, Chloroform-d)

**
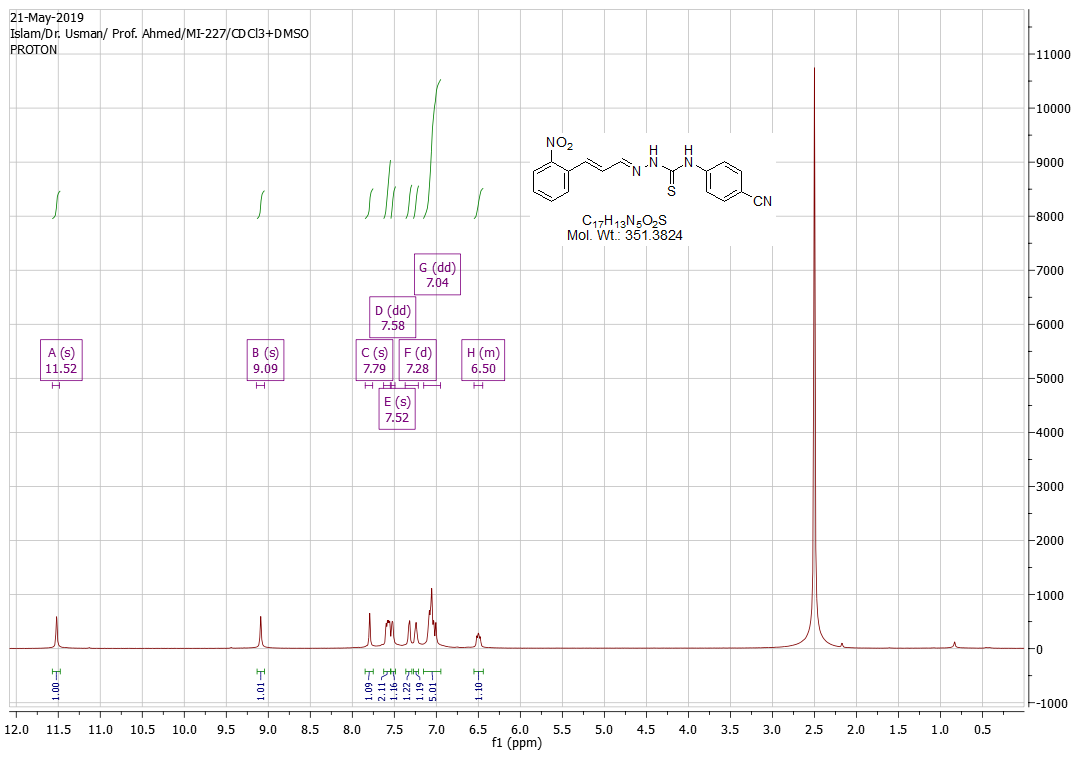

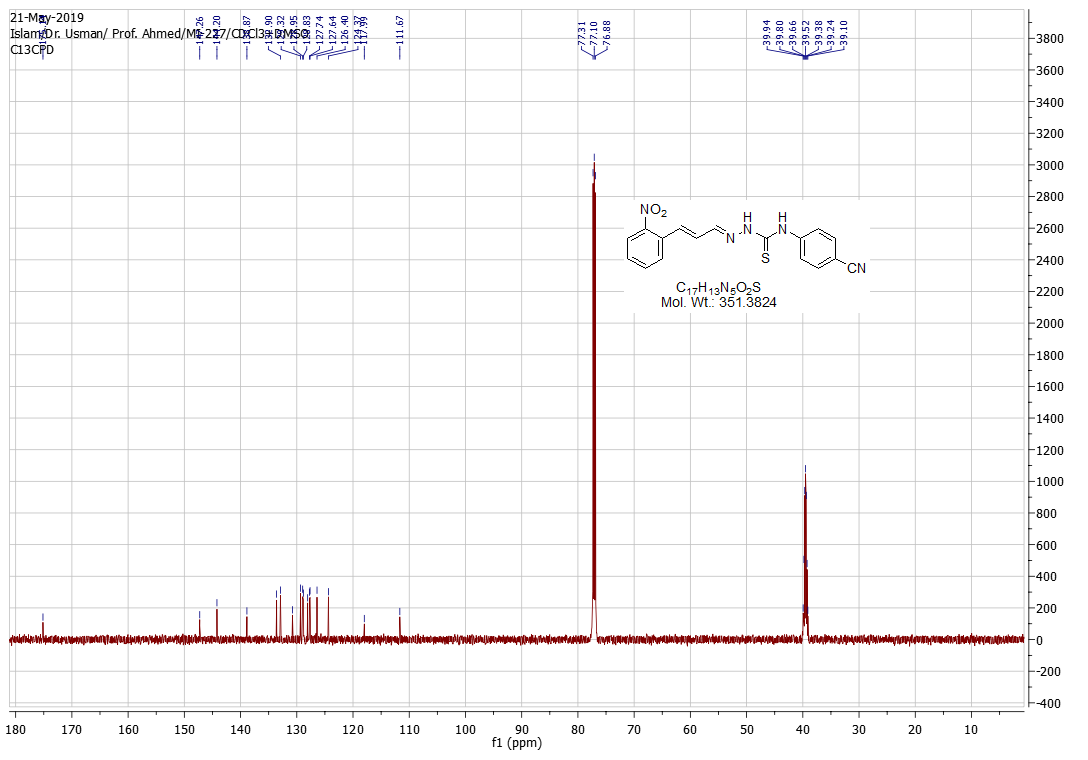
**

2

2

## Figure S4. Characterization of compound 2 by ^1^HNMR (600 MHz, Chloroform-d), ^13^CNMR (151 MHz, Chloroform-d)

##
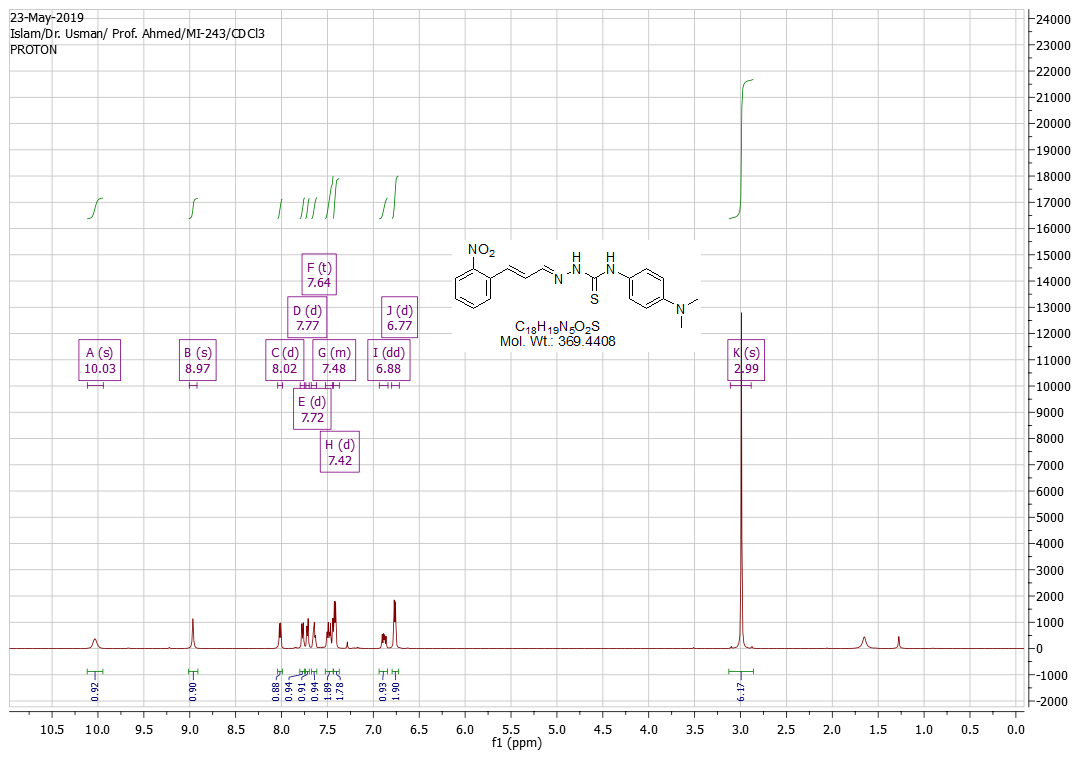

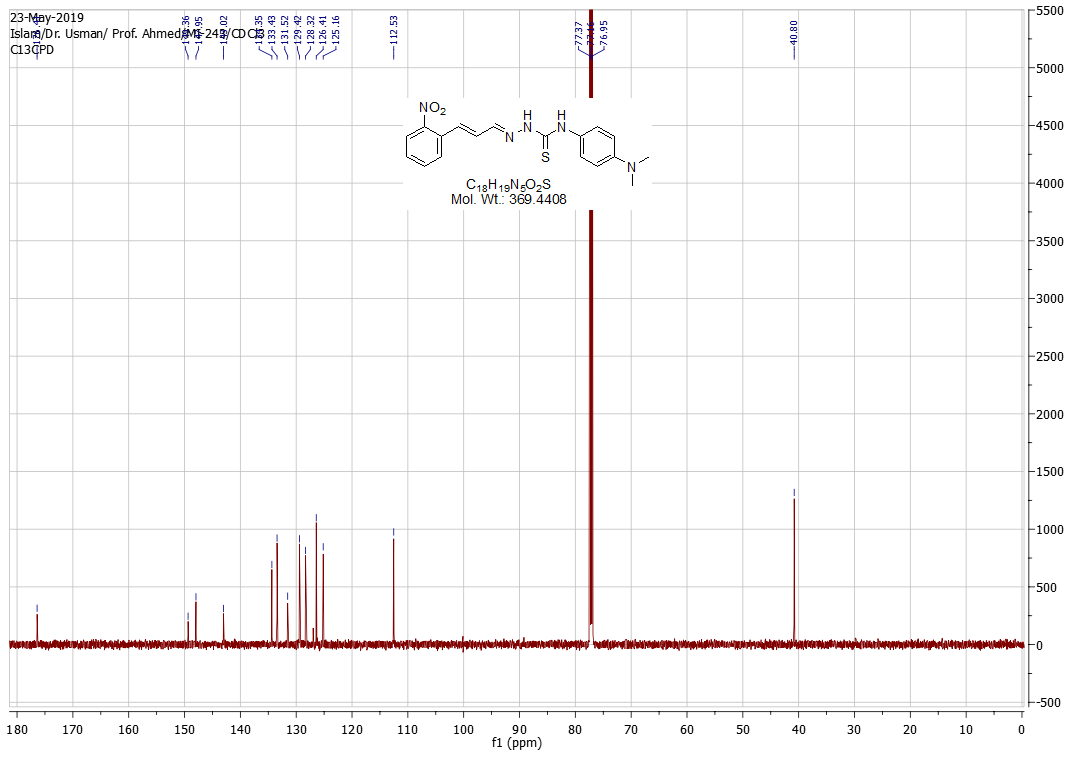
 Figure S5. Characterization of compound 3 by ^1^HNMR (600 MHz, Chloroform-d), ^13^CNMR (151 MHz, Chloroform-d)

3

3

##
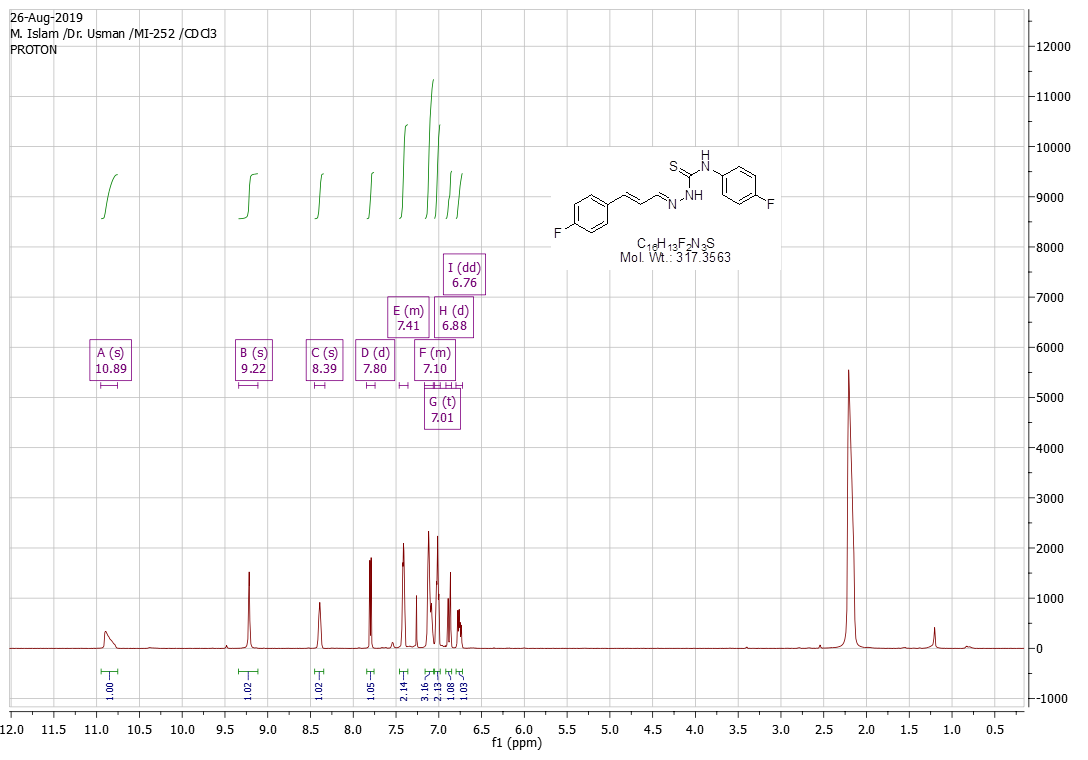

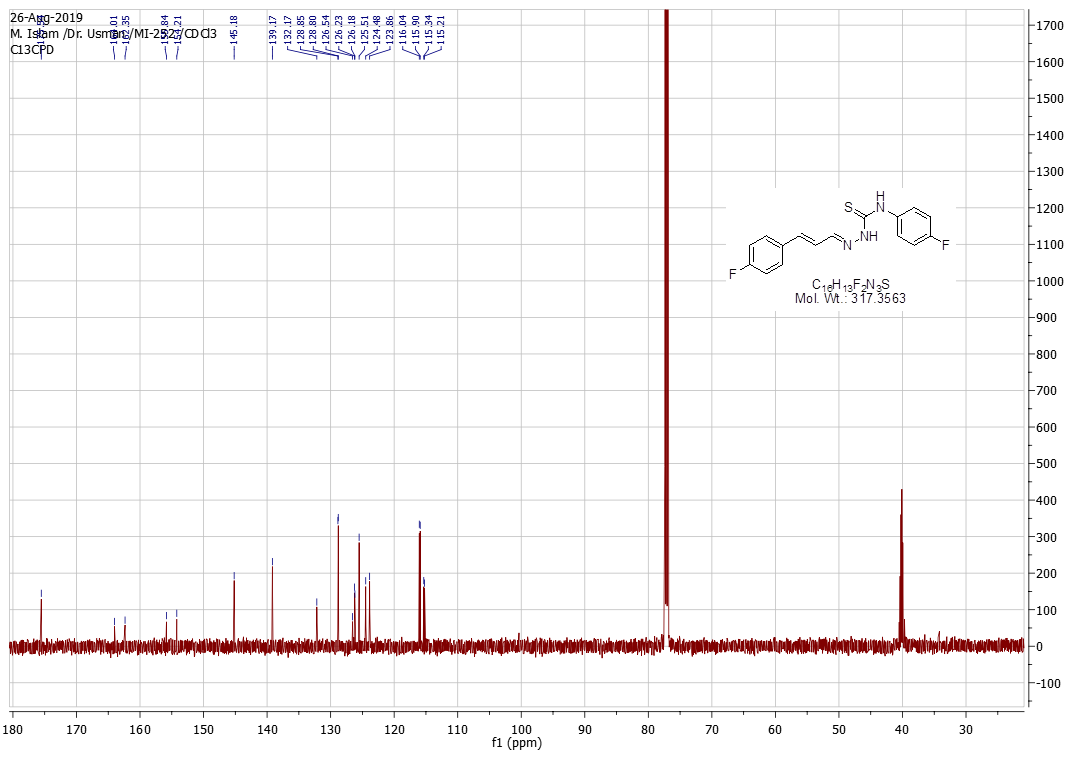
 Figure S6. Characterization of compound 4 by ^1^HNMR (600 MHz, Chloroform-d), ^13^CNMR (151 MHz, Chloroform-d)

4

4

##
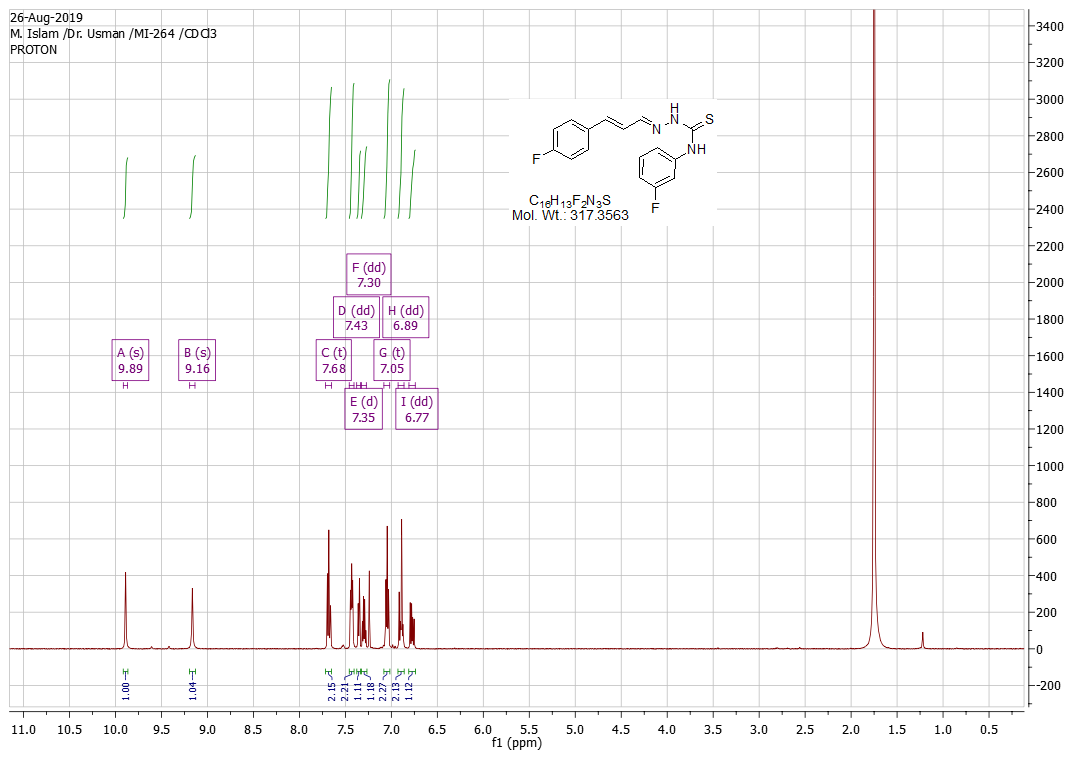

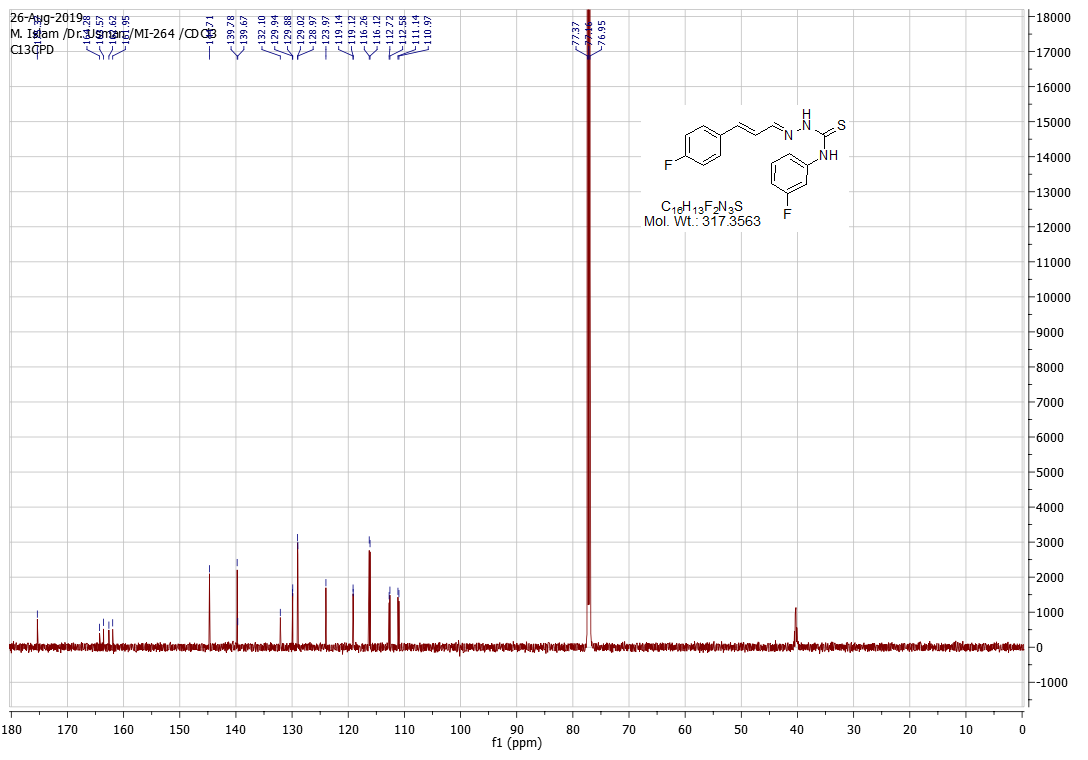
 Figure S7. Characterization of compound 5 by ^1^HNMR (600 MHz, Chloroform-d), ^13^CNMR (151 MHz, Chloroform-d)

5

5

##
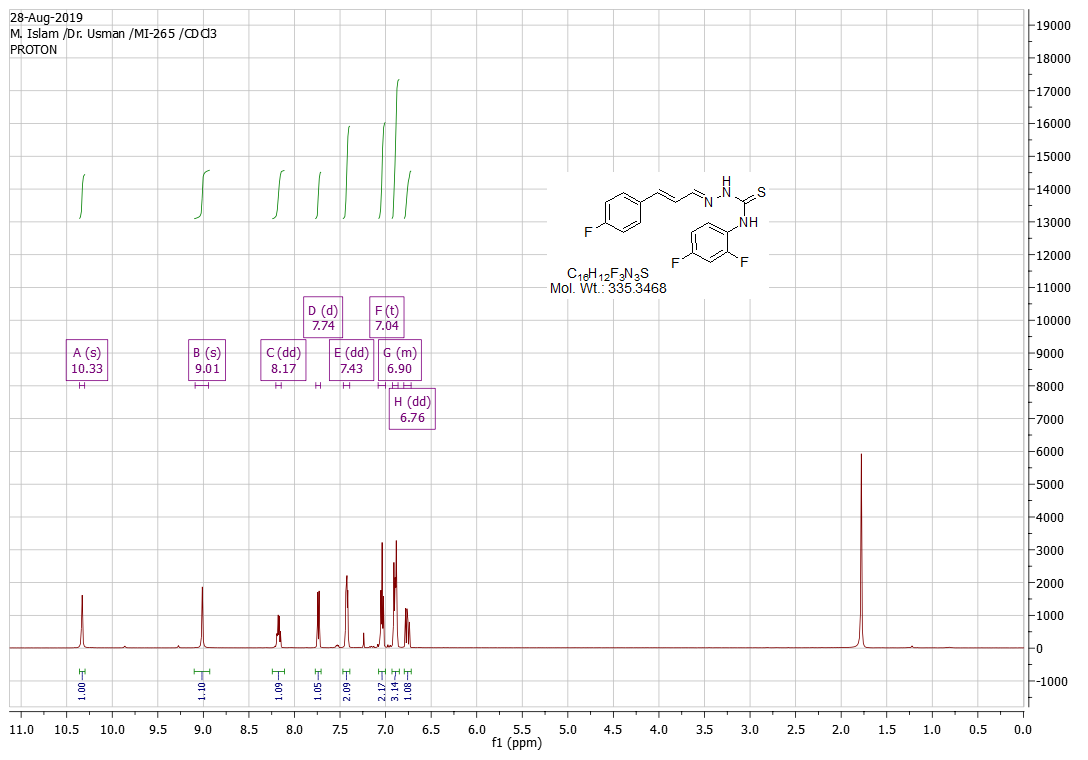

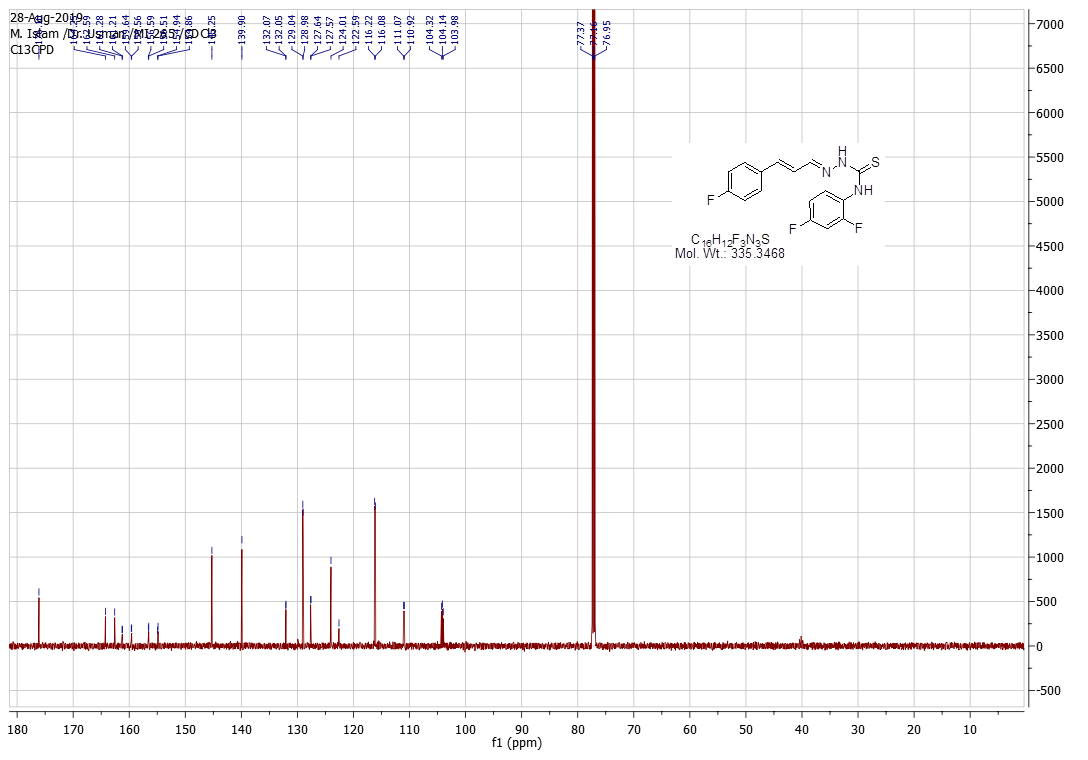
 Figure S8. Characterization of compound 6 by ^1^HNMR (600 MHz, Chloroform-d), ^13^CNMR (151 MHz, Chloroform-d)

6

6

**
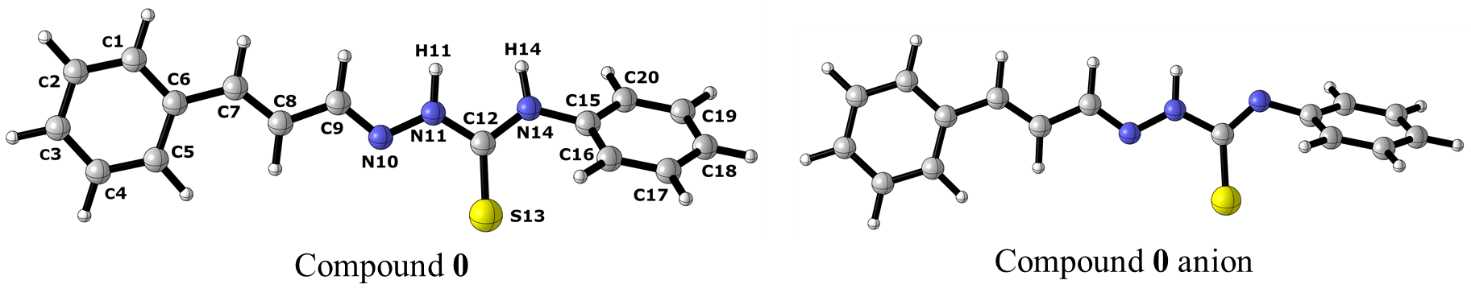
**

**Figure S9. Scheme with the optimized geometries with B3LYP-D3/6-311+(d,p)/SMD method of compound 0 and its anion.**

**
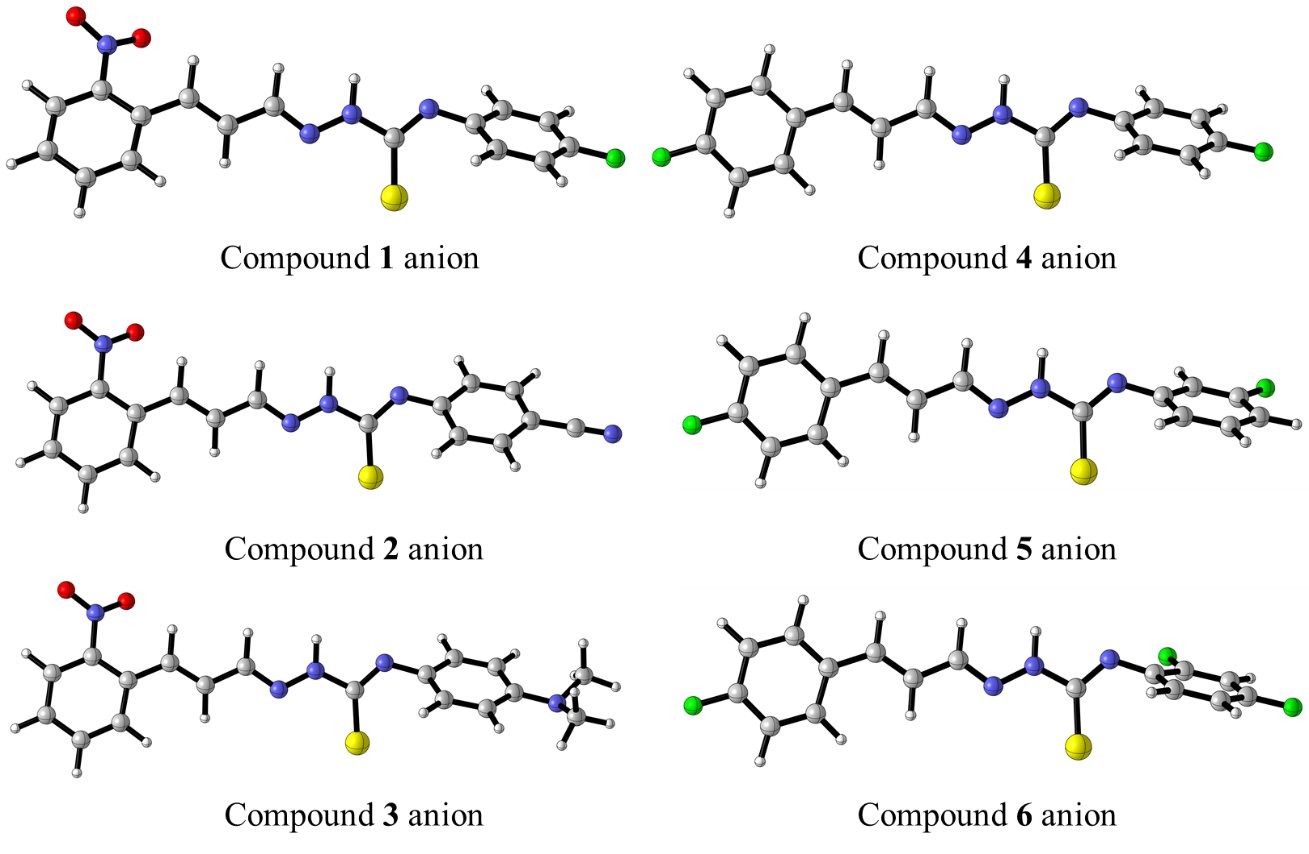
**

**Figure S10. Scheme with the optimized geometries with B3LYP-D3/6-311+(d,p)/SMD method of compounds 1-6 anions.**

**
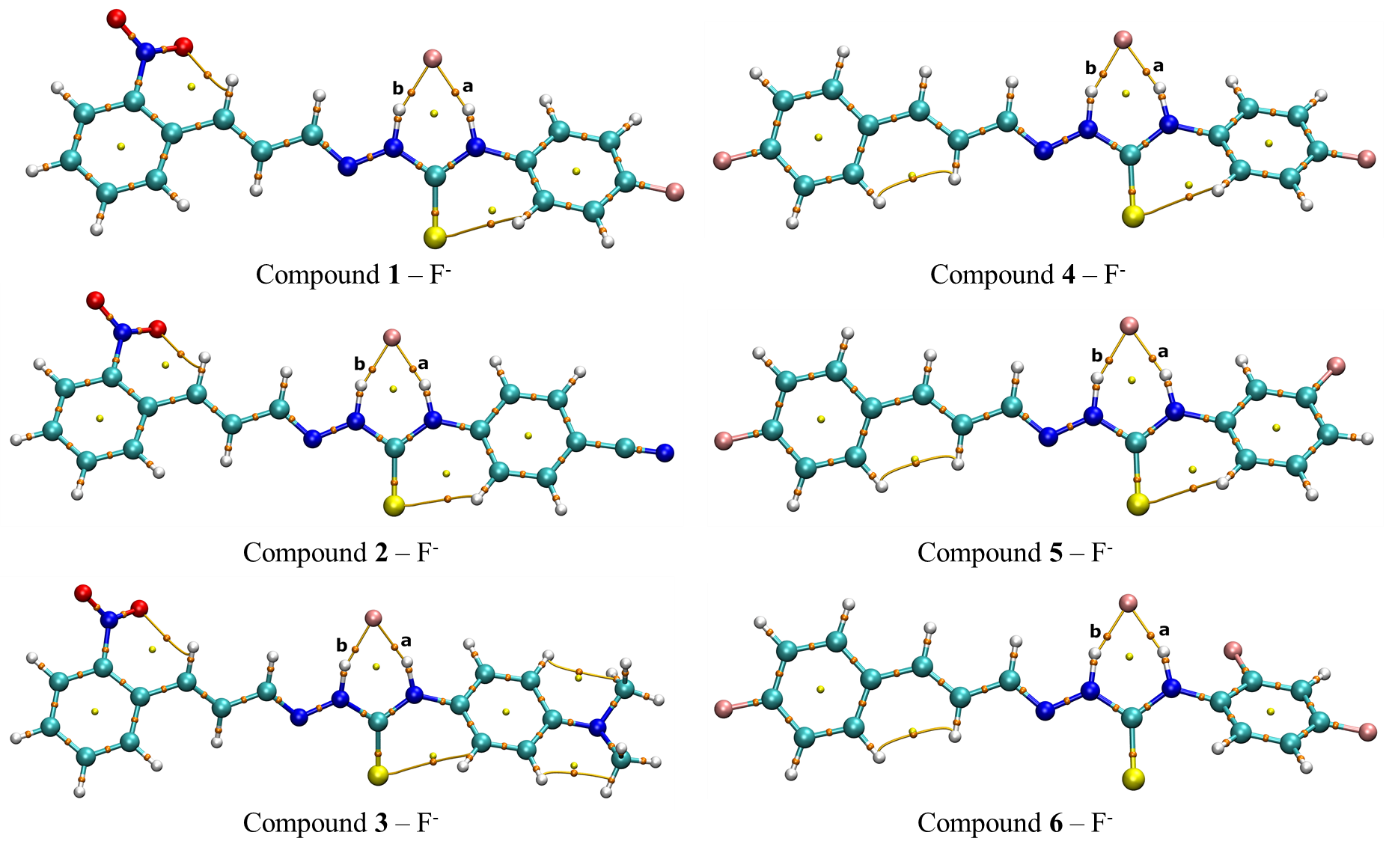
**

**Figure-S11: AIM molecular graphs of optimized geometries with B3LYP-D3/6-311+(d,p)/SMD method of compounds 1-6 complexed with anion F^-^. Tiny orange spheres (BCPs), tiny yellow spheres (RCP) and orange lines (BPs). H atoms (white spheres), C atoms (light blue spheres), N atoms (blue spheres), O atoms (red spheres), F atoms (pink spheres), and S atoms (yeallow spheres).**

**
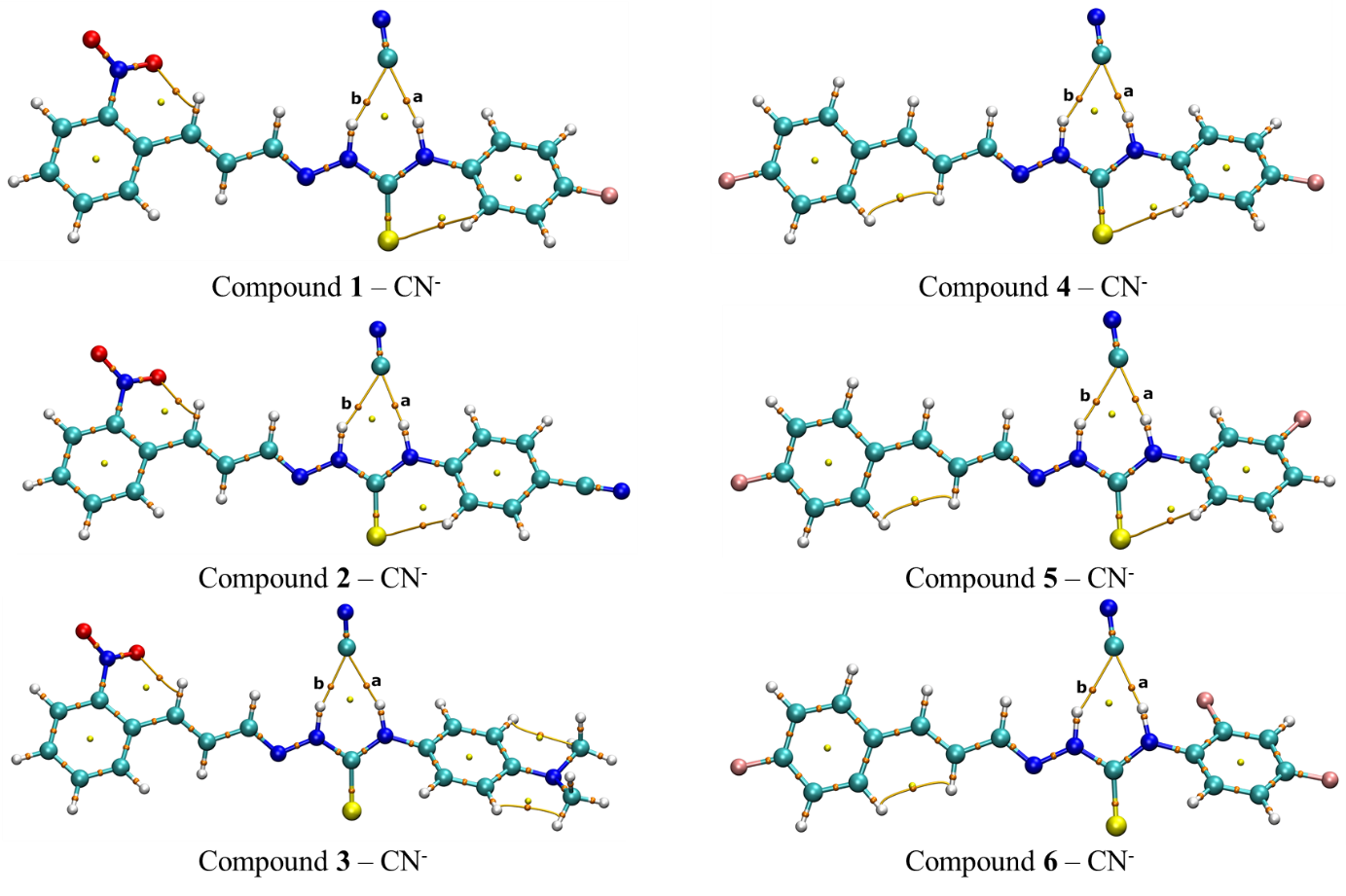
**

**Figure-S12: AIM molecular graphs of optimized geometries with B3LYP-D3/6-311+(d,p)/SMD method of compounds 1-6 complexed with anion CN^-^. Tiny orange spheres (BCPs), tiny yellow spheres (RCP) and orange lines (BPs). H atoms (white spheres), C atoms (light blue spheres), N atoms (blue spheres), O atoms (red spheres), F atoms (pink spheres), and S atoms (yeallow spheres).**

**
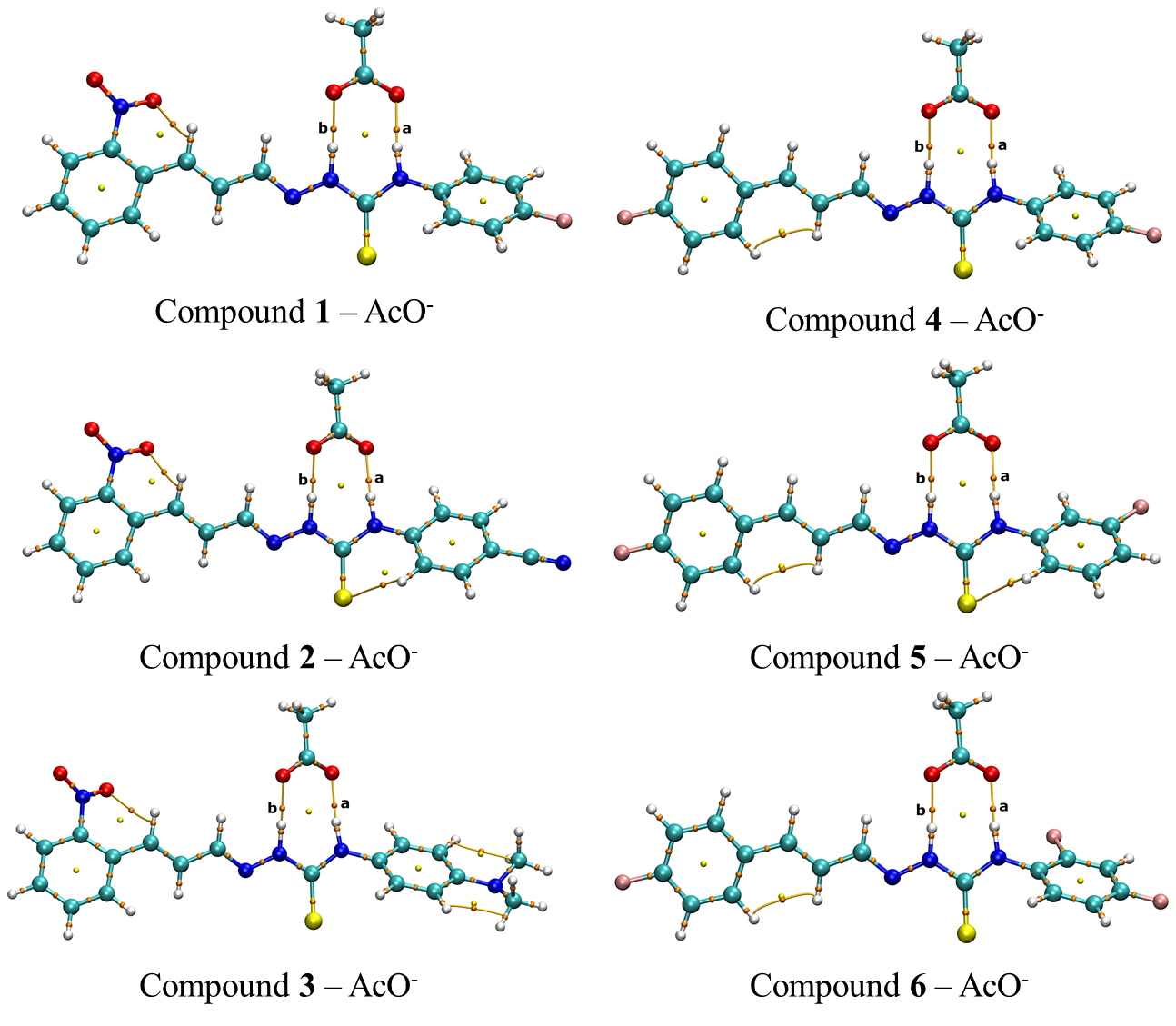
**

**Figure-S13: AIM molecular graphs of optimized geometries with B3LYP-D3/6-311+(d,p)/SMD method of compounds 1-6 complexed with anion AcO^-^. Tiny orange spheres (BCPs), tiny yellow spheres (RCP) and orange lines (BPs). H atoms (white spheres), C atoms (light blue spheres), N atoms (blue spheres), O atoms (red spheres), F atoms (pink spheres), and S atoms (yeallow spheres).**

**Table S1. Scheme with the frontier molecular orbitals of compounds 0 and 1-6 with their respective energy of HOMO→LUMO excitation ΔE (eV).**

| **Sensor** | **FMO** |  | **ΔE (eV)** |
| --- | --- | --- | --- |
| **0** | **LUMO** | **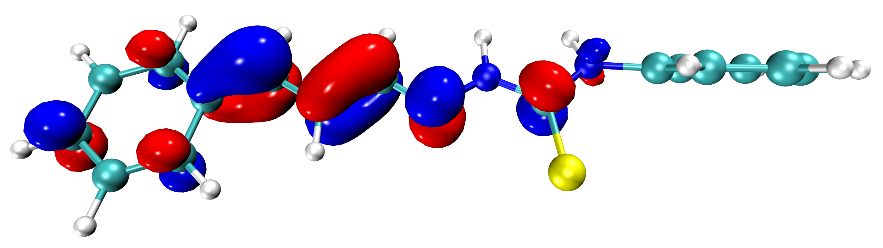** | **7.64** |
|  | **HOMO** | **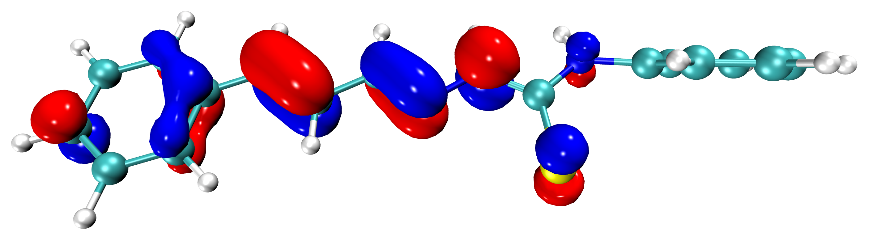** |  |
| **1** | **LUMO** | **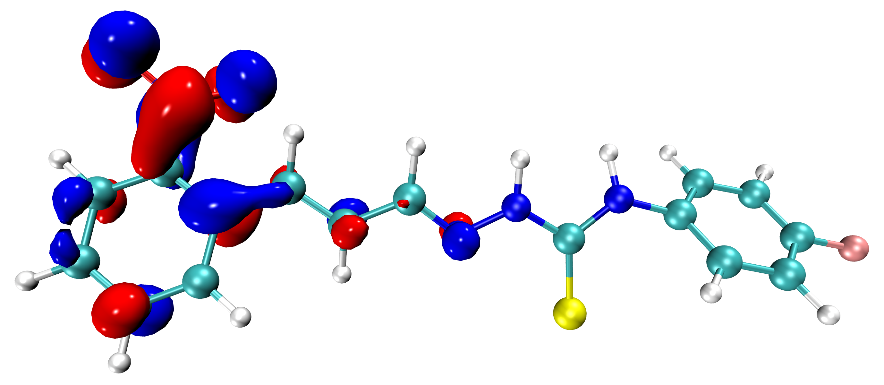** | **7.19** |
|  | **HOMO** | **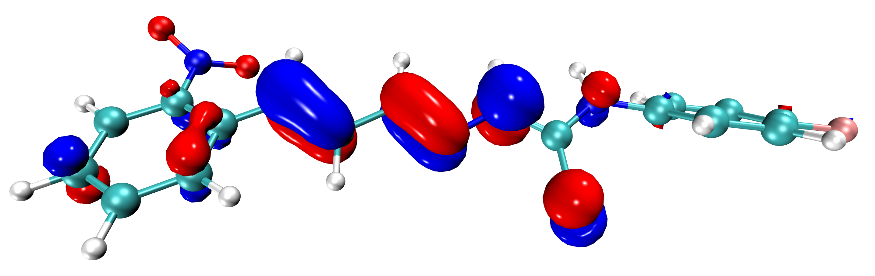** |  |
| **2** | **LUMO** | **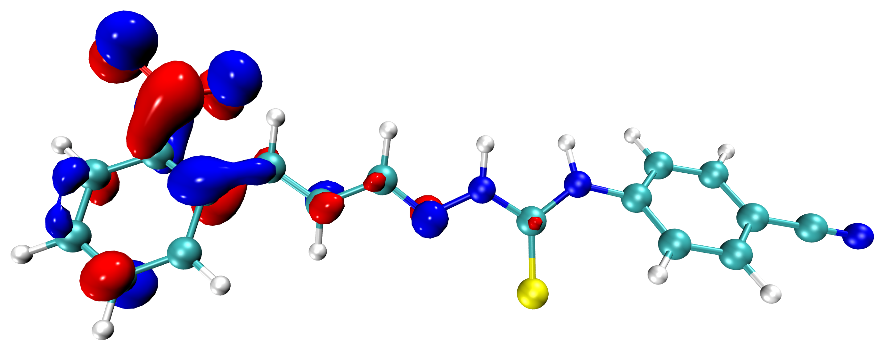** | **7.23** |
|  | **HOMO** | **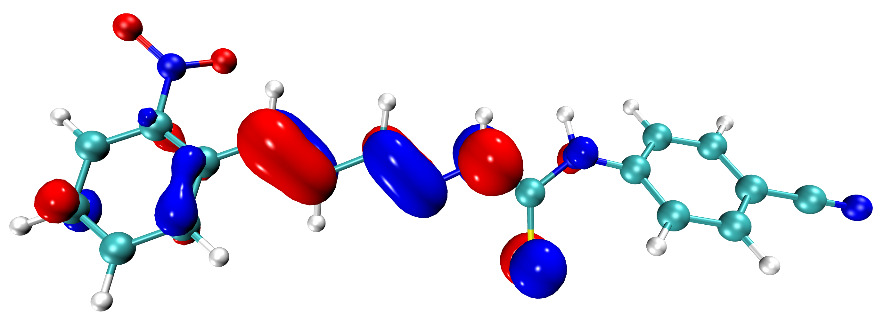** |  |
| **3** | **LUMO** | **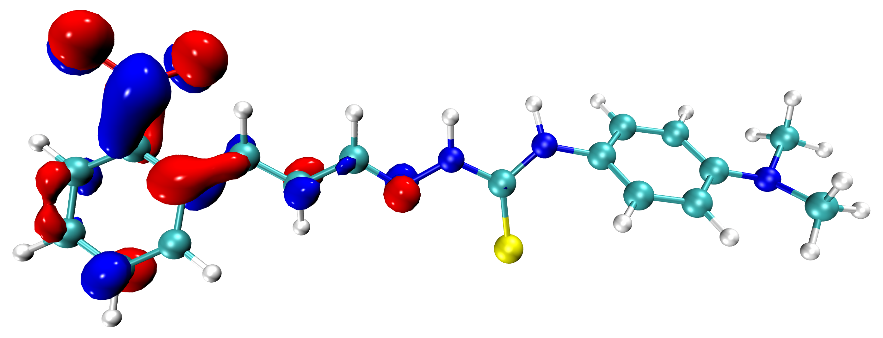** | **6.61** |
|  | **HOMO** | **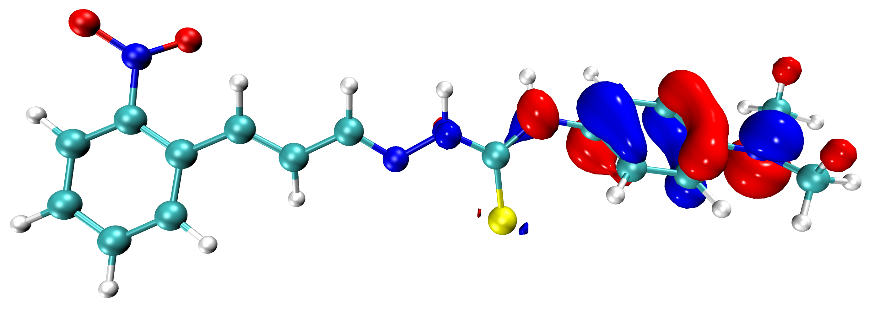** |  |
| **4** | **LUMO** | **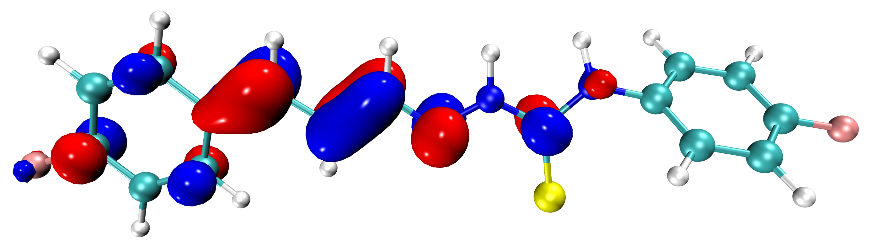** | **7.67** |
|  | **HOMO** | **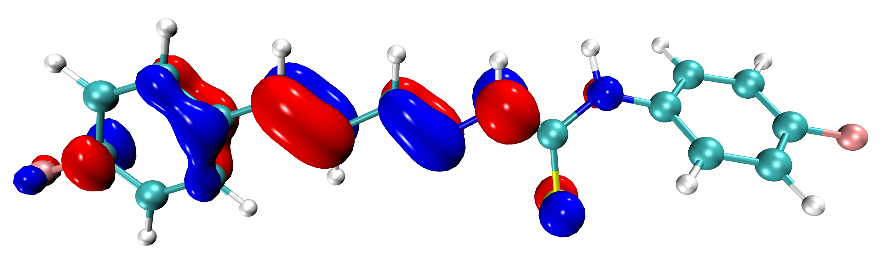** |  |
| **5** | **LUMO** | **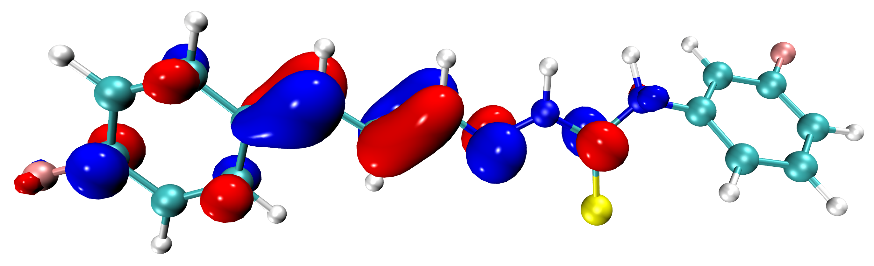** | **7.64** |
|  | **HOMO** | **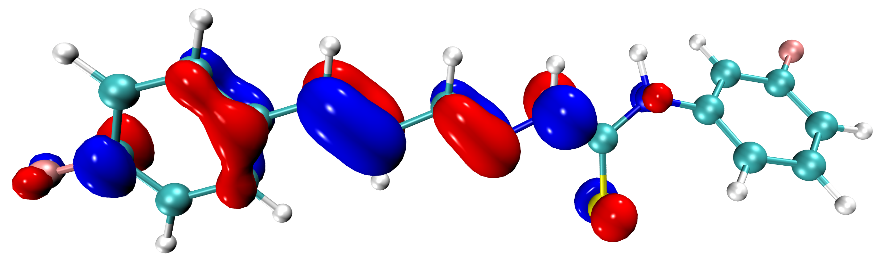** |  |
| **6** | **LUMO** | **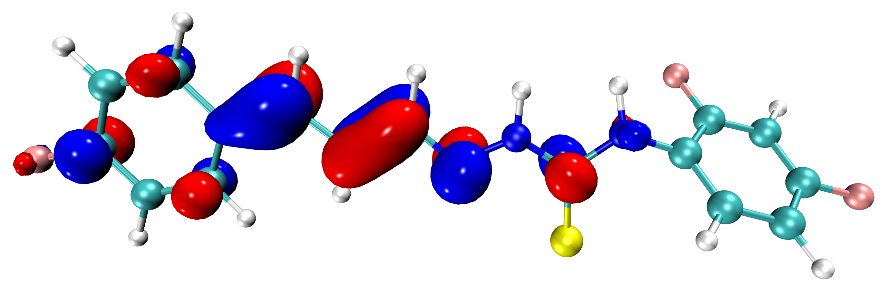** | **7.68** |
|  | **HOMO** | **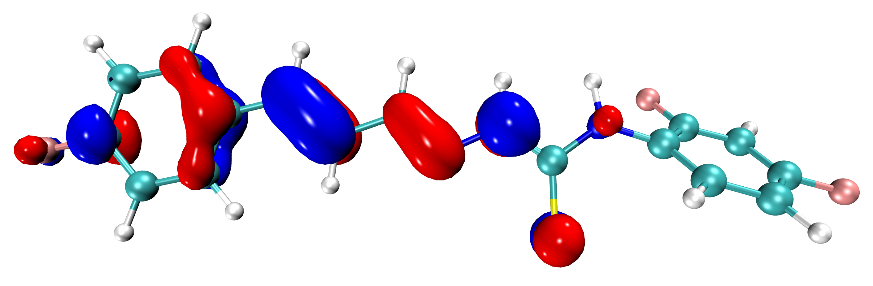** |  |

**Table S2.Absorption spectrum via transition electric dipole moments for compound 0. Calculated with ωB97X-D3/ma-def2-TZVP with implicit solvent SMD (water).**

| **State** | **Wavelength (nm)** | ***f*_osc_** | **Energy (eV)** |
| --- | --- | --- | --- |
| **1** | **331.9** | **0.0076** | **3.735** |
| **2** | **298.1** | **1.9609** | **4.160** |
| **3** | **260.2** | **0.3612** | **4.764** |
| **4** | **247.5** | **0.0069** | **5.010** |
| **5** | **235.5** | **0.0062** | **5.265** |
| **6** | **235.8** | **0.0011** | **5.257** |
| **7** | **225.1** | **0.0584** | **5.507** |
| **8** | **216.2** | **0.0913** | **5.734** |
| **9** | **210.7** | **0.0367** | **5.884** |
| **10** | **209.8** | **0.0262** | **5.908** |

**Table S3. Absorption spectrum via transition electric dipole moments for compound 1. Calculated with ωB97X-D3/ma-def2-TZVP with implicit solvent SMD (water).**

| **State** | **Wavelength (nm)** | ***f*_osc_** | **Energy (eV)** |
| --- | --- | --- | --- |
| **1** | **332.3** | **0.6711** | **3.731** |
| **2** | **331.8** | **0.0528** | **3.737** |
| **3** | **313.9** | **0.1429** | **3.950** |
| **4** | **279.6** | **0.8329** | **4.435** |
| **5** | **270.9** | **0.0061** | **4.577** |
| **6** | **259.8** | **0.4190** | **4.773** |
| **7** | **252.2** | **0.2454** | **4.916** |
| **8** | **238.6** | **0.0249** | **5.196** |
| **9** | **244.3** | **0.0031** | **5.075** |
| **10** | **332.3** | **0.6711** | **3.731** |

**Table S4. Absorption spectrum via transition electric dipole moments for compound 2. Calculated with ωB97X-D3/ma-def2-TZVP with implicit solvent SMD (water).**

| **State** | **Wavelength (nm)** | ***f*_osc_** | **Energy (eV)** |
| --- | --- | --- | --- |
| **1** | **348.4** | **0.0083** | **3.559** |
| **2** | **331.8** | **0.8935** | **3.736** |
| **3** | **314.0** | **0.1611** | **3.948** |
| **4** | **282.1** | **1.0072** | **4.394** |
| **5** | **274.7** | **0.4131** | **4.513** |
| **6** | **270.9** | **0.0019** | **4.577** |
| **7** | **252.8** | **0.1358** | **4.905** |
| **8** | **245.2** | **0.0021** | **5.056** |
| **9** | **244.2** | **0.0013** | **5.078** |
| **10** | **229.8** | **0.0196** | **5.395** |

**Table S5. Absorption spectrum via transition electric dipole moments for compound 3. Calculated with ωB97X-D3/ma-def2-TZVP with implicit solvent SMD (water).**

| **State** | **Wavelength (nm)** | ***f*_osc_** | **Energy (eV)** |
| --- | --- | --- | --- |
| **1** | **326.5** | **0.7900** | **3.797** |
| **2** | **315.4** | **0.3036** | **3.931** |
| **3** | **322.7** | **0.0271** | **3.842** |
| **4** | **278.7** | **1.0664** | **4.449** |
| **5** | **271.6** | **0.0016** | **4.565** |
| **6** | **272.9** | **0.1024** | **4.543** |
| **7** | **263.1** | **0.0429** | **4.712** |
| **8** | **250.0** | **0.1417** | **4.960** |
| **9** | **245.1** | **0.0460** | **5.058** |
| **10** | **232.2** | **0.0478** | **5.340** |

**Table S6. Absorption spectrum via transition electric dipole moments for compound 4. Calculated with ωB97X-D3/ma-def2-TZVP with implicit solvent SMD (water).**

| **State** | **Wavelength (nm)** | ***f*_osc_** | **Energy (eV)** |
| --- | --- | --- | --- |
| **1** | **328.5** | **0.0104** | **3.774** |
| **2** | **296.3** | **1.9181** | **4.184** |
| **3** | **256.3** | **0.3458** | **4.837** |
| **4** | **246.7** | **0.0472** | **5.026** |
| **5** | **238.0** | **0.0236** | **5.210** |
| **6** | **235.3** | **0.0012** | **5.270** |
| **7** | **223.6** | **0.0506** | **5.545** |
| **8** | **216.2** | **0.0912** | **5.733** |
| **9** | **210.6** | **0.02000** | **5.886** |
| **10** | **209.9** | **0.0455** | **5.908** |

**Table S7. Absorption spectrum via transition electric dipole moments for compound 5. Calculated with ωB97X-D3/ma-def2-TZVP with implicit solvent SMD (water).**

| **State** | **Wavelength (nm)** | ***f*_osc_** | **Energy (eV)** |
| --- | --- | --- | --- |
| **1** | **335.0** | **0.0092** | **3.701** |
| **2** | **298.0** | **1.9503** | **4.161** |
| **3** | **260.5** | **0.3690** | **4.760** |
| **4** | **246.9** | **0.0296** | **5.023** |
| **5** | **235.9** | **0.0019** | **5.256** |
| **6** | **234.9** | **0.0025** | **5.279** |
| **7** | **224.8** | **0.0479** | **5.516** |
| **8** | **215.3** | **0.0766** | **5.759** |
| **9** | **211.0** | **0.0307** | **5.876** |
| **10** | **209.5** | **0.0138** | **5.917** |

**Table S8. Absorption spectrum via transition electric dipole moments for compound 6. Calculated with ωB97X-D3/ma-def2-TZVP with implicit solvent SMD (water).**

| **State** | **Wavelength (nm)** | ***f*_osc_** | **Energy (eV)** |
| --- | --- | --- | --- |
| **1** | **327.2** | **0.0048** | **3.790** |
| **2** | **295.7** | **1.9084** | **4.193** |
| **3** | **251.2** | **0.2632** | **4.936** |
| **4** | **246.2** | **0.1234** | **5.036** |
| **5** | **233.7** | **0.0341** | **5.305** |
| **6** | **234.0** | **0.0005** | **5.298** |
| **7** | **221.8** | **0.0582** | **5.590** |
| **8** | **214.1** | **0.1282** | **5.790** |
| **9** | **209.4** | **0.0127** | **5.921** |
| **10** | **210.0** | **0.0488** | **5.904** |

**Table S9. Plot of hole+electron surfaces of main excitations of compound 0. Hole is the blue surface and electron is the green surface.**

| **Excitation** | **hole+electron** | **Energy** |
| --- | --- | --- |
| **S_0_→S_1_** | **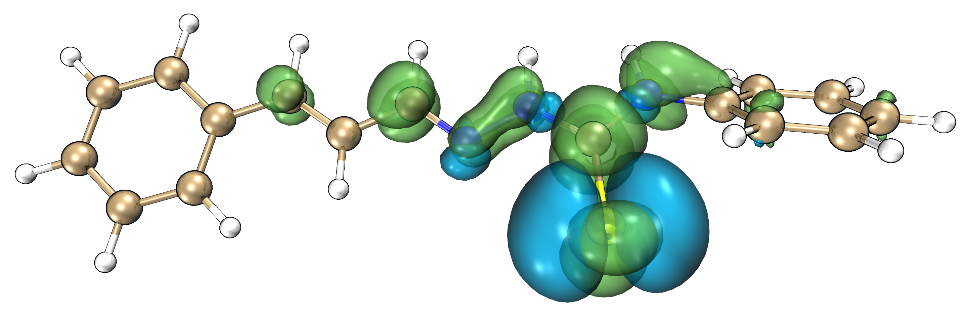** | **3.74 eV** |
| **S_0_→S_2_** | **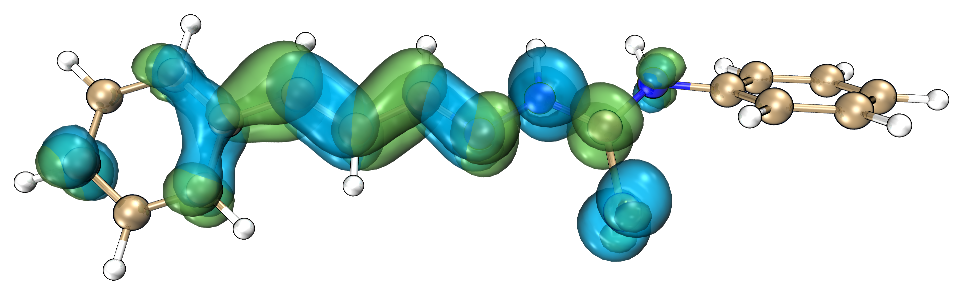** | **4.16 eV** |
| **S_0_→S_3_** | **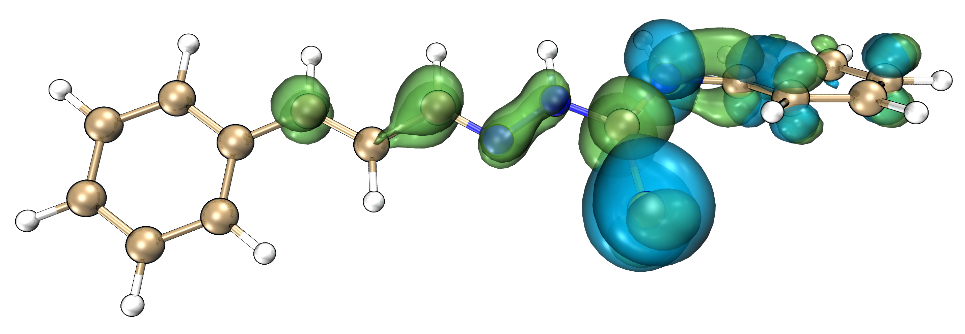** | **4.76 eV** |

**Table S10. Plot of hole+electron surfaces of main excitations of compound 1. Hole is the blue surface and electron is the green surface.**

| **Excitation** | **hole+electron** | **Energy** |
| --- | --- | --- |
| **S_0_→S_1_** | **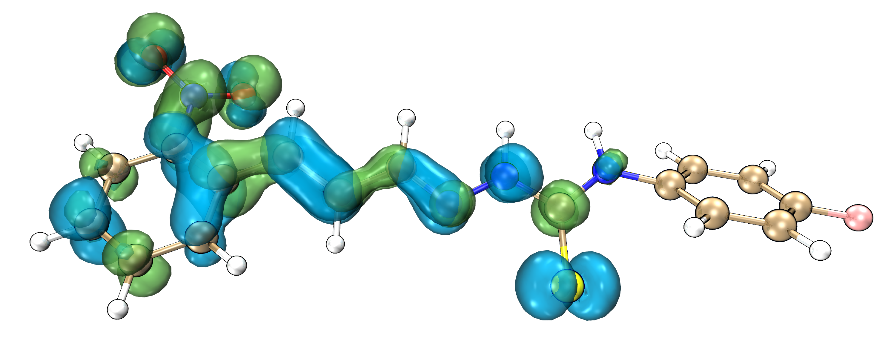** | **3.73 eV** |
| **S_0_→S_2_** | **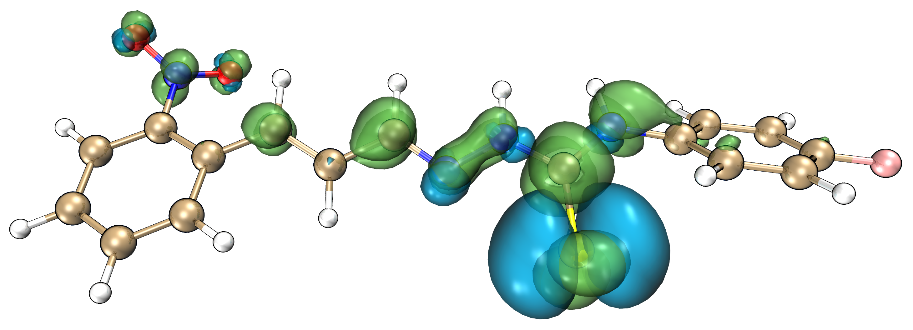** | **3.74 eV** |
| **S_0_→S_3_** | **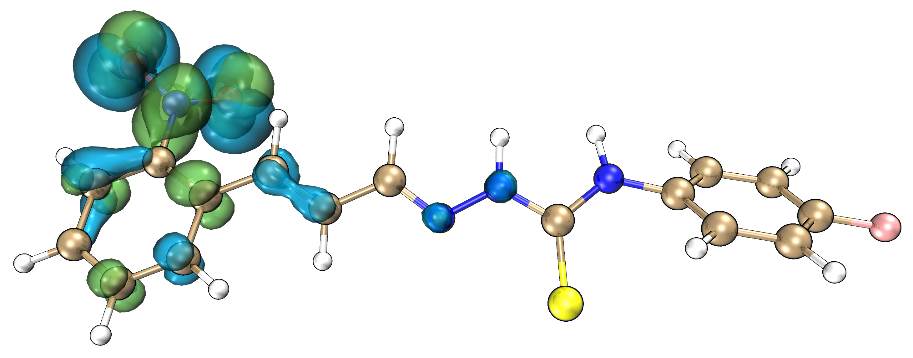** | **3.95 eV** |
| **S_0_→S_4_** | **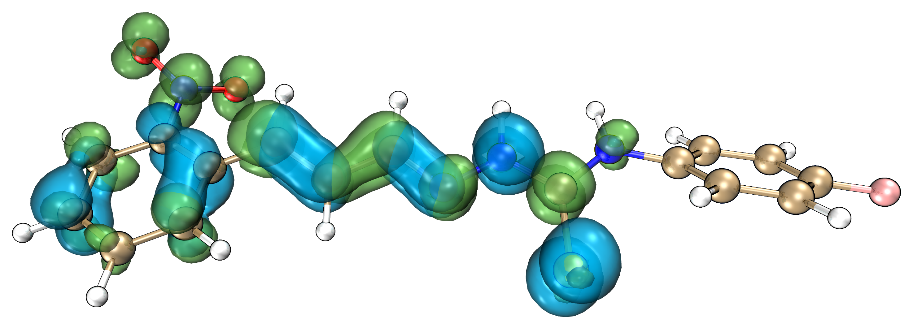** | **4.44 eV** |

**Table S11. Plot of hole+electron surfaces of main excitations of compound 2. Hole is the blue surface and electron is the green surface.**

| **Excitation** | **hole+electron** | **Energy** |
| --- | --- | --- |
| **S_0_→S_1_** | **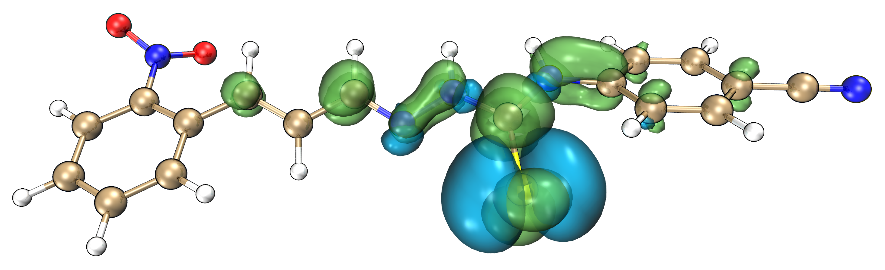** | **3.56 eV** |
| **S_0_→S_2_** | **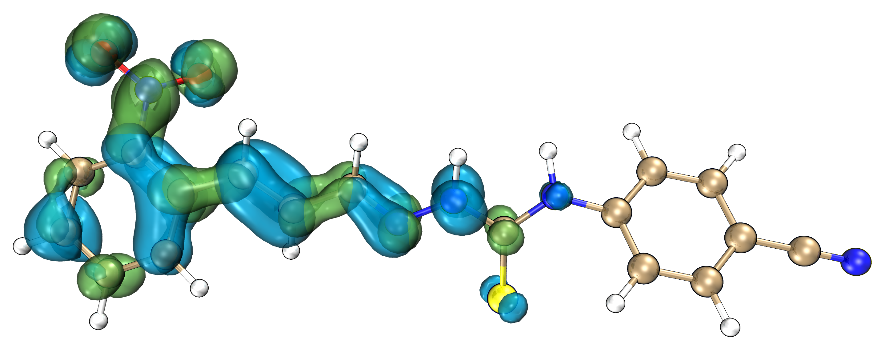** | **3.74 eV** |
| **S_0_→S_3_** | **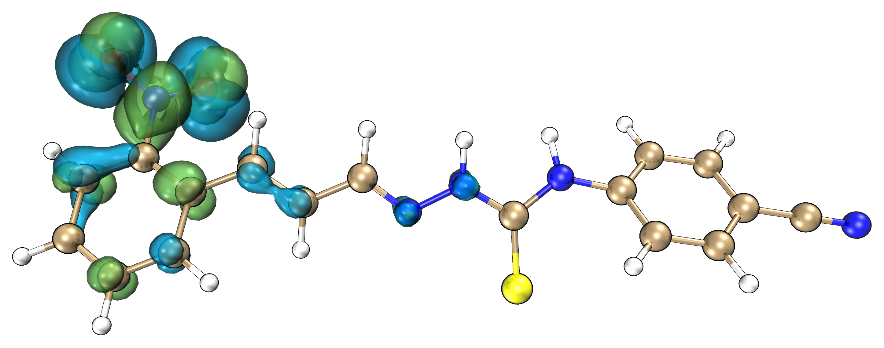** | **3.95 eV** |
| **S_0_→S_4_** | **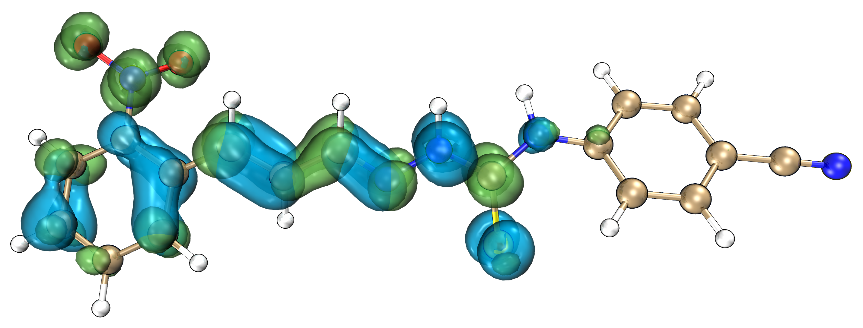** | **4.39 eV** |

**Table S12. Plot of hole+electron surfaces of main excitations of compound 3. Hole is the blue surface and electron is the green surface.**

| **Excitation** | **hole+electron** | **Energy** |
| --- | --- | --- |
| **S_0_→S_1_** | **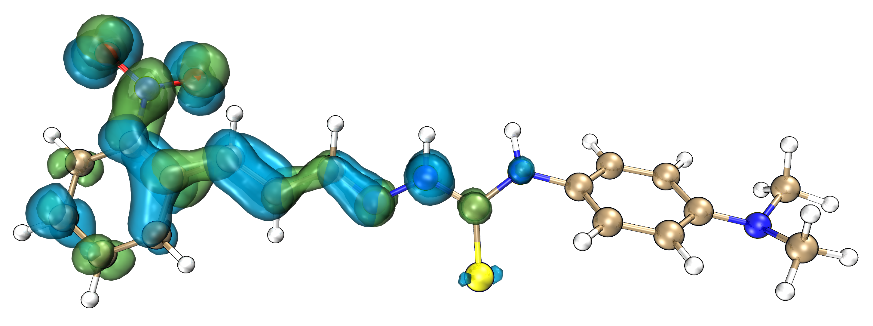** | **3.80 eV** |
| **S_0_→S_2_** | **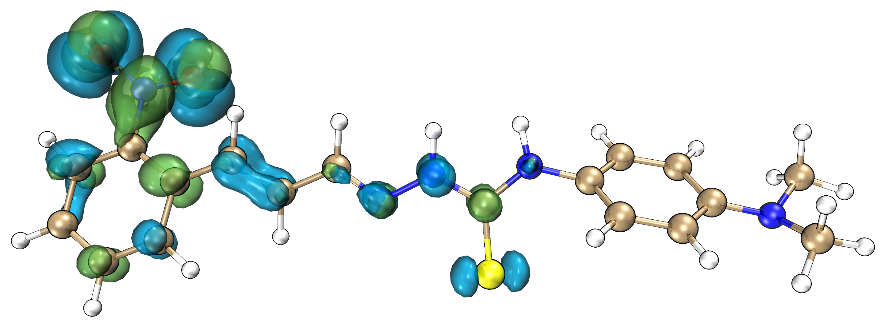** | **3.93 eV** |
| **S_0_→S_3_** | **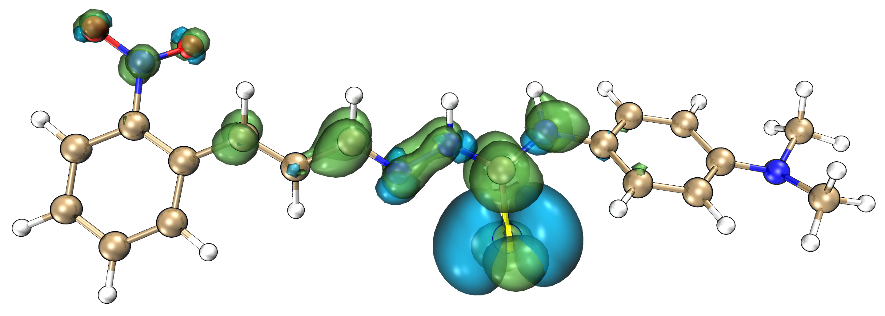** | **3.84 eV** |
| **S_0_→S_4_** | **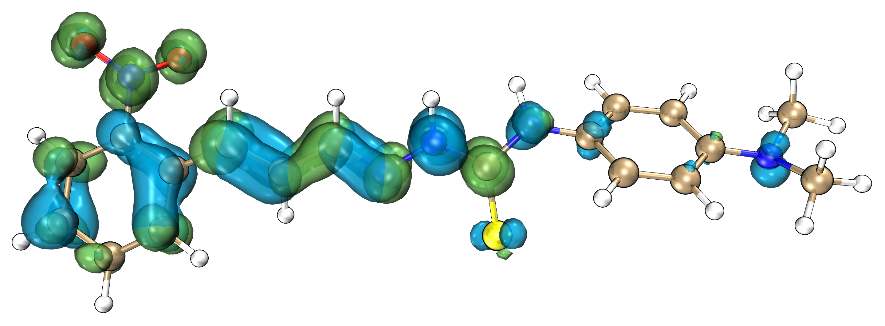** | **4.45 eV** |

**Table S13. Plot of hole+electron surfaces of main excitations of compound 4. Hole is the blue surface and electron is the green surface.**

| **Excitation** | **hole+electron** | **Energy** |
| --- | --- | --- |
| **S_0_→S_1_** | **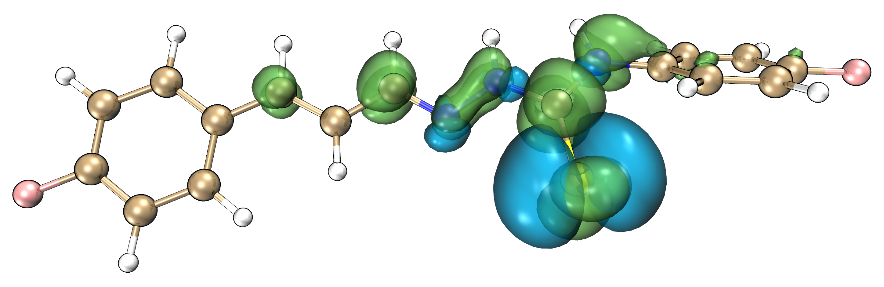** | **3.77 eV** |
| **S_0_→S_2_** | **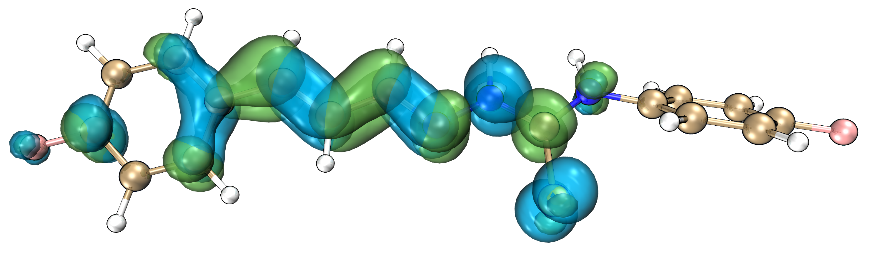** | **4.18 eV** |
| **S_0_→S_3_** | **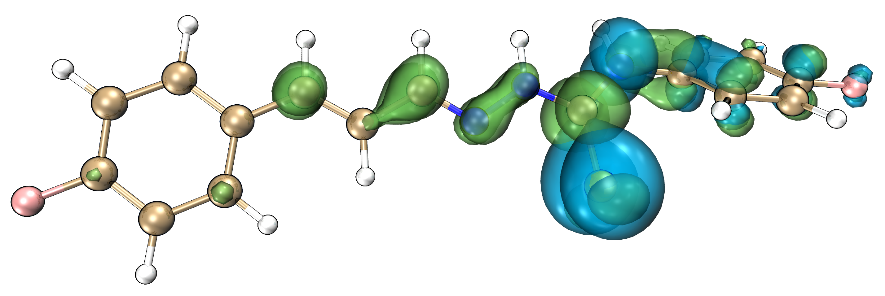** | **4.84 eV** |

**Table S14. Plot of hole+electron surfaces of main excitations of compound 5. Hole is the blue surface and electron is the green surface.**

| **Excitation** | **hole+electron** | **Energy** |
| --- | --- | --- |
| **S_0_→S_1_** | **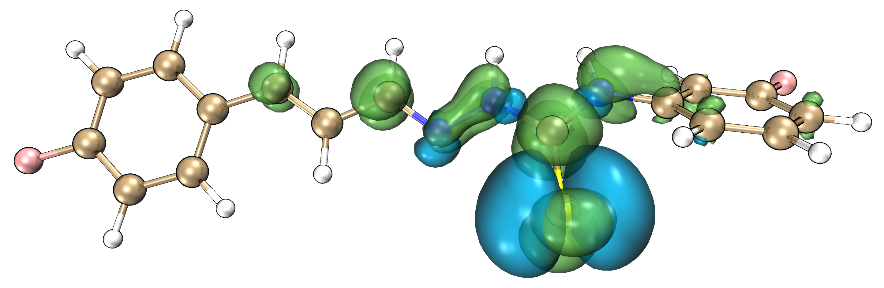** | **3.70 eV** |
| **S_0_→S_2_** | **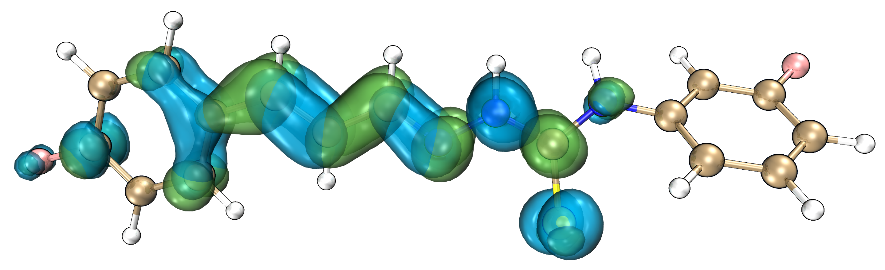** | **4.16 eV** |
| **S_0_→S_3_** | **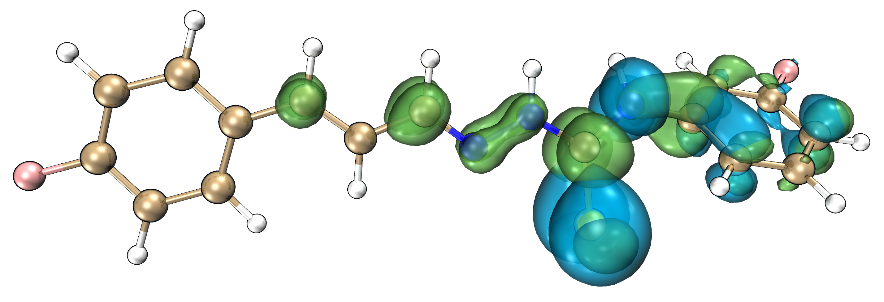** | **4.76 eV** |

**Table S15. Plot of hole+electron surfaces of main excitations of compound 6. Hole is the blue surface and electron is the green surface.**

| **Excitation** | **hole+electron** | **Energy** |
| --- | --- | --- |
| **S_0_→S_1_** | **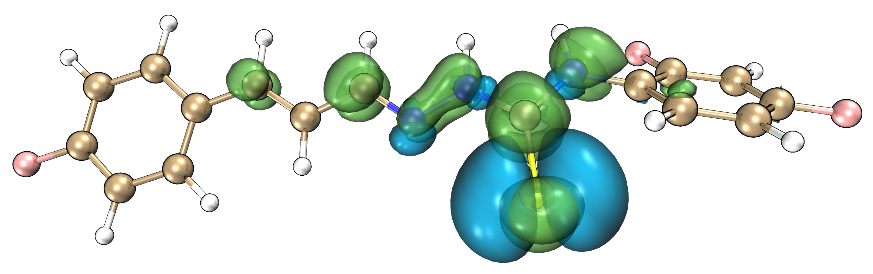** | **3.79 eV** |
| **S_0_→S_2_** | **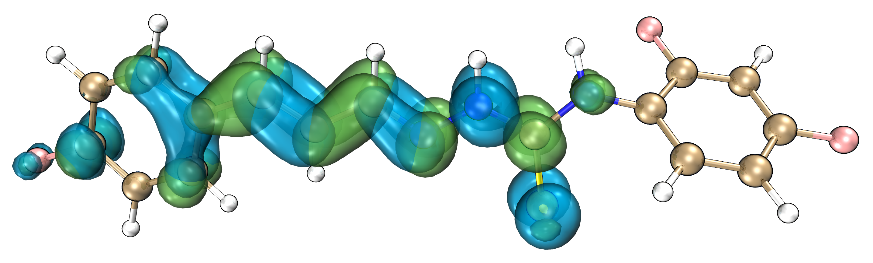** | **4.19 eV** |
| **S_0_→S_3_** | **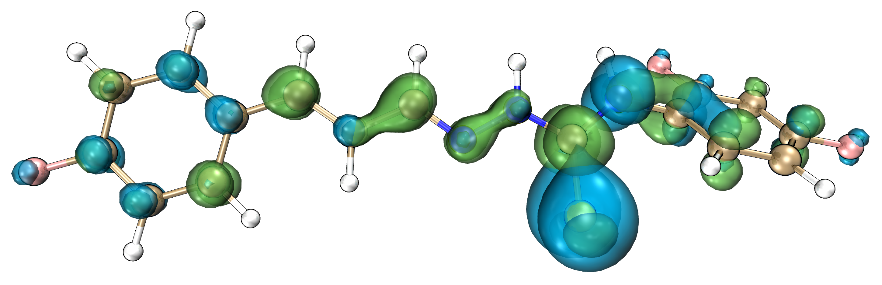** | **4.94 eV** |

**Table S16. Percentage of contribution of S atom to hole and MO contribution above 5% to formation of hole and electron of main excitation of compound 0.**

| **Excitation** | **%S to hole** | **MO** | **Type** | **Contribution** |
| --- | --- | --- | --- | --- |
| **S_0_→S_1_** | **89.37 %** | **HOMO-2** | **Hole** | **24.93 %** |
|  |  | **HOMO-1** | **Hole** | **72.24 %** |
|  |  | **LUMO** | **Electron** | **48.97 %** |
|  |  | **LUMO+1** | **Electron** | **32.53 %** |
| **S_0_→S_2_** | **11.36 %** | **HOMO** | **Hole** | **89.75 %** |
|  |  | **LUMO** | **Electron** | **89.98 %** |
| **S_0_→S_3_** | **49.25 %** | **HOMO-2** | **Hole** | **67.41 %** |
|  |  | **HOMO-1** | **Hole** | **24.20 %** |
|  |  | **LUMO** | **Electron** | **52.78 %** |
|  |  | **LUMO+1** | **Electron** | **37.45 %** |

**Table S17. Percentage of contribution of S atom to hole and MO contribution above 5% to formation of hole and electron of main excitation of compound 1.**

| **Excitation** | **%S to hole** | **MO** | **Type** | **Contribution** |
| --- | --- | --- | --- | --- |
| **S_0_→S_1_** | **11.93 %** | **HOMO-9** | **Hole** | **5.71 %** |
|  |  | **HOMO-5** | **Hole** | **5.75 %** |
|  |  | **HOMO-3** | **Hole** | **10.19 %** |
|  |  | **HOMO-1** | **Hole** | **5.09 %** |
|  |  | **HOMO** | **Hole** | **64.43 %** |
|  |  | **LUMO** | **Electron** | **79.68 %** |
|  |  | **LUMO+1** | **Electron** | **11.41 %** |
| **S_0_→S_2_** | **79.88 %** | **HOMO-2** | **Hole** | **25.41 %** |
|  |  | **HOMO-1** | **Hole** | **63.30 %** |
|  |  | **HOMO** | **Hole** | **5.10 %** |
|  |  | **LUMO** | **Electron** | **22.16 %** |
|  |  | **LUMO+1** | **Electron** | **34.73 %** |
|  |  | **LUMO+2** | **Electron** | **28.12 %** |
| **S_0_→S_3_** | **0.59 %** | **HOMO-9** | **Hole** | **39.33 %** |
|  |  | **HOMO-8** | **Hole** | **21.83 %** |
|  |  | **HOMO-5** | **Hole** | **22.16 %** |
|  |  | **HOMO** | **Hole** | **12.35 %** |
|  |  | **LUMO** | **Electron** | **80.72 %** |
|  |  | **LUMO+1** | **Electron** | **12.78 %** |
| **S_0_→S_4_** | **16.29 %** | **HOMO-3** | **Hole** | **11.57 %** |
|  |  | **HOMO-1** | **Hole** | **5.90 %** |
|  |  | **HOMO** | **Hole** | **71.76 %** |
|  |  | **LUMO** | **Electron** | **12.32 %** |
|  |  | **LUMO+1** | **Electron** | **76.87 %** |
|  |  | **LUMO+2** | **Electron** | **5.36 %** |

**Table S18. Percentage of contribution of S atom to hole and MO contribution above 5% to formation of hole and electron of main excitation of compound 2.**

| **Excitation** | **%S to hole** | **MO** | **Type** | **Contribution** |
| --- | --- | --- | --- | --- |
| **S_0_→S_1_** | **89.05 %** | **HOMO-2** | **Hole** | **23.28 %** |
|  |  | **HOMO-1** | **Hole** | **74.30 %** |
|  |  | **LUMO** | **Electron** | **15.18 %** |
|  |  | **LUMO+1** | **Electron** | **48.47 %** |
|  |  | **LUMO+2** | **Electron** | **5.74 %** |
|  |  | **LUMO+6** | **Electron** | **11.67 %** |
| **S_0_→S_2_** | **4.42 %** | **HOMO-9** | **Hole** | **6.03 %** |
|  |  | **HOMO-5** | **Hole** | **6.33 %** |
|  |  | **HOMO-3** | **Hole** | **12.41 %** |
|  |  | **HOMO** | **Hole** | **69.31 %** |
|  |  | **LUMO** | **Electron** | **86.09 %** |
|  |  | **LUMO+1** | **Electron** | **8.74 %** |
| **S_0_→S_3_** | **0.73 %** | **HOMO-9** | **Hole** | **36.75 %** |
|  |  | **HOMO-8** | **Hole** | **25.09 %** |
|  |  | **HOMO-5** | **Hole** | **21.96 %** |
|  |  | **HOMO** | **Hole** | **11.79 %** |
|  |  | **LUMO** | **Electron** | **78.11 %** |
|  |  | **LUMO+1** | **Electron** | **14.34 %** |
| **S_0_→S_4_** | **10.06 %** | **HOMO-3** | **Hole** | **13.76 %** |
|  |  | **HOMO** | **Hole** | **76.21 %** |
|  |  | **LUMO** | **Hole** | **12.56 %** |
|  |  | **LUMO+1** | **Electron** | **73.57 %** |
|  |  | **LUMO+2** | **Electron** | **6.50 %** |

**Table S19. Percentage of contribution of S atom to hole and MO contribution above 5% to formation of hole and electron of main excitation of compound 3.**

| **Excitation** | **%S to hole** | **MO** | **Type** | **Contribution** |
| --- | --- | --- | --- | --- |
| **S_0_→S_1_** | **3.75 %** | **HOMO-10** | **Hole** | **10.21 %** |
|  |  | **HOMO-9** | **Hole** | **5.43 %** |
|  |  | **HOMO-6** | **Hole** | **10.60 %** |
|  |  | **HOMO-5** | **Hole** | **7.03 %** |
|  |  | **HOMO-1** | **Hole** | **47.95 %** |
|  |  | **HOMO** | **Hole** | **9.89 %** |
|  |  | **LUMO** | **Electron** | **82.13 %** |
|  |  | **LUMO+1** | **Electron** | **11.49 %** |
| **S_0_→S_2_** | **5.39 %** | **HOMO-10** | **Hole** | **25.05 %** |
|  |  | **HOMO-9** | **Hole** | **26.35 %** |
|  |  | **HOMO-5** | **Hole** | **17.73 %** |
|  |  | **HOMO-2** | **Hole** | **5.75 %** |
|  |  | **HOMO-1** | **Hole** | **14.82 %** |
|  |  | **HOMO** | **Hole** | **5.76 %** |
|  |  | **LUMO** | **Electron** | **74.41 %** |
|  |  | **LUMO+1** | **Electron** | **15.92 %** |
| **S_0_→S_3_** | **79.97 %** | **HOMO-2** | **Hole** | **77.55 %** |
|  |  | **HOMO-1** | **Hole** | **10.03 %** |
|  |  | **HOMO** | **Hole** | **6.20 %** |
|  |  | **LUMO** | **Electron** | **23.73 %** |
|  |  | **LUMO+1** | **Electron** | **32.55 %** |
|  |  | **LUMO+2** | **Electron** | **29.49 %** |
| **S_0_→S_4_** | **4.75 %** | **HOMO-5** | **Hole** | **9.60 %** |
|  |  | **HOMO-1** | **Hole** | **56.24 %** |
|  |  | **HOMO** | **Hole** | **20.50 %** |
|  |  | **LUMO** | **Electron** | **13.16 %** |
|  |  | **LUMO+1** | **Electron** | **72.42 %** |

**Table S20. Percentage of contribution of S atom to hole and MO contribution above 5% to formation of hole and electron of main excitation of compound 4.**

| **Excitation** | **%S to hole** | **MO** | **Type** | **Contribution** |
| --- | --- | --- | --- | --- |
| **S_0_→S_1_** | **89.11 %** | **HOMO-2** | **Hole** | **22.76 %** |
|  |  | **HOMO-1** | **Hole** | **74.46 %** |
|  |  | **LUMO** | **Electron** | **49.08 %** |
|  |  | **LUMO+1** | **Electron** | **28.63 %** |
| **S_0_→S_2_** | **11.59 %** | **HOMO** | **Hole** | **89.36 %** |
|  |  | **LUMO** | **Electron** | **90.00 %** |
| **S_0_→S_3_** | **50.45 %** | **HOMO-2** | **Hole** | **67.02 %** |
|  |  | **HOMO-1** | **Hole** | **21.34 %** |
|  |  | **LUMO** | **Electron** | **53.67 %** |
|  |  | **LUMO+1** | **Electron** | **30.12 %** |

**Table S21. Percentage of contribution of S atom to hole and MO contribution above 5% to formation of hole and electron of main excitation of compound 5.**

| **Excitation** | **%S to hole** | **MO** | **Type** | **Contribution** |
| --- | --- | --- | --- | --- |
| **S_0_→S_1_** | **88.93 %** | **HOMO-2** | **Hole** | **20.56 %** |
|  |  | **HOMO-1** | **Hole** | **76.19 %** |
|  |  | **LUMO** | **Electron** | **50.86 %** |
|  |  | **LUMO+1** | **Electron** | **28.58 %** |
|  |  | **LUMO+15** | **Electron** | **5.05 %** |
| **S_0_→S_2_** | **11.66 %** | **HOMO-3** | **Hole** | **5.40 %** |
|  |  | **HOMO** | **Hole** | **89.76 %** |
|  |  | **LUMO** | **Electron** | **89.99 %** |
| **S_0_→S_3_** | **48.14 %** | **HOMO-2** | **Hole** | **71.71 %** |
|  |  | **HOMO-1** | **Hole** | **20.19 %** |
|  |  | **LUMO** | **Electron** | **52.84 %** |
|  |  | **LUMO+1** | **Electron** | **36.65 %** |

**Table S22. Percentage of contribution of S atom to hole and MO contribution above 5% to formation of hole and electron of main excitation of compound 6.**

| **Excitation** | **%S to hole** | **MO** | **Type** | **Contribution** |
| --- | --- | --- | --- | --- |
| **S_0_→S_1_** | **89.22 %** | **HOMO-2** | **Hole** | **9.94 %** |
|  |  | **HOMO-1** | **Hole** | **87.89 %** |
|  |  | **LUMO** | **Electron** | **48.86 %** |
|  |  | **LUMO+1** | **Electron** | **23.07 %** |
|  |  | **LUMO+5** | **Electron** | **6.20 %** |
| **S_0_→S_2_** | **11.87 %** | **HOMO** | **Hole** | **90.18 %** |
|  |  | **LUMO** | **Electron** | **90.17 %** |
| **S_0_→S_3_** | **44.31 %** | **HOMO-4** | **Hole** | **5.55 %** |
|  |  | **HOMO-3** | **Hole** | **6.75 %** |
|  |  | **HOMO-2** | **Hole** | **62.73 %** |
|  |  | **HOMO-1** | **Hole** | **9.92 %** |
|  |  | **HOMO** | **Hole** | **10.47 %** |
|  |  | **LUMO** | **Electron** | **48.91 %** |
|  |  | **LUMO+1** | **Electron** | **28.21 %** |

**Table S23. Plot of molecular orbitals (MO) surfaces that contributed to hole and electron of main excitations of compound 0.**

| **MO** | **Surface** |
| --- | --- |
| **HOMO-2** | **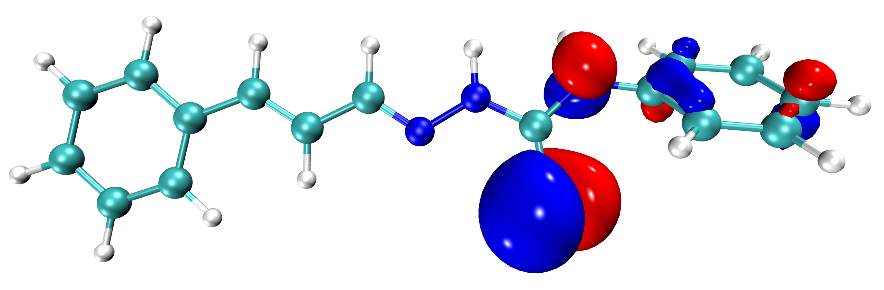** |
| **HOMO-1** | **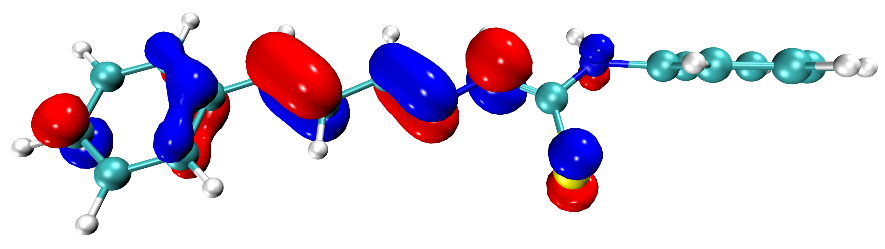** |
| **HOMO** |  |
| **LUMO** | **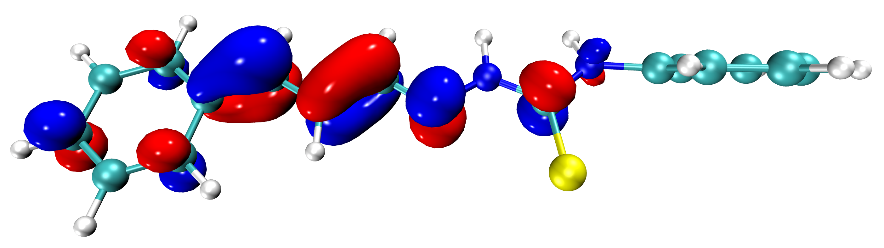** |
| **LUMO+1** | **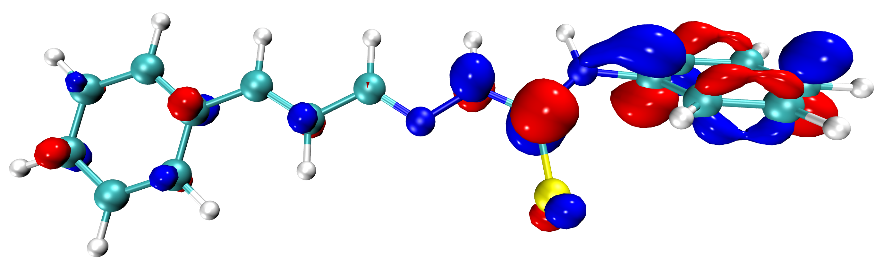** |

**Table S24. Plot of molecular orbitals (MO) surfaces that contributed to hole and electron of main excitations of compound 1.**

| **MO** | **Surface** |
| --- | --- |
| **HOMO-9** | **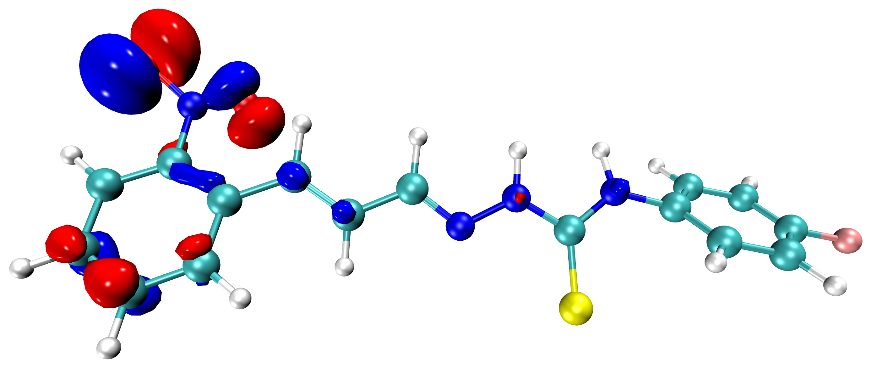** |
| **HOMO-8** | **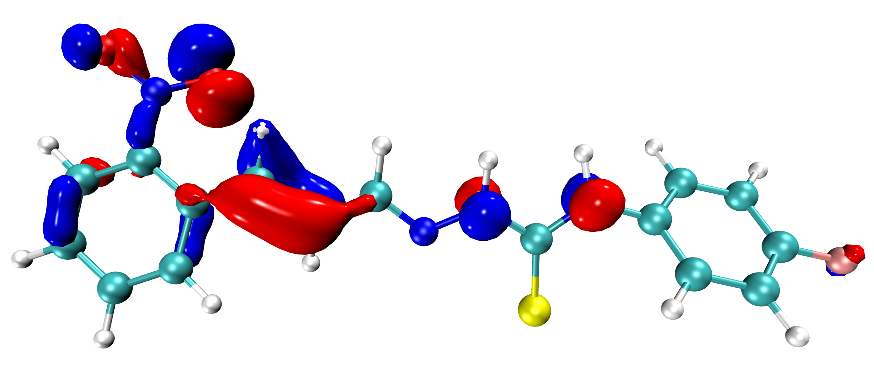** |
| **HOMO-5** | **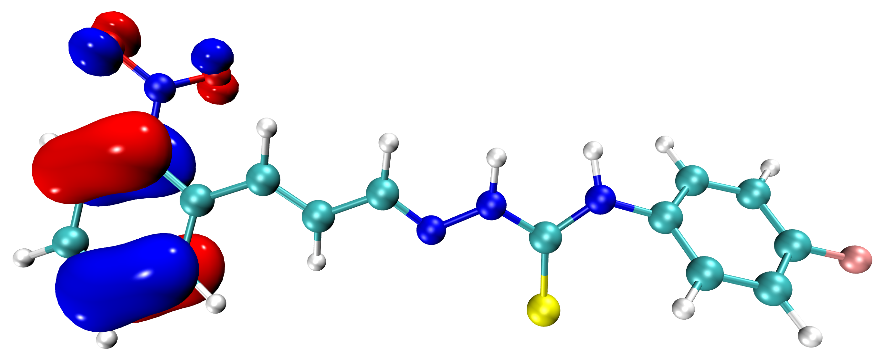** |
| **HOMO-3** | **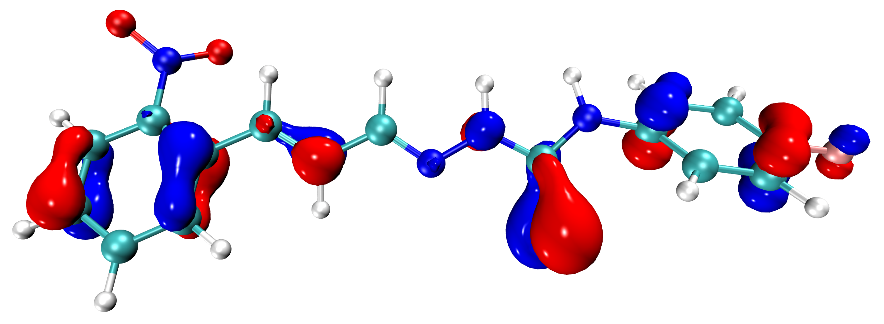** |
| **HOMO-2** | **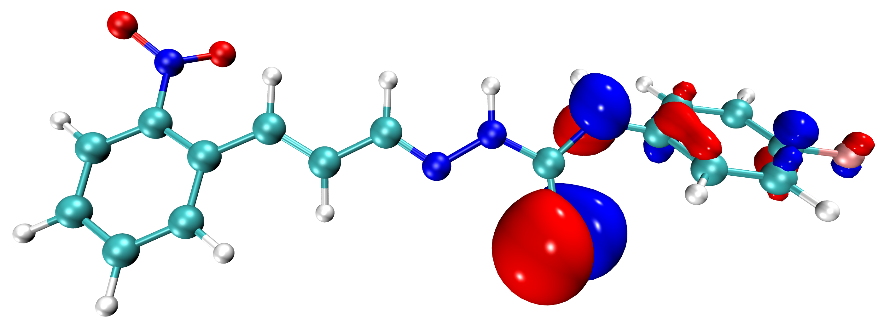** |
| **HOMO-1** | **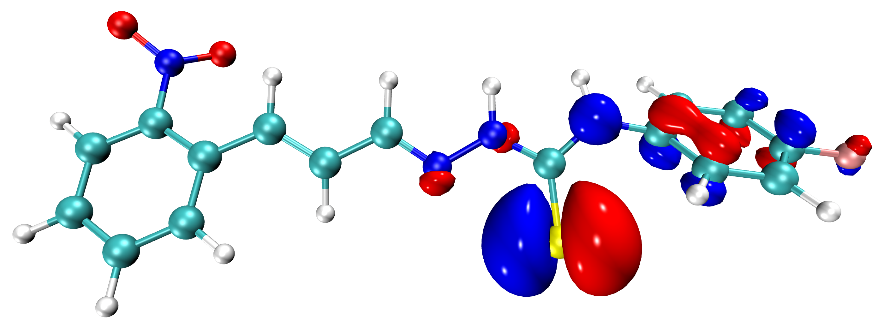** |
| **HOMO** |  |
| **LUMO** | **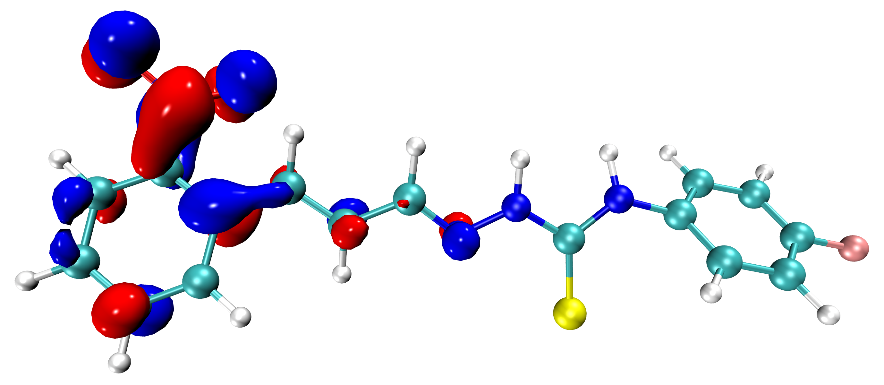** |
| **LUMO+1** | **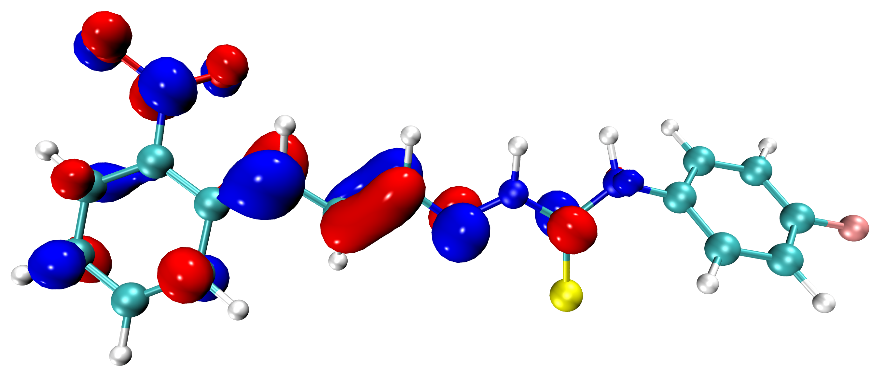** |
| **LUMO+2** | **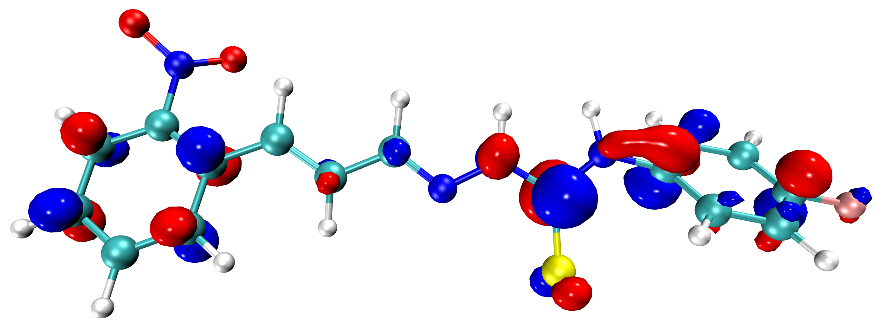** |

**Table S25. Plot of molecular orbitals (MO) surfaces that contributed to hole and electron of main excitations of compound 2.**

| **MO** | **Surface** |
| --- | --- |
| **HOMO-9** | **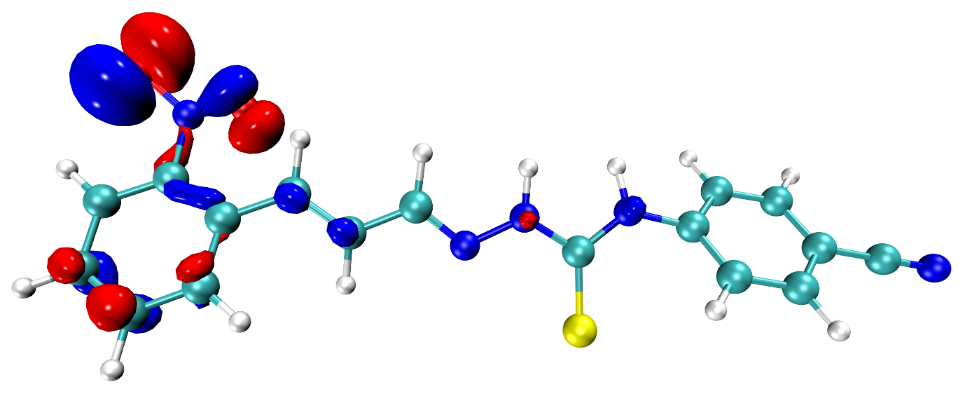** |
| **HOMO-8** | **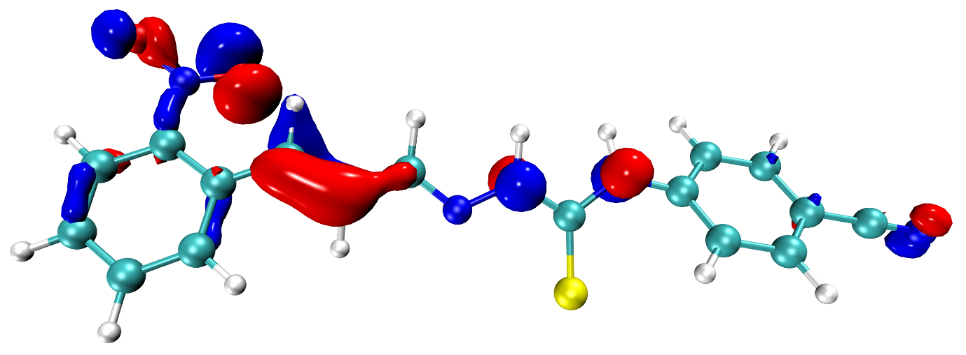** |
| **HOMO-5** | **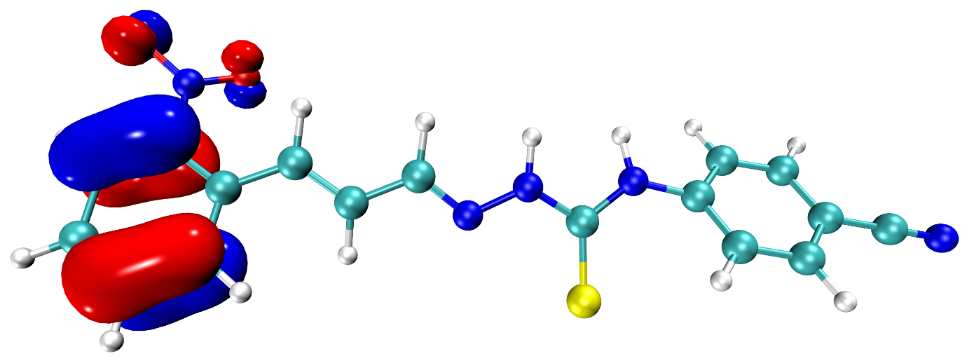** |
| **HOMO-3** | **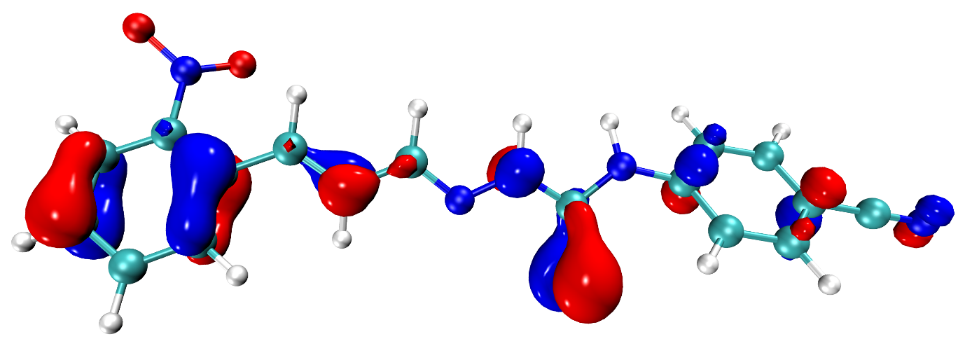** |
| **HOMO-2** | **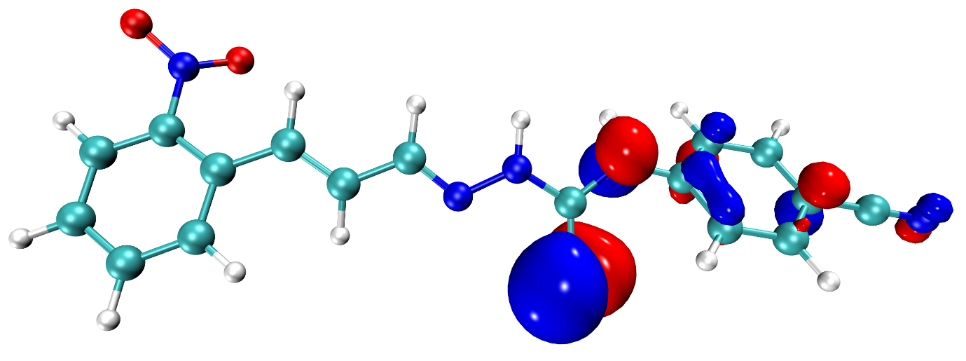** |
| **HOMO-1** | **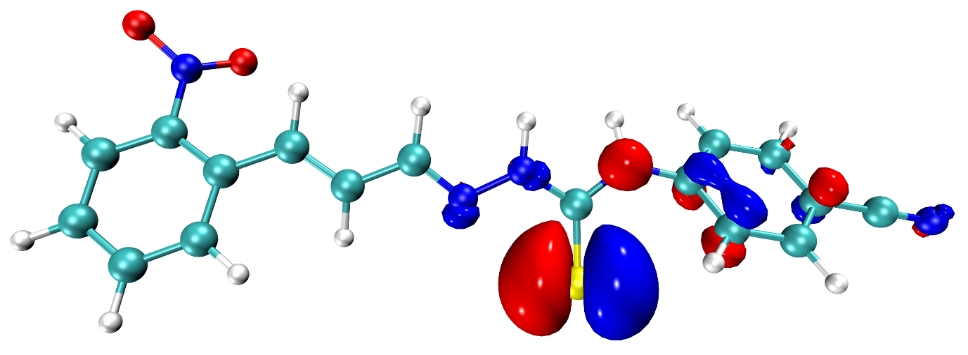** |
| **HOMO** | **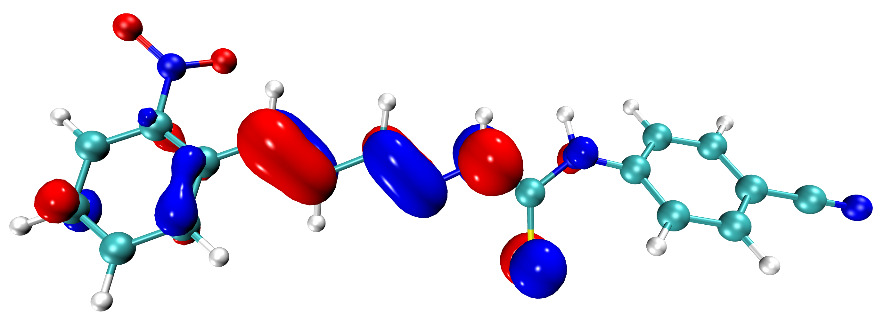** |
| **LUMO** | **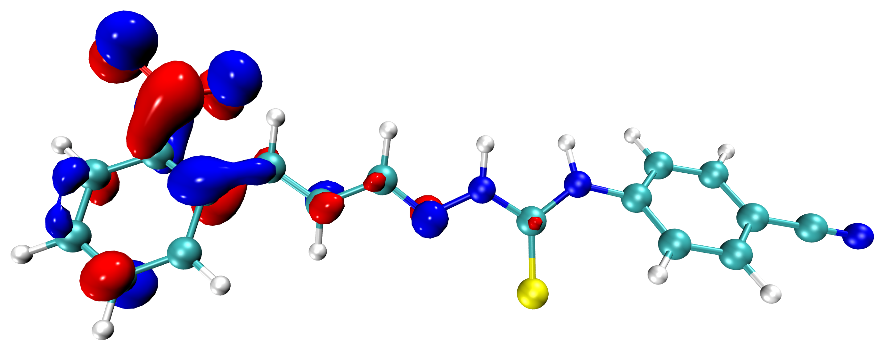** |
| **LUMO+1** | **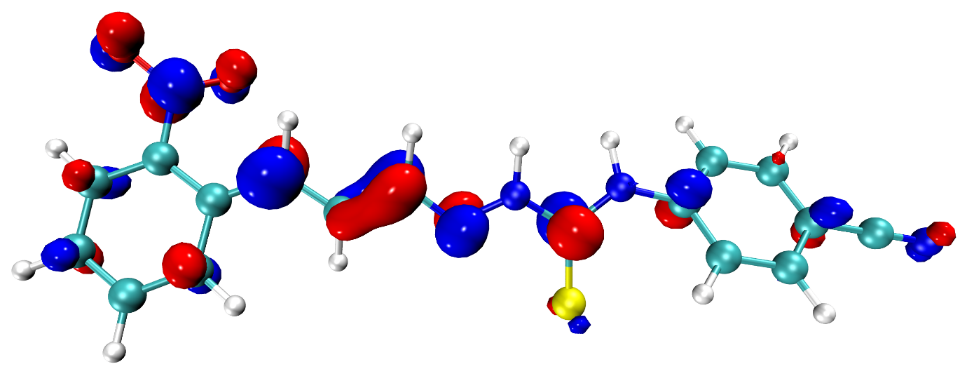** |
| **LUMO+2** | **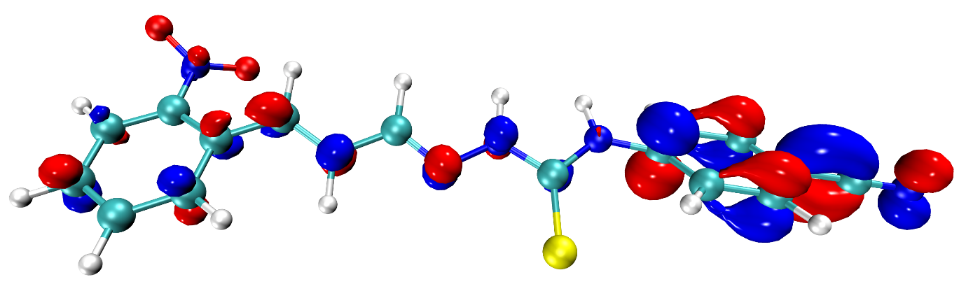** |

**Table S26. Plot of molecular orbitals (MO) surfaces that contributed to hole and electron of main excitations of compound 3.**

| **MO** | **Surface** |
| --- | --- |
| **HOMO-10** | **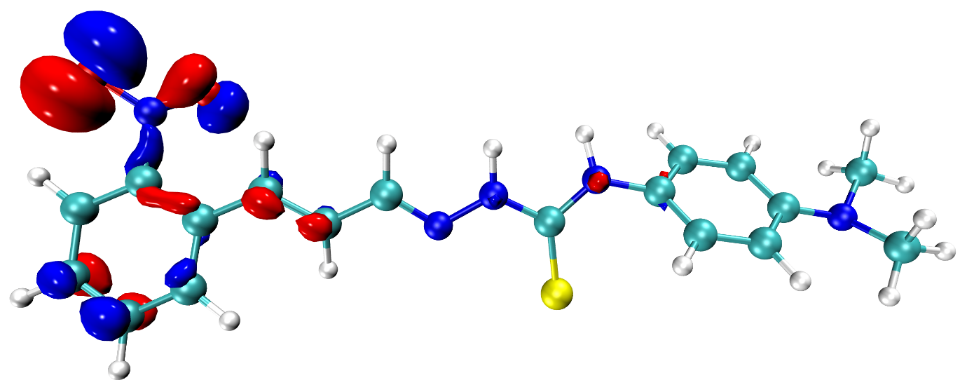** |
| **HOMO-9** | **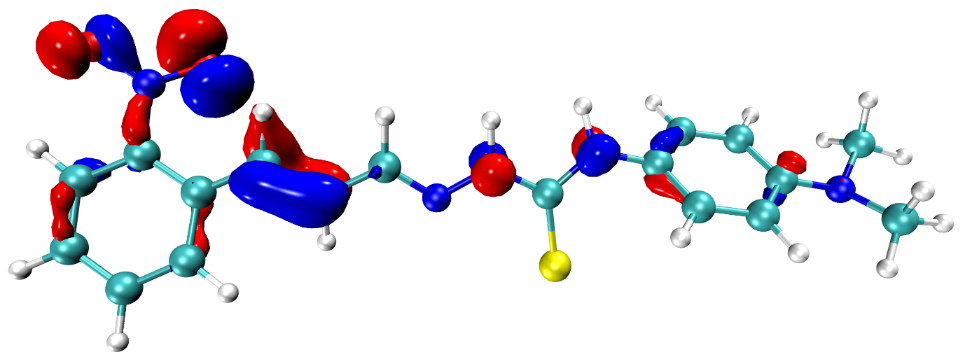** |
| **HOMO-6** | **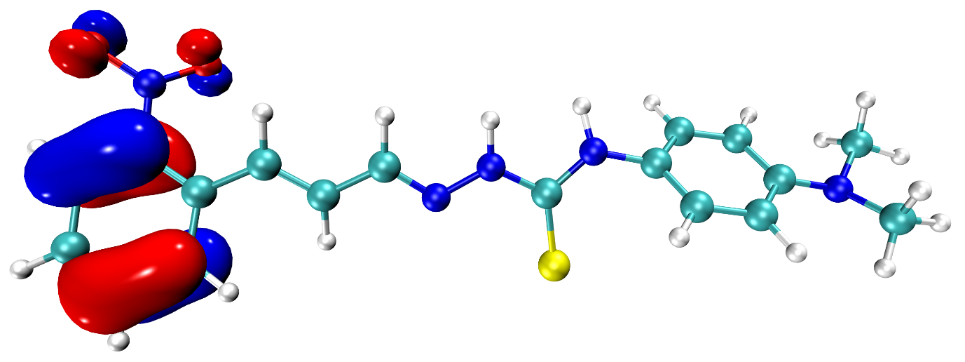** |
| **HOMO-5** | **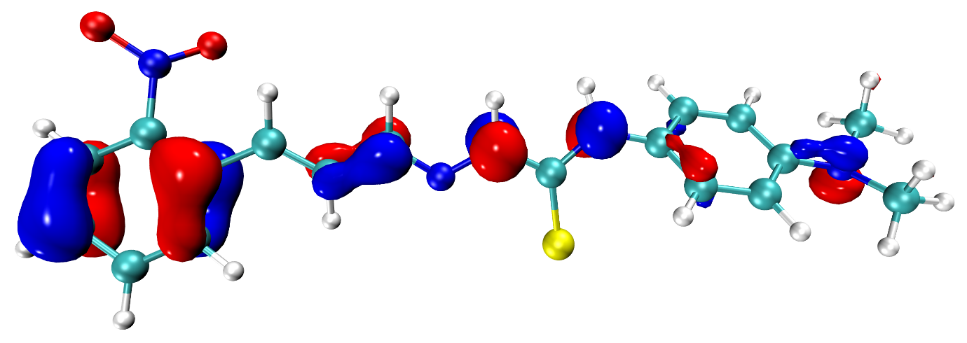** |
| **HOMO-2** | **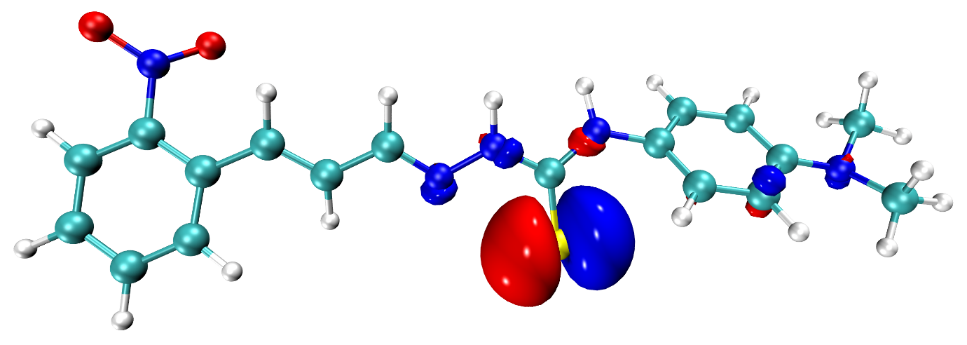** |
| **HOMO-1** | **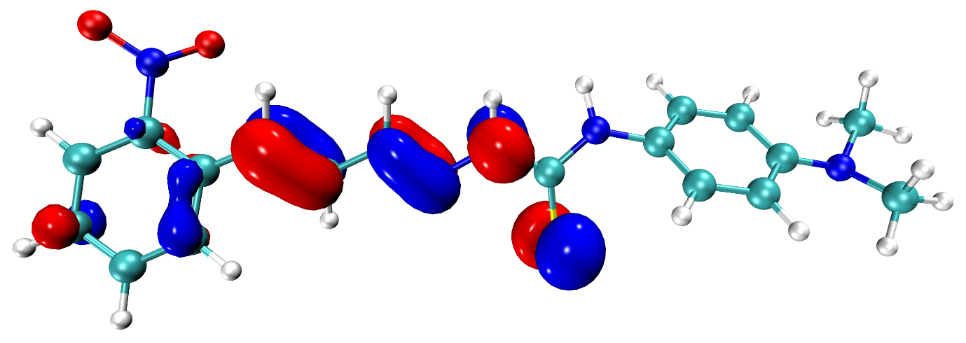** |
| **HOMO** | **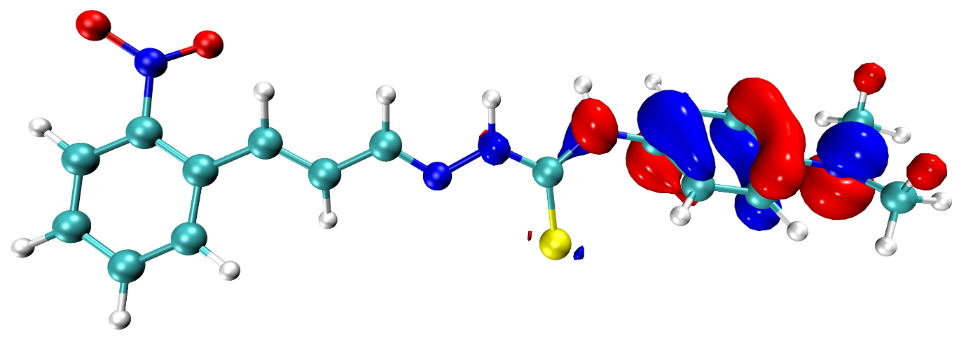** |
| **LUMO** | **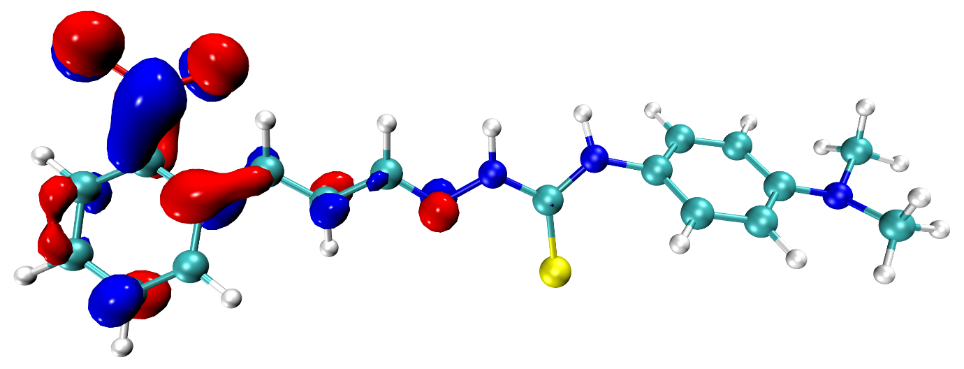** |
| **LUMO+1** | **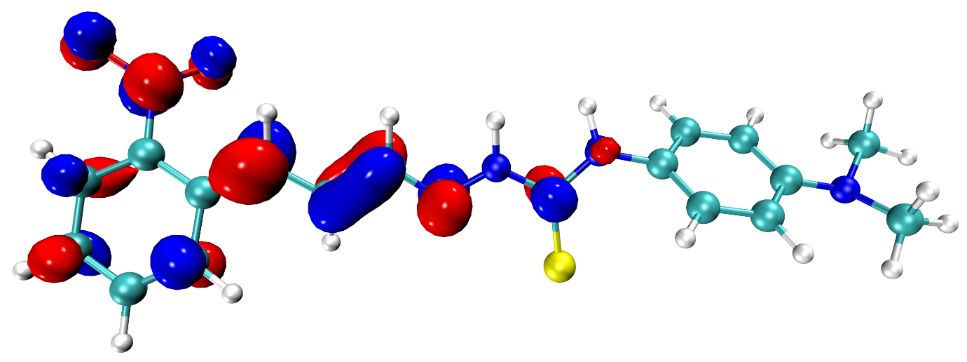** |
| **LUMO+2** | **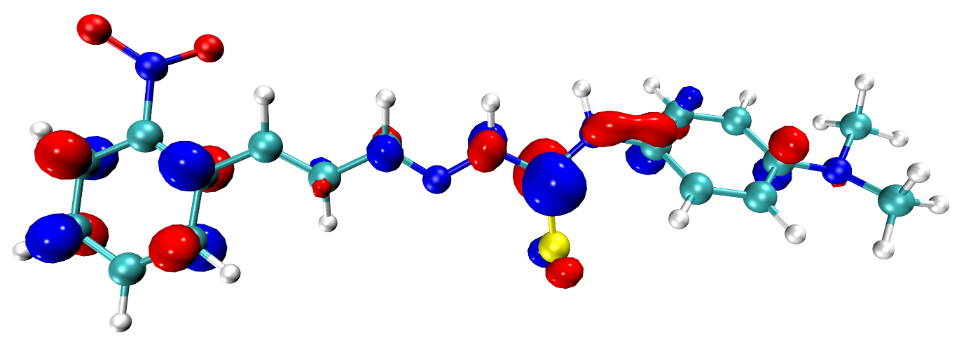** |

**Table S27. Plot of molecular orbitals (MO) surfaces that contributed to hole and electron of main excitations of compound 4.**

| **MO** | **Type** |
| --- | --- |
| **HOMO-2** | **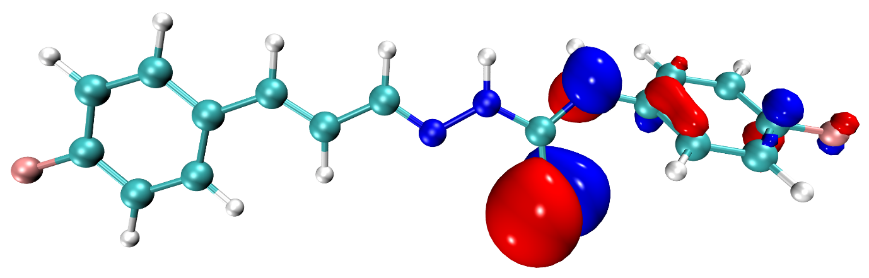** |
| **HOMO-1** | **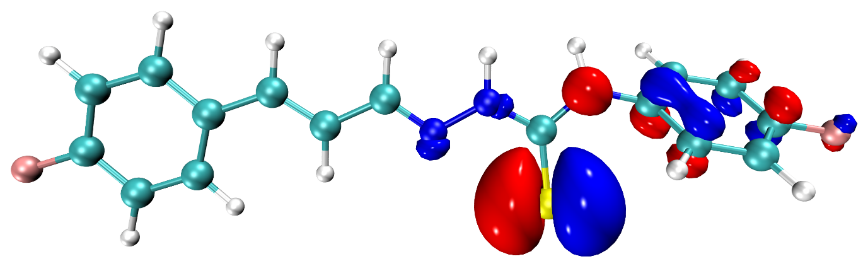** |
| **HOMO** | **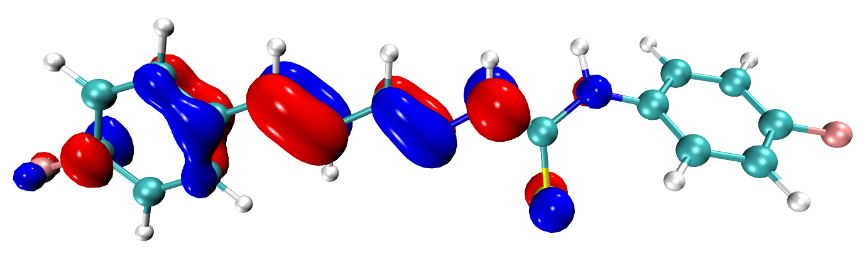** |
| **LUMO** | **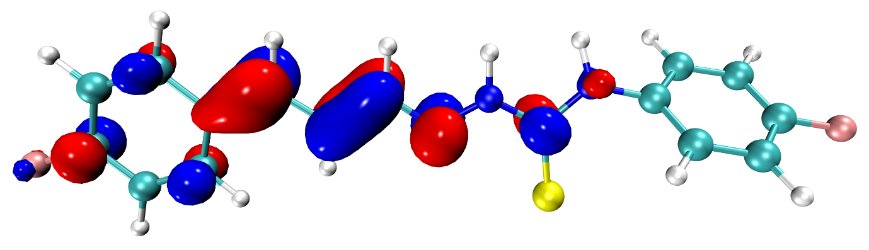** |
| **LUMO+1** | **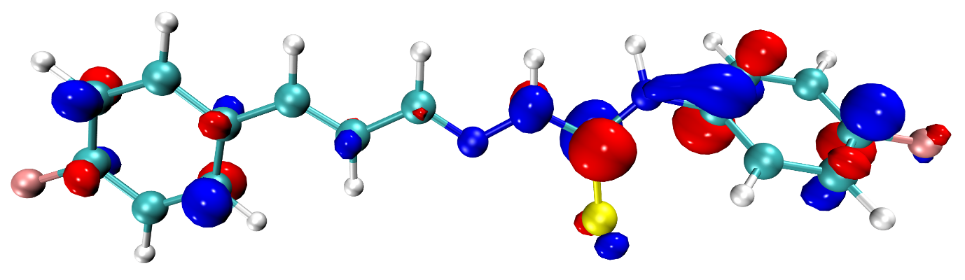** |

**Table S28. Plot of molecular orbitals (MO) surfaces that contributed to hole and electron of main excitations of compound 5.**

| **MO** | **Surface** |
| --- | --- |
| **HOMO-3** | **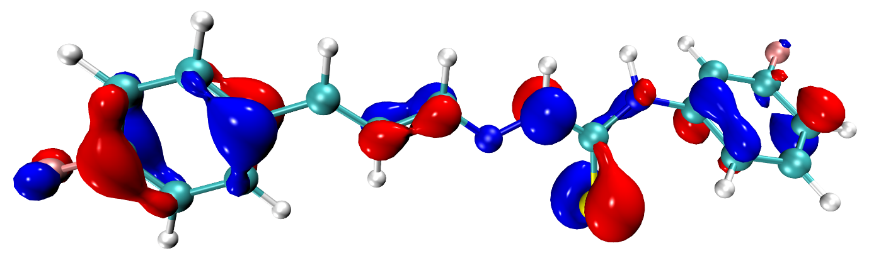** |
| **HOMO-2** | **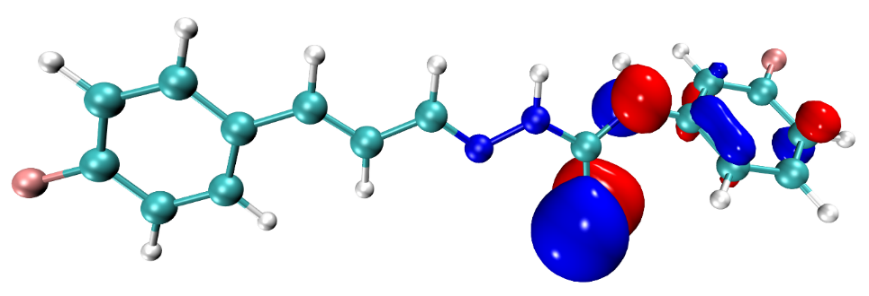** |
| **HOMO-1** | **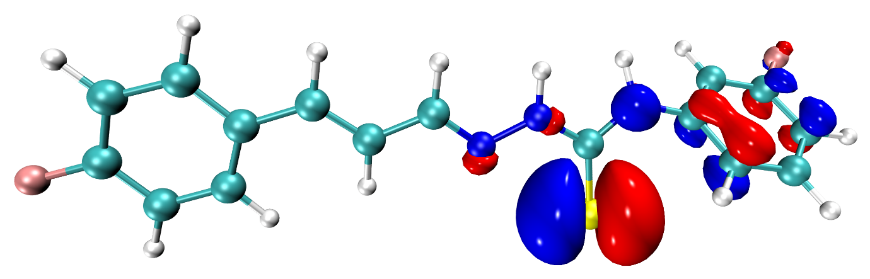** |
| **HOMO** | **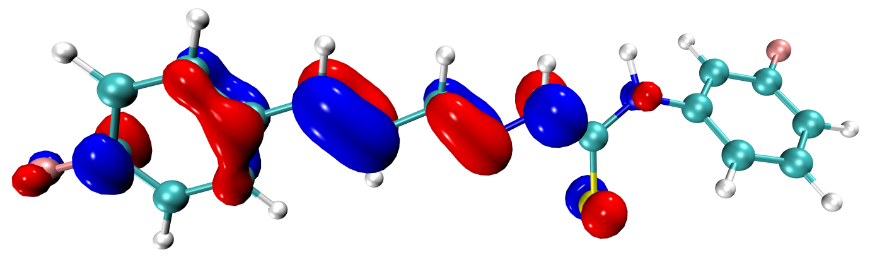** |
| **LUMO** | **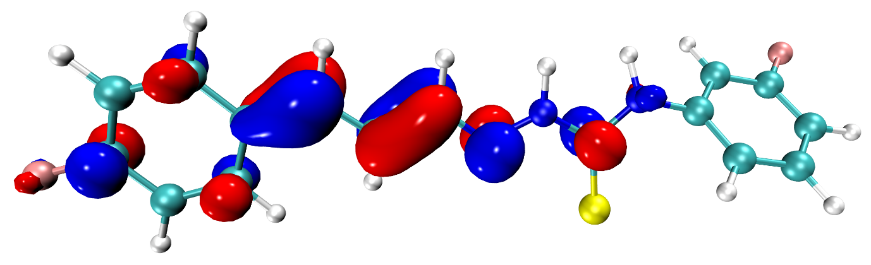** |
| **LUMO+1** |  |
| **LUMO+15** |  |

**Table S29. Plot of molecular orbitals (MO) surfaces that contributed to hole and electron of main excitations of compound 6.**

| **MO** | **Surface** |
| --- | --- |
| **HOMO-4** |  |
| **HOMO-3** |  |
| **HOMO-2** |  |
| **HOMO-1** |  |
| **HOMO** |  |
| **LUMO** |  |
| **LUMO+1** |  |
| **LUMO+5** |  |

**Table S30. Calculated thermochemistry data with B3LYP-D3/6-311+G(d,p)/SMD of benzoic acid (H-bza) and substituted benzoic acids (R-bza, X= *m*-NO_2_-, *p*-NO_2_-, *m*-F-, or *p*-F) of proton dissociation in water (SMD).**

| **Compound** | $\boldsymbol{G}_{\mathbf{aq}}^{\mathbf{*}}\boldsymbol{(AH)}$ | $\boldsymbol{G}_{\mathbf{aq}}^{\mathbf{*}}\boldsymbol{(}\boldsymbol{A}^{\boldsymbol{-}}\boldsymbol{)}$ | $\boldsymbol{G}_{\mathbf{aq}}^{\mathbf{*}}\boldsymbol{(}\boldsymbol{H}^{\boldsymbol{+}}\boldsymbol{)}$ | $\boldsymbol{\Delta}\boldsymbol{G}_{\boldsymbol{aq}}^{\boldsymbol{*}}$ |
| --- | --- | --- | --- | --- |
| **H-bza** | **-264109.613267** | **-263830.773145** | **-270.296808** | **8.54** |
| ***m*- NO_2_-bza** | **-392472.873755** | **-392198.648966** | **-270.296808** | **3.93** |
| ***p*-NO_2_-bza** | **-392478.317400** | **-392202.036263** | **-270.296808** | **5.98** |
| ***m*-F-bza** | **-326404.055126** | **-326126.878532** | **-270.296808** | **6.88** |
| ***p*-F-bza** | **-326408.166568** | **-326129.682244** | **-270.296808** | **8.19** |

**Table S31. Calculated (with B3LYP-D3/6-311+G(d,p)/SMD method) equilibrium constants (**$\boldsymbol{K}_{\boldsymbol{a}}$**),**$\boldsymbol{p}\boldsymbol{K}_{\boldsymbol{a}}$**, and Hammet constants (**$\boldsymbol{\sigma}_{\boldsymbol{X}}$**), besides experimental values of** $\boldsymbol{p}\boldsymbol{K}_{\boldsymbol{a}}$ **and** $\boldsymbol{\sigma}_{\boldsymbol{X}}$ **of benzoic acid (H-bza)** **and substituted benzoic acids (R-bza, R= *m*-NO_2_-, *p*-NO_2_-, *m*-F-, or *p*-F).**

| **Compound** | $\boldsymbol{K}_{\boldsymbol{a}}$ | ${\boldsymbol{p}\boldsymbol{K}_{\boldsymbol{a}}}_{\boldsymbol{calc}}$ | ${\boldsymbol{p}\boldsymbol{K}_{\boldsymbol{a}}}_{\boldsymbol{exp}}$ | ${\boldsymbol{\Delta p}\boldsymbol{K}_{\boldsymbol{a}}}_{\boldsymbol{calc-exp}}$ | ${\boldsymbol{\sigma}_{\boldsymbol{X}}}_{\boldsymbol{calc}}$ | ${\boldsymbol{\sigma}_{\boldsymbol{X}}}_{\boldsymbol{exp}}$ |
| --- | --- | --- | --- | --- | --- | --- |
| **H-bza** | **5.41 x 10^-07^** | **6.27** | **4.19** | **2.08** | **0.00** | **0.00** |
| ***m*- NO_2_-bza** | **1.31 x 10^-03^** | **2.88** | **3.49** | **-0.61** | **3.38** | **0.71** |
| ***p*-NO_2_-bza** | **4.08 x 10^-05^** | **4.39** | **3.43** | **0.96** | **1.88** | **0.78** |
| ***m*-F-bza** | **8.99 x 10^-06^** | **5.05** | **3.87** | **1.18** | **1.22** | **0.34** |
| ***p*-F-bza** | **9.87 x 10^-07^** | **6.00** | **4.14** | **1.86** | **0.26** | **0.06** |

**Table S32. Calculated thermochemistry data with B3LYP-D3/6-311+G(d,p)/SMD methodof compounds 1-6 and hypothetical compound 0 (without substituting groups) proton dissociation in water.**

| **Compound** | $\boldsymbol{G}_{\mathbf{aq}}^{\mathbf{*}}\boldsymbol{(SH)}$ | $\boldsymbol{G}_{\mathbf{aq}}^{\mathbf{*}}\boldsymbol{(}\boldsymbol{S}^{\boldsymbol{-}}\boldsymbol{)}$ | $\boldsymbol{G}_{\mathbf{aq}}^{\mathbf{*}}\boldsymbol{(}\boldsymbol{H}^{\boldsymbol{+}}\boldsymbol{)}$ |
| --- | --- | --- | --- |
| **0** | **-741211.801723** | **-740918.993868** | **-270.296808** |
| **1** | **-931876.239758** | **-931585.950098** | **-270.296808** |
| **2** | **-927482.603982** | **-927194.892759** | **-270.296808** |
| **3** | **-953631.239229** | **-953338.802232** | **-270.296808** |
| **4** | **-865808.944721** | **-865515.728985** | **-270.296808** |
| **5** | **-865809.164349** | **-865517.967938** | **-270.296808** |
| **6** | **-928103.732338** | **-927814.729700** | **-270.296808** |

**Table S33. Calculated relative Gibbs free energy (**${\boldsymbol{\Delta}\boldsymbol{G}}_{\boldsymbol{eq}}$**) with B3LYP-D3/6-311+G(d,p)/SMD method of compounds 1-6proton dissociation equilibrium between sensors and anions (F^-^, AcO^-^, Br^-^, Cl^-^, HSO_4_^-^, ClO4^-^, CN^-^and SCN^-^).**

| **Compound** | **F^-^** | **AcO^-^** | **Br^-^** | **Cl^-^** | **HSO_4_^-^** | **ClO_4_^-^** | **CN^-^** | **SCN^-^** |
| --- | --- | --- | --- | --- | --- | --- | --- | --- |
| **0** | **7.77** | **12.70** | **23.59** | **25.88** | **38.31** | **45.82** | **5.78** | **20.67** |
| **1** | **5.25** | **10.18** | **21.08** | **23.37** | **35.79** | **43.30** | **3.26** | **18.16** |
| **2** | **2.67** | **7.60** | **18.50** | **20.79** | **33.21** | **40.72** | **0.68** | **15.58** |
| **3** | **7.40** | **12.33** | **23.22** | **25.51** | **37.94** | **45.45** | **5.41** | **20.30** |
| **4** | **8.17** | **13.11** | **24.00** | **26.29** | **38.72** | **46.23** | **6.19** | **21.08** |
| **5** | **6.16** | **11.09** | **21.98** | **24.27** | **36.70** | **44.21** | **4.17** | **19.06** |
| **6** | **3.96** | **8.89** | **19.79** | **22.08** | **34.50** | **42.02** | **1.98** | **16.87** |

**Table S34. Calculated equilibrium constants (**$\boldsymbol{K}_{\boldsymbol{eq}}$**) with B3LYP-D3/6-311+G(d,p)/SMD method of compounds 1-6 proton dissociation equilibrium** **between sensors and anions (F^-^, AcO^-^, Br^-^, Cl^-^, HSO_4_^-^, ClO4^-^, CN^-^and SCN^-^).**

| **Compound** | **F^-^** | **AcO^-^** | **Br^-^** | **Cl^-^** |
| --- | --- | --- | --- | --- |
| **0** | **2.01 x 10^-6^** | **4.84 x 10^-10^** | **4.93 x 10^-18^** | **1.03 x 10^-19^** |
| **1** | **1.41 x 10^-4^** | **3.40 x 10^-8^** | **3.47 x 10^-16^** | **7.26 x 10^-18^** |
| **2** | **1.10 x 10^-2^** | **2.65 x 10^-6^** | **2.70 x 10^-14^** | **5.65 x 10^-16^** |
| **3** | **3.76 x 10^-6^** | **9.06 x 10^-10^** | **9.23 x 10^-18^** | **1.93 x 10^-19^** |
| **4** | **1.01 x 10^-6^** | **2.43 x 10^-10^** | **2.48 x 10^-18^** | **5.18 x 10^-20^** |
| **5** | **3.06 x 10^-5^** | **7.36 x 10^-9^** | **7.50 x 10^-17^** | **1.57 x 10^-18^** |
| **6** | **1.24 x 10^-3^** | **2.99 x 10^-7^** | **3.05 x 10^-15^** | **6.38 x 10^-17^** |
| **Compound** | **HSO_4_^-^** | **ClO_4_^-^** | **CN^-^** | **SCN^-^** |
| **0** | **7.96 x 10^-29^** | **2.46 x 10^-34^** | **5.75 x 10^-5^** | **6.86 x 10^-16^** |
| **1** | **5.59 x 10^-27^** | **1.73 x 10^-32^** | **4.05 x 10^-3^** | **4.82 x 10^-14^** |
| **2** | **4.36 x 10^-25^** | **1.35 x 10^-30^** | **3.15 x 10^-1^** | **3.75 x 10^-12^** |
| **3** | **1.49 x 10^-28^** | **4.60 x 10^-34^** | **1.08 x 10^-4^** | **1.28 x 10^-15^** |
| **4** | **3.99 x 10^-29^** | **1.23 x 10^-34^** | **2.89 x 10^-5^** | **3.44 x 10^-16^** |
| **5** | **1.21 x 10^-27^** | **3.74 x 10^-33^** | **8.75 x 10^-4^** | **1.04 x 10^-14^** |
| **6** | **4.92 x 10^-26^** | **1.52 x 10^-31^** | **3.56 x 10^-2^** | **4.24 x 10^-13^** |

**Table-S35: Calculated relative Gibbs free energy (Δ*G*) with B3LYP-D3/6-311+G(d,p)/SMD method of interactions between sensors 1-6 and anions F^-^, CN^-^, and AcO^-^.**

|  | **Δ*G* (kcal.mol^-1^)** | | |
| --- | --- | --- | --- |
| **Compound** | **F^-^** | **CN^-^** | **AcO^-^** |
| **1** | **-6.64** | **1.70** | **0.25** |
| **2** | **-7.57** | **-0.03** | **-0.04** |
| **3** | **-5.94** | **2.01** | **2.60** |
| **4** | **-5.26** | **2.61** | **1.89** |
| **5** | **-5.21** | **2.06** | **2.22** |
| **6** | **-6.50** | **1.85** | **1.60** |

**Table-S36: Calculated relative enthalpy energies (Δ*H*) with B3LYP-D3/6-311+G(d,p)/SMD method of interactions between sensors 1-6 and anions F^-^, CN^-^, and AcO^-^.**

|  | **Δ*H* (kcal.mol^-1^)** | | |
| --- | --- | --- | --- |
| **Compound** | **F^-^** | **CN^-^** | **AcO^-^** |
| **1** | **-13.86** | **-6.30** | **-10.17** |
| **2** | **-14.72** | **-6.82** | **-10.84** |
| **3** | **-13.20** | **-6.06** | **-9.51** |
| **4** | **-13.49** | **-6.33** | **-9.79** |
| **5** | **-13.58** | **-6.32** | **-9.98** |
| **6** | **-13.92** | **-6.54** | **-10.15** |

**Table-S37: AIM data (calculated with B3LYP-D3/6-311+G(d,p)/SMD method) of H-bonds between sensors 1-6 and anions F^-^, CN^-^, and AcO^-^.**

| **Compound** | **Anion** | **BCP** | **ρ(r)** | **𝛻^2^ρ(r)** | **ε** | ***V*(r)** |
| --- | --- | --- | --- | --- | --- | --- |
| **1** | **F^-^** | ***a*** | **0.0423** | **+0.1430** | **0.0082** | **-0.0410** |
|  |  | ***b*** | **0.0466** | **+0.1525** | **0.0065** | **-0.0463** |
|  | **AcO^-^** | ***a*** | **0.0396** | **+0.1171** | **0.0465** | **-0.0334** |
|  |  | ***b*** | **0.0380** | **+0.1127** | **0.0456** | **-0.0315** |
|  | **CN^-^** | ***a*** | **0.0258** | **+0.0599** | **0.0115** | **-0.0148** |
|  |  | ***b*** | **0.0189** | **+0.0488** | **0.0465** | **-0.0096** |
| **2** | **F^-^** | ***a*** | **0.0454** | **+0.1501** | **0.0091** | **-0.0449** |
|  |  | ***b*** | **0.0476** | **+0.1550** | **0.0088** | **-0.0477** |
|  | **AcO^-^** | ***a*** | **0.0401** | **+0.1173** | **0.0387** | **-0.0339** |
|  |  | ***b*** | **0.0404** | **+0.1164** | **0.0406** | **-0.0342** |
|  | **CN^-^** | ***a*** | **0.0303** | **+0.0637** | **0.0140** | **-0.0187** |
|  |  | ***b*** | **0.0160** | **+0.0429** | **0.1156** | **-0.0077** |
| **3** | **F^-^** | ***a*** | **0.0402** | **+0.1375** | **0.0089** | **-0.0383** |
|  |  | ***b*** | **0.0468** | **+0.1530** | **0.0052** | **-0.0466** |
|  | **AcO^-^** | ***a*** | **0.0373** | **+0.1130** | **0.0461** | **-0.0307** |
|  |  | ***b*** | **0.0383** | **+0.1130** | **0.0461** | **-0.0317** |
|  | **CN^-^** | ***a*** | **0.0204** | **+ 0.0521** | **0.0266** | **-0.0106** |
|  |  | ***b*** | **0.0238** | **+0.5664** | **0.0104** | **-0.0132** |
| **4** | **F^-^** | ***a*** | **0.0443** | **+0.1484** | **0.0054** | **-0.0436** |
|  |  | ***b*** | **0.0430** | **+0.1436** | **0.0079** | **-0.0416** |
|  | **AcO^-^** | ***a*** | **0.0387** | **+0.1155** | **0.0473** | **-0.0323** |
|  |  | ***b*** | **0.0382** | **+0.1131** | **0.0464** | **-0.0316** |
|  | **CN^-^** | ***a*** | **0.0281** | **+0.0622** | **0.0117** | **-0.0168** |
|  |  | ***b*** | **0.0157** | **+0.0419** | **0.1025** | **-0.0075** |
| **5** | **F^-^** | ***a*** | **0.0450** | **+0.1499** | **0.0081** | **-0.0444** |
|  |  | ***b*** | **0.0438** | **+0.1458** | **0.0103** | **-0.0427** |
|  | **AcO^-^** | ***a*** | **0.0386** | **+0.1151** | **0.0442** | **-0.0321** |
|  |  | ***b*** | **0.0387** | **+0.1139** | **0.0447** | **-0.0322** |
|  | **CN^-^** | ***a*** | **0.0284** | **+0.0625** | **0.0127** | **-0.0171** |
|  |  | ***b*** | **0.0163** | **+0.0435** | **0.0954** | **-0.0079** |
| **6** | **F^-^** | ***a*** | **0.0452** | **+0.1501** | **0.0075** | **-0.0446** |
|  |  | ***b*** | **0.0436** | **+0.1451** | **0.0093** | **-0.0424** |
|  | **AcO^-^** | ***a*** | **0.0395** | **+0.1167** | **0.0469** | **-0.0333** |
|  |  | ***b*** | **0.0389** | **+0.1142** | **0.0467** | **-0.0324** |
|  | **CN^-^** | ***a*** | **0.0274** | **+0.0613** | **0.0104** | **-0.0161** |
|  |  | ***b*** | **0.0168** | **+0.0444** | **0.0734** | **-0.0082** |
